# Design, Synthesis, and Biological Evaluation of Proteolysis Targeting Chimeras (PROTACs) for the Dual Degradation of IGF-1R and Src

**DOI:** 10.3390/molecules25081948

**Published:** 2020-04-23

**Authors:** Sudhakar Manda, Na Keum Lee, Dong-Chan Oh, Jeeyeon Lee

**Affiliations:** 1 College of Pharmacy, Research Institute of Pharmaceutical sciences, Seoul National University, 1 Gwanak-ro, Gwanak-gu, Seoul 08826, Korea; sudhakariiim@gmail.com (S.M.); nklee12@snu.ac.kr (N.K.L.); 2 Natural Products Research Institute, College of Pharmacy, Seoul National University, Seoul 08826, Korea; dongchanoh@snu.ac.kr

**Keywords:** PROTACs, anticancer activity, protein degradation, IGF-1R, Src

## Abstract

A focused PROTAC library was developed to degrade both IGF-1R and Src proteins, which are associated with various cancers. PROTACs with IGF-1R and Src degradation potentials were synthesized by tethering different inhibitor warhead units and the E3 ligase (CRBN) recruiting-pomalidomide with various linkers. The designed PROTACs 12a–b inhibited the proliferation and migration of MCF7 and A549 cancer cells with low micromolar potency (1–5 μM) in various cellular assays.

## 1. Introduction

For the past two decades, targeted protein degradation strategies have been explored widely to develop treatments for various diseases, particularly for cancers [1,2,3,4]. Proteolysis targeting chimeras (PROTACs) have emerged as a novel therapeutic strategy in drug discovery for targeted protein degradation [4,5,6]. PROTACs are heterobifunctional molecules that possess one warhead ligand that binds to the target protein of interest (POI), and a second ligand that recruits an E3 ligase system, which is connected by a chemical linker. The recruitment of the E3 ligase to the target protein results in ubiquitination and the subsequent degradation of the target protein by the proteasome, which is a distinct mechanism from the occupancy-driven modulation of enzyme function by conventional small-molecule inhibitors. Initially described by Crews and Deshaies in 2001, PROTACs have been successfully applied to numerous target proteins [7,8,9,10,11,12]. As opposed to the stoichiometric binding of drugs that prevent the enzymatic activity of target proteins, PROTACs need to transiently bind to their targets for degradation; this is promising for the potential to address therapeutic challenges in targeting intractable proteins in the current drug discovery [13]. In addition, they can impede the feedback-mediated increased expression of the target proteins that often result in pharmacological inefficacy by small-molecule inhibitors [14,15].

Several E3 ligases have been used in PROTAC technology to degrade recruited target proteins, which include β-TRCP, MDM2 and cIAP [7,8,9]. In addition, von Hippel-Lindau (VHL) and cereblon (CRBN) have been extensively studied in relation to PROTAC-mediated protein degradation [2,16,17,18]. Cereblon (CRBN), which is a part of a cullin-RING ubiquitin ligase complex, was identified as the target of thalidomide and its derivatives [19]. These phthalimide immunomodulatory drugs (IMiDs), including lenalidomide, pomalidomide, and thalidomide, play a pivotal role in the treatment of multiple myeloma and now serve as potent binders of the CRBN. To date, PROTACs have been successfully applied to various target proteins with different cellular locations, including estrogen and androgen receptors, BET proteins, tau protein, FKBP12, and kinases [2,11,12,17,20,21]. In particular, PROTACs, which hijack various cellular kinases, have resulted in effective target degradations [22,23,24,25,26]. Furthermore, the enhanced selectivity of kinase degraders, as compared to their parental kinase inhibitors, were implicated as having potential applications for clinical study [23,27]. Currently, many efforts are underway in medicinal chemistry to convert previously ineffective inhibitors to selective PROTACs for a next-generation drug platform.

The insulin-like growth factor 1 receptor (IGF-1R) is a membrane receptor tyrosine kinase that is implicated in several cancers, including prostate, breast, and lung cancers [28,29,30]. IGF-1R plays a key role in the proliferation, transformation and survival of various cancer cells. Frequently, its anti-apoptotic properties result in resistance to cytotoxic chemotherapeutic drugs or radiotherapy. For these reasons, the IGF-1R signaling pathway has been a major target for the development of anticancer agents [31,32]. Src, known as proto-oncogene tyrosine-protein kinase, is also associated with cancer cell survival and resistance to targeted anticancer therapies [33]. In particular, Src activation is related to the resistance of many anti-IGF-1R therapeutics. Indeed, a combined inhibition of IGF-1R and the Src family kinases have been shown to enhance antitumor effects in various cancers by decreasing the activated survival pathways [34,35]. We have also shown that the dual inhibition of IGF-1R and Src could be a viable approach to develop effective anticancer therapies to overcome resistance [36].

**Figure 1:**
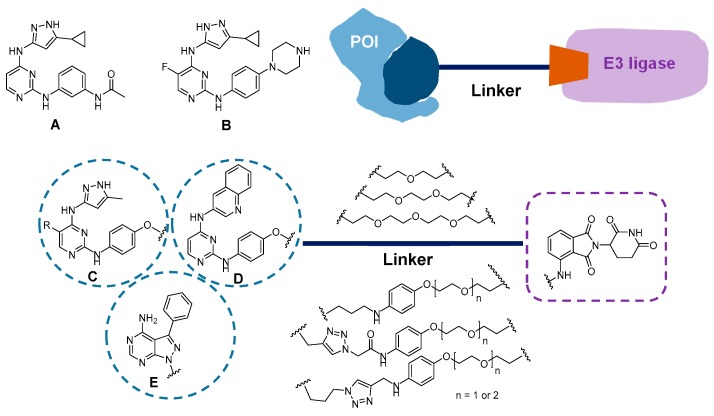
Chemical structures of representative Src inhibitor **A**, IGF-1R inhibitors **B**, and a schematic diagram of PROTACs for IGF-1R and Src.

In this study, we report the design and synthesis of IGF-1R/Src dual degraders using the PROTAC strategy (Figure 1). The (5-cyclopropyl-1*H*-pyrazol-3-yl)pyrimidine-2,4-diamine (A) is known as a potent c-Src inhibitor with an IC_50_ value of 720 nM from the enzyme inhibition assay [37,38]. RBx10080307 (B) is known as an IGF-1R inhibitor with a cell free IC_50_ value of 277 nM [39,40]. A common structure, *N*^2^-phenyl-*N*^4^-(1*H*-pyrazol-3-yl)pyrimidine-2,4-diamine (C), was derived and was connected with the pomalidomide as a ligand for cereblon for dual IGF-1R/Src degradation. We reasoned that a common chemical structure would target both IGF-1R and Src, bringing both proteins close to the E3 ligase for degradation in PROTACs. We also adopted previously reported 2,4-bis-arylamino-1,3-pyrimidine (D) as an IGF-1R warhead [41], and the aminopyrazolo [3,4-*d*]pyrimidine module (E) as a Src warhead for the PROTAC approach [42,43], and compared their efficacy to those of 12 with the newly designed inhibitor units. Through extensive optimization of the linker region, we have synthesized a series of dual IGF-1R/Src degraders. Among these, PROTACs 12a and 12b were capable of inducing the degradation of both IGF-1R and Src proteins at 1–5 µM in the MCF7 (human breast cancer) and A549 (human lung cancer) cells.

## 2. Results

### 2.1. Design and Synthesis

**Scheme 1:**
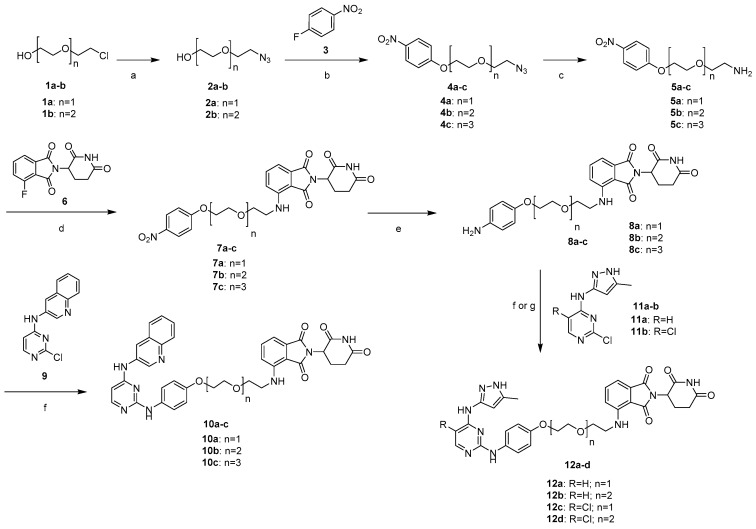
Reagents and conditions: (a) NaN_3_, H_2_O, 80 °C, 16 h, 69–82%; (b) K_2_CO_3_, DMSO, 80 °C, 4 h, 53–61%; (c) Ph_3_P, THF-H_2_O, RT, 14 h, 61–85%; (d) DIPEA, DMF, 90 °C, 16 h; (e) Fe (powder), NH_4_Cl, EtOH-H_2_O, 80 °C, 2 h (42–44% over 2 steps); (f) DMSO, 100 °C, 4 h (10a–c, 12a–b; 21–57%); (g) p-TsOH, butanol, 100 °C, 4 h (12c–d; 22–32%).

We developed a convergent synthetic route to prepare the designed dual IGF-1R/Src PROTAC degraders and their control compounds (Scheme 1). We prepared 4a–b in two steps. An azidation was first carried out from 2-chloropolyethoxy ethanols 1a-b to provide 2a–b [44], which were coupled with 4-flouronitrobenzene 3 to give 4a–b, respectively [45]. 4c was synthesized according to the previous reports, as depicted in the supplementary information [46]. The reduction of azides 4a–c, in the presence of triphenylphosphine, leads to the amine intermediates 5a–c, which were subsequently attached with thalidomide derivative 6 to obtain 7a–c, followed by a reduction with Fe/NH_4_Cl to provide 8a–c. PROTACs 10a–c were synthesized by coupling 8a–c with 2-chloro-4-arylamino-1,3-pyrimidines 9 in DMSO. The key intermediates 8a–b were also treated with their corresponding reagents 11a or 11b, to produce PROTACs 12a–d (Scheme 1) [36,47].

**Scheme 2:**
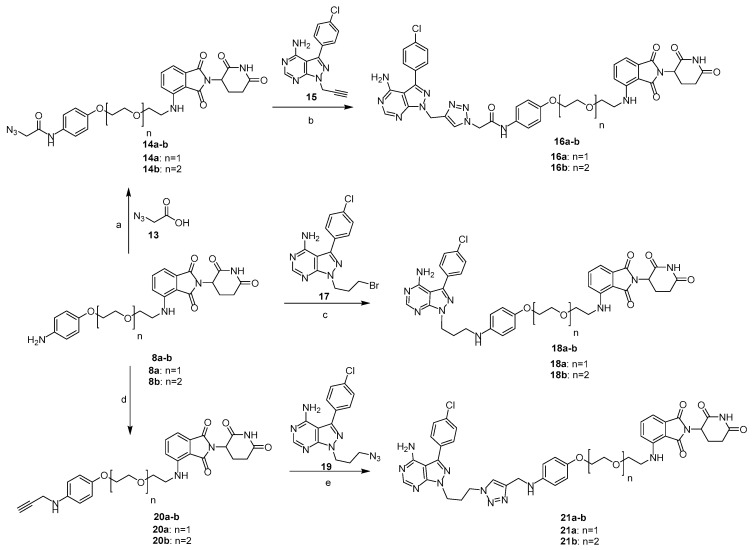
Reagents and conditions: (a) EDC·HCl, DIPEA, DMF, RT, 16 h, 40–62%; (b) CuSO_4_·5H_2_O, sodium ascorbate, THF, t-Butanol-H_2_O, RT, 6 h, 26–27%; (c) K_2_CO_3_, DMF, 16 h, 50.5–50.8%; (d) Propargyl bromide, K_2_CO_3_, DMF, 16 h, 52–55%; (e) CuSO_4_·5H_2_O, sodium ascorbate, THF, t-Butanol/H_2_O, RT, 6 h, 38–40%.

The thalidomide-attached linkers 8a–b were also coupled with azidoacetic acid 13 in the presence of EDC·HCl to provide 14a–b, which were further treated with *N*-substituted 4-aminopyrazolo[3–*d*]pyrimidines intermediate (15) in the presence of copper sulfate and sodium ascorbate in THF/H_2_O/tBuOH to obtain the click products 16a–b via a copper (I)-catalyzed alkyne azide 1,3-dipolar cycloaddition (CuAAC) reaction (Scheme 2). 8a-b reacted with the bromopropyl group-attached pyrazolo[3,4-*d*]pyrimidin-4-amine (17) under the basic condition yielded PROTACs 18a-b. Next, *N*-propagylation of 8a–b under the basic condition afforded 20a-b, which were coupled with azido intermediate (19) via the CuAAC reaction to obtain the desired triazole products 21a–b [36].

### 2.2. CPR3 (12a) and CPR4 (12b), Synthesized PROTAC Compounds, Inhibited Cancer Cell Proliferation

**Figure 2:**
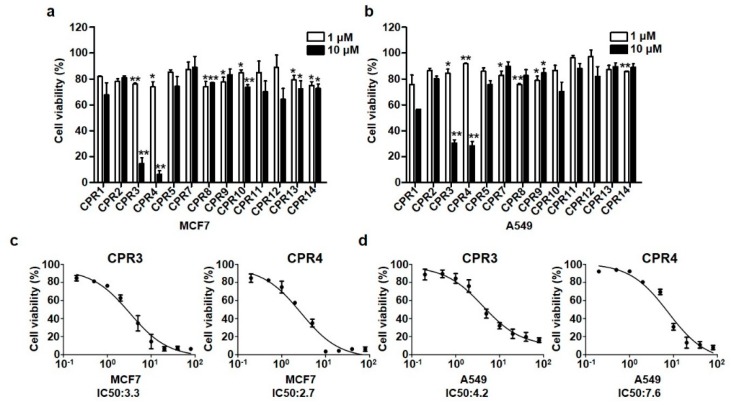
Inhibitory effect of PROTAC compounds on the viability of two different cancer cell lines, MCF7 (**a**) and A549 (**b**). PROTAC compounds were treated at 1 or 10 μM of concentration for 3 days in a complete RPMI-1640 medium. At various concentrations of CPR3 (**c**) or CPR4 (**d**), the IC_50_ value was calculated through the growth curve in both cell lines. Cell viability was determined by the changes in absorbance at 570 nm. * *P* ≤ 0.05 and ** *P* ≤ 0.01, as determined by Student’s t-test.

Next, we investigated the cell cytotoxicity of the synthesized PROTAC compounds 10a–c, 12a–d, 16a–d, 18a–b and 21a–b in both MCF7 (human breast cancer) and A549 (human lung cancer) cells. As the cell permeability is more crucial for PROTAC compounds with higher molecular weights than for conventional small-molecule inhibitors, we first screened the synthesized compounds via the MTT assay. Cell growth inhibition was measured at the concentrations of 1 or 10 μM PROTAC compounds, and the measured absorbance values were normalized to DMSO-treated cells. In Figure 2, CPR3 and CPR4 inhibited cell growth dose-dependently in both MCF7 (Figure 2a) and A549 (Figure 2b) cells. Compounds CPR3 and CPR4, with a methyl-1*H*-pyrazole group, showed significant growth inhibitions. In contrast, other compounds did not show significant dose-dependent growth inhibitions. IC_50_ values were further determined through the growth inhibition curve by varying the concentrations from 0.5 to 80 μM. The IC_50_ values of CPR3 and CPR4 were measured to be 3.3 and 2.7 μM in MCF7 cells (Figure 2c), whereas they were 4.2 and 7.6 μM in A549 cells (Figure 2d), respectively. Taken together, CPR3 and CPR4 showed anti-cancer potency by effectively inhibiting cancer cell growth.

### 2.3. CPR3 and CPR4 Degraded Both Src and IGF-1R Proteins

**Figure 3:**
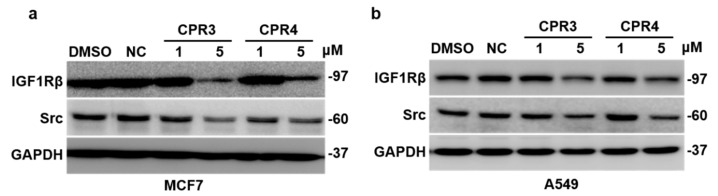
Immunoblotting for IGF-1R and Src in both MCF7 (**a**) and A549 (**b**) cells. The cells were treated with CPR3 or CPR4 at 1 and 5 μM of concentration for 24 h. NC was used as a negative control (5 μM). For detection of the protein expression, extracted lysate was incubated with IGF-1R or Src antibody for 1 h.

Cell growth inhibition is closely related to the post translational regulation of gene expression [48]. Therefore, we examined whether CPR3 and CPR4 reduce the expression level of the IGF-1R or Src proteins, serving as a dual degrader. 2-Chloro-*N*-(5-methyl-1*H*-pyrazol-3-yl)pyrimidin-4-amine (NC) was used as a negative control. In MCF7 cells, NC did not degrade Src or the IGF-1R protein, whereas CPR3 and CPR4 all degraded Src and IGF-1R at 5 μM of concentration (Figure 3a). Similarly, CPR3 and CPR4 also degraded Src and IGF-1R in A549 cells (Figure 3b). These results indicated that CPR3 and CPR4 play a key role as a dual degrader for both IGF-1R and Src proteins.

### 2.4. Invasion and Migration Ability Were Suppressed by CPR3 and CPR4 Treatment in Both MCF7 and A549 Cells

**Figure 4:**
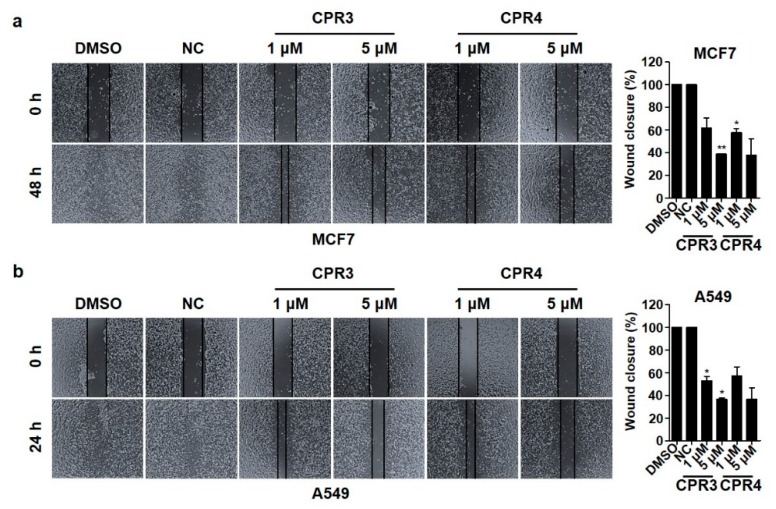
Wound healing images of MCF7 (**a**) and A549 (**b**) cells treated with CPR3 or CPR4 for 24 h. The PROTAC compound-treated cells were scratched with micropipette tip at 0 h. After 24 h, the migration status was monitored by bright optical microscopy. * *P* ≤ 0.05 and ** *P* ≤ 0.01, as determined by Student’s t-test.

**Figure 5:**
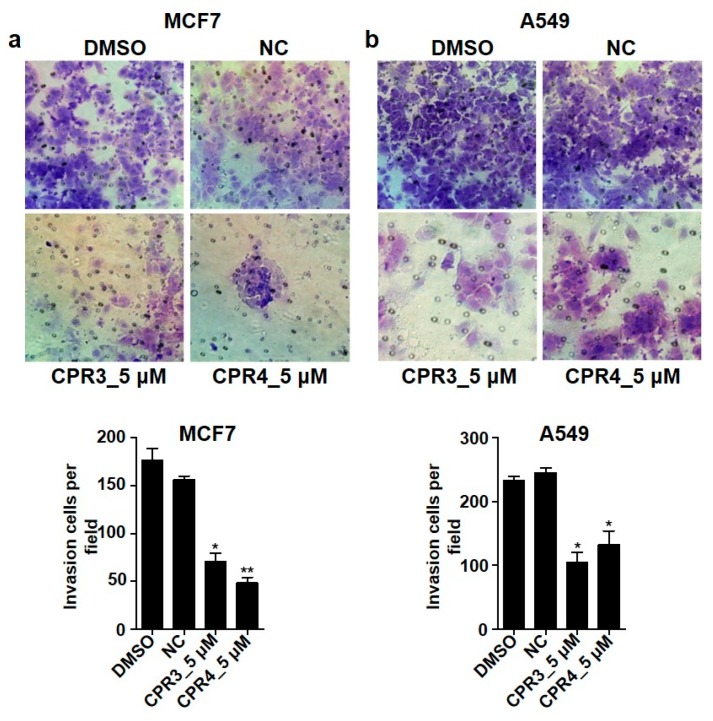
Invasiveness images of MCF7 (**a**) and A549 (**b**) cells treated with CPR3 or CPR4 for 24 h. The cells, resuspended in RPMI-1640 without serum, were reseeded on matrigel-coated insert transwell for 24 h. After 24 h, the cells that migrated to the membrane of the transwell were stained with 0.1% crystal violet and were counted with bright optical microscopy. * *P* ≤ 0.05 and ** *P* ≤ 0.01, as determined by Student’s t-test.

IGF-1R or Src mediated cancer cells were correlated with proliferation, differentiation, survival and metastasis, promoting cell-cell signal transduction. The characteristics of cancer cells have resistance to chemotherapy by processing the pathway related to the epithelial to mesenchymal transition (EMT) [49]. Therefore, we tested cell migration and invasiveness through the treatment of CPR3 and CPR4, which can block IGF-1R or Src in both MCF7 and A549 cells. In both cells, CPR3 and CPR4 significantly delayed the ratio of wound closure in a dose dependent manner, 1 or 5 μM (Figure 4), suggesting a reduction of migration by interrupting the pathway of IGF-1R or Src. In addition, Figure 4 shows a decreased invasiveness of CPR3 and CPR4 in both MCF7 (Figure 5a) and A549 (Figure 5b) cells. Cells with the ability to penetrate into the matrix were stained and counted on a bright field microscopy. The number of cells was significantly reduced at 5 μM of concentration of CPR3 and CPR4, as compared to DMSO (Figure 5). These results indicated that CPR3 and CPR4 can block the IGF-1R/Src regulated cancer cell progressions, including migration and invasion.

### 2.5. PROTAC Compounds Inhibited the Cell Growth of Both MCF7 and A549 Cells in the Soft Agar Colony Formation Assay

**Figure 6:**
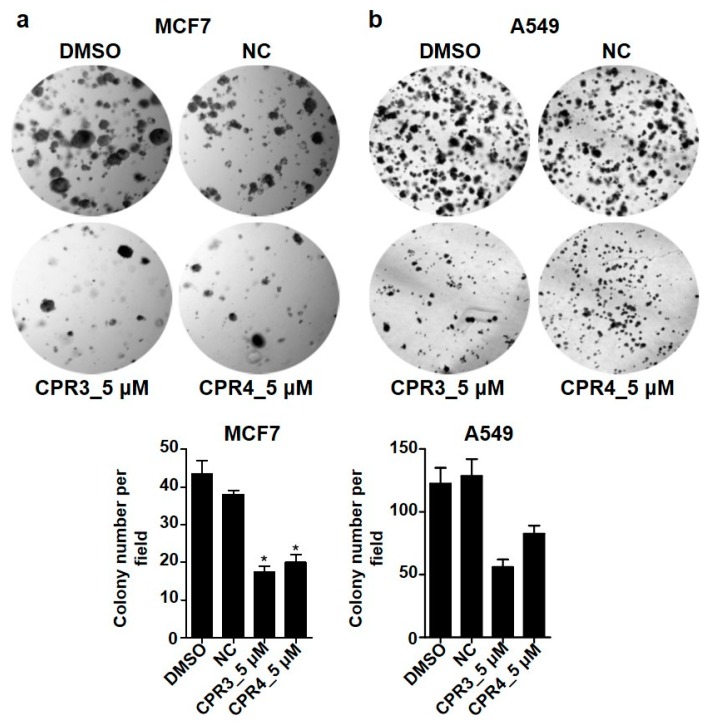
Soft agar colony formation images after treatment with CPR3 or CPR4 in both MCF7 (**a**) and A549 (**b**) cells. CPR3 or CPR4 was treated at 5 μM of concentration, followed by an incubation period of 2 weeks. The formed colonies were stained with 0.1% crystal violet and were detected on a bright field microscopy. * *P* ≤ 0.05, as determined by Student’s t-test.

Next, we examined tumorigenesis by treatment with PROTAC compounds in both MCF7 and A549 cells. It is well known that cancer cells differentiate rapidly and proliferate infinitely. In addition, the capability of single cells to form into a colony is a hallmark of cancer cell survival and proliferation. To test cellular anchorage-independent growth in vitro, we performed the soft agar colony formation assay after treatment with PROTAC compounds. In Figure 6, the number of colonies was significantly increased in DMSO or NC in both MCF7 (Figure 6a) and A549 (Figure 6b) cells. In contrast to the control group, the colony forming ability sharply declined with a 5 μM concentration of PROTAC compounds. Moreover, the sizes of the colonies formed from a single cell were much smaller in PROTAC compounds than in DMSO or NC. These results indicated that PROTAC compounds, with the dual degradation of IGF-1R and Src, affected cell survival.

## 3. Discussion

In this study, we rapidly synthesized and screened PROTACs for dual degradation of IGF-1R and Src by employing different warhead ligands and varied linker lengths and compositions, which brought target proteins and E3 ligases into proximity for ubiquitination. Our work demonstrated that efficient PROTAC molecules (12a–b), which had single warhead ligands that degraded two target proteins, exhibited low micromolar anticancer activity, measured by different cellular assays, including cancer cell proliferation, immunoblotting, wound healing assay, and soft agar colony formation assays.

Interestingly, the potency of the synthesized compounds obviously varied, depending on the warhead units. Our data revealed that the previously reported Src or IGF-1R modules (D and E) were not sufficient, as individual warheads, for dual PROTACs, whereas the *N*^2^-phenyl-*N^4^*-(1*H*-pyrazol-3-yl)pyrimidine-2,4-diamine (C) exhibited better potency for dual degradation. The induced degradation of the protein was found to be intrinsic to target proteins, which are affected by numerous factors, including the linker length and composition, E3 ligase ligand, spatial orientation of the formed ternary complex and their stability, cell permeability, and ubiquitination efficiency. One important factor for PROTACs is a drug-like property with cell permeability, for which atom numbers need to be minimized in the design of the chemical structure. Indeed, the linker size with 7-10 atoms, between the warhead ligand and the E3 ligase ligand in PROTACs 12a–b, appears to be sufficient for proximity-induced ubiquitination of Src or IGF-1R with the E3 ligase.

The possibility of off-target degradation is still likely to happen with small-warhead ligands, due to their promiscuity. However, recent advancements in PROTACs with promiscuous kinase inhibitors have shown that efficiency is not directly correlated with the binding affinity of the ligands [27]. Rather, it is more closely related to the geometry of the ternary complex of the target protein-PROTAC-E3 ligases, as well as to the efficiency of the proximity-induced enzymatic ubiquitin transfer. Further optimization needs to be followed in regard to the optimal CRBN ligand orientation, nano to pico molar potency, etc. However, our work demonstrated a facile preparation of efficient dual degraders, which enrich our knowledge in establishing dual degradation as an alternative therapeutic strategy in cancers.

## 4. Materials and Methods

### 4.1. Chemistry

#### 4.1.1. General Information

All reagents and solvents were obtained from commercial suppliers and used as received unless otherwise specified. All reactions were performed under nitrogen atmosphere using oven-dried glassware and monitored by thin-layer chromatography (TLC) on a silica-coated plate (60 F254, Merck, Darmstadt, Germany). Separated compounds on the TLC plate were visualized under UV light at 254 nm and 365 nm (VL-4.LC, Vilber Lourmat, Eberhardzell, Germany). Column chromatography was carried out using a silica gel 230-400 mesh (ZEOprep, Zeochem, Lake Zurich, Switzerland) with *n*-hexane, EtOAc, CH_2_Cl_2_ and MeOH as eluents. ^1^H NMR (400 MHz) and ^13^C NMR (125 MHz) spectra were recorded using a FT-NMR Avance III HD (Bruker, Billerica, MA, USA) at ambient temperature. Chemical shifts were reported in ppm (parts per million) relative to tetramethylsilane and coupling constants (*J*) were expressed in hertz (Hz). The following abbreviations are used for multiplicities: s = singlet; brs = broad singlet; d = doublet; t = triplet; q = quartet; m = multiplet; dd = doublet of doublets. Mass-to-charge ratio (m/z) values were obtained by using high-resolution mass spectrometry (HRMS) under fast atom bombardment (FAB) conditions with a JMS-700 MStation (JEOL, Tokyo, Japan).

#### 4.1.2. Synthesis of 10a–c, 12a–d, 16a–b, 18a–b and 21a–b

General procedure for synthesis of 8a–c: A reaction mixture of 7a–c (11.036 mmol, 1.0 eq), iron powder (10.363 mmol, 10 eq) and ammonium chloride (10.363 mmol, 10 eq) in ethanol-water (8:2) was stirred for 2 h under N_2_ atmosphere at 80 °C. The reaction mixture was cooled down to room temperature, filtered through celite bed, which was washed twice with ethyl acetate. Solvent was removed under reduced pressure and the obtained residue was dissolved in ethyl acetate and washed with water and brine solution. Organic layer was dried over anhydrous sodium sulfate. Upon concentration under reduced pressure, the residue was purified by column chromatography on silica gel to give 8a–c in 42–44% yield.

*4-((2-(2-(4-aminophenoxy)ethoxy)ethyl)amino)-2-(2,6-dioxopiperidin-3-yl)isoindoline-1,3-dione (**8a**):* Yellow solid; yield 42.1%; *R*_f_ = 0.50 (methanol/dichloromethane = 0.5:9.5); ^1^H-NMR (400 MHz, CDCl_3_) δ 8.57 (s, 1H), 7.43 (t, *J* = 8.8 Hz, 1H), 7.06 (d, *J* = 7.2 Hz, 1H), 6.89 (d, *J* = 8.4 Hz, 1H), 6.71 (d, *J* = 9.2 Hz, 2H), 6.58 (d, *J* = 9.2 Hz, 2H), 6.49 (t, *J* = 5.6 Hz, 1H), 4.87 (dd, *J* = 5.6, 12.0 Hz, 1H), 4.03 (t, *J* = 4.8 Hz, 2H), 3.80–3.74 (m, 4H), 3.45 (dd, *J* = 5.6, 11.2 Hz, 2H), 2.84–2.65 (m, 3H), 2.08–2.02 (m, 1H); ^13^C-NMR (125 MHz, CDCl_3_) δ 171.39, 169.18, 168.53, 167.59, 151.80, 146.77, 140.14, 135.95, 132.42, 116.75, 116.31 (2C), 115.87 (2C), 111.57, 110.24, 69.89, 69.63, 68.21, 48.78, 42.36, 31.32, 22.67; HR-MS (FAB^+^) calcd for C_23_H_25_N_4_0_6_ [M + H]^+^ 453.1774, found 453.1777.

*4-((2-(2-(2-(4-aminophenoxy)ethoxy)ethoxy)ethyl)amino)-2-(2,6-dioxopiperidin-3-yl)isoindoli ne-1,3-dione (**8b**)*: Yellow solid; yield 44.7%; *R*_f_ = 0.50 (methanol/dichloromethane = 1:9); ^1^H-NMR (400 MHz, CDCl_3_) δ 8.54 (s, 1H), 7.44 (t, *J* = 7.2 Hz, 1H), 7.05 (d, *J* = 7.2 Hz, 1H), 6.88 (d, *J* = 8.4 Hz, 1H), 6.71 (d, *J* = 8.8 Hz, 2H), 6.58 (d, *J* = 8.8 Hz, 2H), 6.47 (t, *J* = 5.6 Hz, 1H), 4.84 (dd, *J* = 5.2, 12.0 Hz, 1H), 4.02 (t, *J* = 4.8 Hz, 2H), 3.79 (t, *J* = 5.2 Hz, 2H), 3.71–3.65 (m, 6H), 3.43 (dd, *J* = 5.6, 11.2 Hz, 3H), 2.77–2.64 (m, 3H), 2.04-2.00 (m, 1H); ^13^C-NMR (125 MHz, CDCl_3_) δ 171.29, 169.18, 168.46, 167.59, 151.85, 146.77, 140.11, 135.95, 132.42, 116.73, 116.30 (2C), 115.79 (2C), 111.53, 110.19, 70.70, 70.66, 69.92, 69.44, 68.07, 48.78, 42.32, 31.31, 22.65; HR-MS (FAB^+^) calcd for C_25_H_29_N_4_0_7_ [M + H]^+^ 497.2036, found 497.2029.

*4-((2-(2-(2-(2-(4-aminophenoxy)ethoxy)ethoxy)ethoxy)ethyl)amino)-2-(2,6-dioxopiperidin-3-yl)isoindoline-1,3-dione (**8c**):* Yellow solid; yield 42.9%; *R*_f_ = 0.50 (methanol/dichloromethane = 1:9); ^1^H-NMR (400 MHz, CDCl_3_) δ 8.46 (s, 1H), 8.13 (d, *J* = 9.2 Hz, 2H), 7.44 (t, *J* = 8.4 Hz, 1H), 7.05 (d, *J* = 6.8 Hz, 1H), 6.93 (d, *J* = 9.2 Hz, 2H), 6.87 (d, *J* = 8.4 Hz, 1H), 6.44 (t, *J* = 5.6 Hz, 1H), 4.88 (dd, *J* = 6.0, 12.4 Hz, 1H), 4.17 (t, *J* = 4.4 Hz, 2H), 3.85 (t, *J* = 4.8 Hz, 2H), 3.70–3.64 (m, 10H), 3.42 (dd, *J* = 5.6, 11.1 Hz, 2H), 2.85–2.68 (m, 3H), 2.10–2.02 (m, 1H); ^13^C-NMR (125 MHz, CDCl_3_) δ 171.36, 169.18, 168.54, 167.49, 163.75, 146.68, 141.42, 135.92, 132.35, 125.73 (2C), 116.67, 114.48 (2C), 111.50, 110.10, 70.75, 70.54, 70.50, 69.40, 69.22, 68.10, 48.75, 42.26, 31.28, 22.63; HR-MS (FAB^+^) calcd for C_27_H_33_N_4_O_8_ [M + H]^+^ 541.2298, found 541.2299.

General procedure for synthesis of 10a–c and 12a–b: A reaction mixture of 8a–c (0.044 mmol, 1.0 eq) and corresponding pyrimidines 9 or 11a–b (0.044 mmol, 1.0 eq) were dissolved in DMSO (1.0 mL), and the resulting mixture was stirred for 16 h under N_2_ atmosphere at 90 °C. The progress of the reaction was monitored by TLC. After completion of the reaction, 50 mL of cold water was added and extracted with ethyl acetate (3 × 50 mL). The combined organic layer was dried over anhydrous sodium sulfate. Upon concentration under reduced pressure, the residue was purified by column chromatography on silica gel to give 10a–c and 12a–b, respectively. 21–57% yields.

*2-(2,6-dioxopiperidin-3-yl)-4-((2-(2-(4-((4-(quinoline-3-ylamino)pyrimidin-2-yl)amino)pheno xy)ethoxy)ethyl)amino)isoindoline-1,3-dione (**10a**, CPR-2)*: Yellow solid; yield 52.0%; *R*_f_ = 0.45 (methanol/dichloromethane = 1:9); mp 160.2–161.8 °C; ^1^H-NMR (400 MHz, MeOD + CDCl_3_) δ 8.81 (s, 1H), 8.70 (s, 1H), 7.90 (d, *J* = 5.6 Hz, 1H), 7.85–7.83 (m, 1H), 7.53–7.42 (m, 4H), 7.35 (d, *J* = 9.2 Hz, 2H), 6.98-6.95 (m, 2H), 6.87 (d, *J* = 9.2 Hz, 2H), 6.19 (d, *J* = 6.0 Hz, 1H), 4.85 (dd, *J* = 5.6, 12.4 Hz, 1H), 4.14 (t, *J* = 4.4 Hz, 2H), 3.87 (t, *J* = 4.8 Hz, 2H), 3.79 (t, *J* = 5.6 Hz, 2H), 3.48 (t, *J* = 5.2 Hz, 2H), 2.68–2.53 (m, 3H), 1.99–1.87 (m, 1H); ^13^C-NMR (125 MHz, MeOD + CDCl_3_) δ 174.08, 170.92, 170.46, 169.29, 162.30, 161.34, 156.69, 156.32, 147.99, 145.73, 144.42, 137.28, 134.97, 133.92, 133.60, 129.87, 128.86, 128.83 (2C), 128.26, 124.99, 124.87, 118.17, 116.25 (2C), 112,67, 111.33, 99.83, 71.05, 70.95, 69.10, 50.06, 43.45, 32.49, 23.85; HRMS (FAB^+^) calcd for C_36_H_33_N_8_O_6_ [M + H]^+^ 673.2523, found 673.2532.

*2-(2,6-dioxopiperidin-3-yl)-4-((2-(2-(2-(4-((4-(quinolin-3-ylamino)pyrimidin-2-yl)amino)phen oxy)ethoxy)ethoxy)ethyl)amino)isoindoline-1,3-dione (**10b**, CPR-5)*: Yellow solid; yield 57.7%; *R*_f_ = 0.45 (methanol/dichloromethane = 1:9); mp 185.3–186.4 °C; ^1^H-NMR (400 MHz, DMSO-d_6_) δ 11.06 (s, 1H), 9.82 (s, 1H), 9.10 (s, 1H), 8.85 (s, 2H), 8.02 (d, *J* = 5.6 Hz, 1H), 7.86 (d, *J* = 9.2 Hz, 1H), 7.69 (brs, 1H), 7.54–7.49 (m, 5H), 7.08 (d, *J* = 8.8 Hz, 1H), 6.97 (d, *J* = 6.8 Hz, 1H), 6.83 (d, *J* = 9.2 Hz, 2H), 6.57 (t, *J* = 6.0 Hz, 1H), 6.25 (d, *J* = 5.6 Hz, 1H), 4.99 (dd, *J* = 5.6, 12.8 Hz, 1H), 4.01 (t, *J* = 4.4 Hz, 2H), 3.72 (t, *J* = 4.8 Hz, 2H), 3.60–3.56 (m, 6H), 3.46–3.40 (m, 2H), 2.85–2.76 (m, 1H), 2.53–2.48 (m, 2H), 1.96–1.91 (m, 1H); ^13^C-NMR (125 MHz, MeOD + CDCl_3_) δ 173.69, 170.58, 170.29, 169.04, 162.05, 161.17, 156.78, 156.05, 147.74, 145.64, 144.31, 137.07, 134.69, 133.73, 133.39, 129.66, 128.84, 128.64, 128.61, 128.09, 124.68, 124.61 (2C), 117.91, 115.97 (2C), 112.52, 111.07, 99.53, 71.82, 71.67, 70.91, 70.53, 68.82, 49.88, 43.21. 32.35, 23.68; HRMS (FAB^+^) calcd for C_38_H_37_N_8_O_7_ [M + H]^+^ 717.2785, found 717.2770.

*2-(2,6-dioxopiperidin-3-yl)-4-((2-(2-(2-(2-(4-((4-(quinolin-3-ylamino)pyrimidin-2-yl)amino) phenoxy)ethoxy)ethoxy)ethoxy)ethyl)amino)isoindoline-1,3-dione (**10c**, CPR-1)*: Yellow solid; yield 21.0%; *R*_f_ = 0.45 (methanol/dichloromethane = 1:9); mp 151.8–152.9 °C; ^1^H-NMR (400 MHz, DMSO-d_6_) δ 11.05 (s, 1H), 9.77 (s, 1H), 9.05 (s, 1H), 8.88 (brs, 1H), 8.86 (s, 1H), 8.03 (d, *J* = 5.6 Hz, 1H), 7.87 (d, *J* = 9.6 Hz, 1H), 7.70 (brs, 1H), 7.55–7.48 (m, 5H), 7.07 (d, *J* = 8.4 Hz, 1H), 6.97 (d, *J* = 6.8 Hz, 1H), 6.84 (d, *J* = 9.2 Hz, 2H), 6.55 (t, *J* = 5.6 Hz, 1H), 6.25 (d, *J* = 5.6 Hz, 1H), 5.01 (dd, *J* = 5.0, 12.8 Hz, 1H), 4.01 (t, *J* = 4.4 Hz, 2H), 3.70 (t, *J* = 4.4 Hz, 2H), 3.58–3.52 (m, 10H), 3.40 (dd, *J* = 5.2, 10.8 Hz, 2H), 2.87–2.78 (m, 1H), 2.55–2.49 (m, 2H), 1.99–1.94 (m, 1H); ^13^C-NMR (125 MHz, CDCl_3_) δ 172.76, 170.03, 169.22, 167.68, 160.78, 159.85, 156.15, 154.50, 146.63, 145.50, 144.23, 135.89, 132.88, 132.86, 132.32, 128.72, 128.26, 127.63, 127.36, 126.94, 123.82, 122.48 (2C), 116.67, 114.82 (2C), 111.51, 110.08, 97.51, 70.78, 70.68, 70.60 (2C), 69.74, 69.34, 67.72, 48.83, 42.28, 31.44, 22.79; HR-MS (FAB^+^) calcd for C_40_H_41_N_8_O_8_ [M + H]^+^ 761.3047, found 761.3039.

*2-(2,6-dioxopiperidin-3-yl)-4-((2-(2-(4-((4-((5-methyl-1H-pyrazol-3-yl)amino)pyrimidin-2-yl) amino)phenoxy)ethoxy)ethyl)amino)isoindoline-1,3-dione (**12a**, CPR-3)*: Yellow solid; yield 32.6%; *R*_f_ = 0.45 (methanol/dichloromethane = 1:9); mp 158.1–159.3 °C; ^1^H-NMR (400 MHz, MeOD + CDCl_3_) δ 7.84 (d, *J* = 6.0 Hz, 1H), 7.42 (dd, *J* = 7.2, 8.4 Hz, 1H), 7.34 (d, *J* = 9.2 Hz, 2H), 7.00 (d, *J* = 6.8 Hz, 1H), 6.90 (d, *J* = 8.8 Hz, 1H), 6.83 (d, *J* = 8.8 Hz, 2H), 6.13 (s, 1H), 5.87 (s, 1H), 4.85 (dd, *J* = 5.6, 12.4 Hz, 1H), 4.08 (t, *J* = 4.4 Hz, 2H), 3.80 (t, *J* = 4.8 Hz, 2H), 3.73 (t, *J* = 5.2 Hz, 2H), 3.44 (t, *J* = 5.2 Hz, 2H), 2.77–2.58 (m, 3H), 2.16 (s, 3H), 2.04–1.99 (m, 1H); ^13^C-NMR (125 MHz, DMSO-d_6_) δ 172.84, 170.11, 168.94, 167.31, 160.75, 159.39, 157.14, 150.01, 147.37, 146.40, 141.86, 138.84, 136.21, 132.11, 117.41, 115.46 (2C), 115.24 (2C), 110.70, 109.31, 104.99, 95.52, 69.09, 68.93, 67.64, 48.58, 41.71, 31.01, 22.16, 10.63; HR-MS (FAB^+^) calcd for C_31_H_32_N_9_O_6_ [M + H]^+^ 626.2476, found 626.2477.

*2-(2,6-dioxopiperidin-3-yl)-4-((2-(2-(2-(4-((4-((5-methyl-1H-pyrazol-3-yl)amino)pyrimidin-2-yl)amino)phenoxy)ethoxy)ethoxy)ethyl)amino)isoindoline-1,3-dione (**12b**, CPR-4)*: Yellow solid; yield 44.5%; *R*_f_ = 0.45 (methanol/dichloromethane = 1:9); mp 176.2–177.5 °C; ^1^H-NMR (400 MHz, MeOD + CDCl_3_) δ 7.75 (d, *J* = 6.4 Hz, 1H), 7.43 (dd, *J* = 7.2, 8.4 Hz, 1H), 7.32 (d, *J* = 8.8 Hz, 2H), 7.00 (d, *J* = 7.2 Hz, 1H), 6.91 (d, *J* = 8.8 Hz, 1H), 6.86 (d, *J* = 9.2 Hz, 2H), 6.23 (s, 1H), 5.96 (s, 1H), 4.85 (dd, *J* = 5.2, 11.2 Hz, 1H), 4.08 (t, *J* = 4.4 Hz, 2H), 3.83 (t, *J* = 4.8 Hz, 2H), 3.71–3.65 (m, 6H), 3.42 (t, *J* = 5.2 Hz, 2H), 2.75–2.65 (m, 3H), 2.18 (s, 3H), 2.06–1.98 (m, 1H); ^13^C-NMR (125 MHz, DMSO-d_6_) δ 172.80, 170.08, 168.94, 167.29, 160.72, 159.36, 157.06, 150.84, 147.35, 146.40, 140.13, 138.85, 136.20, 132.08, 117.44, 116.17 (2C), 115.31 (2C), 110.66, 109.24, 105.01, 95.47, 69.90, 69.79, 69.19, 68.91, 67.53, 48.55, 41.68, 30.98, 22.14, 10.62; HRMS (FAB^+^) calcd for C_33_H_36_N_9_O_7_ [M + H]^+^ 670.2738, found 670.2745.

*4-((2-(2-(4-((5-chloro-4-((5-methyl-1H-pyrazol-3-yl)amino)pyrimidin-2-yl)amino)phenoxy) ethoxy)ethyl)amino)-2-(2,6-dioxopiperidin-3-yl)isoindoline-1,3-dion(**12c**, CPR-10)*: To the solution of 8a (30 mg, 0.066 mmol) in n-butanol (4.0 mL) were added 11b (16 mg, 0.066 mmol) and p-toluenesulfonic acid monohydrate (13.5 mg, 0.066 mmol). The resulting mixture was stirred for 4 h under N_2_ atmosphere at 100 °C. The progress of reaction was monitored by TLC. After completion of the reaction, the reaction mixture was cooled down to room temperature, saturated NaHCO_3_ solution was added to adjust pH to 7, then extracted with ethyl acetate (3 x 20 mL). The combined organic layer was dried over anhydrous sodium sulfate. After concentration of the reaction mixture under reduced pressure, the residue was purified by column chromatography on silica gel to give 12c (13.19 mg, 32.5%) as a yellow solid. *R*_f_ = 0.45 (methanol/dichloromethane = 1:9); mp 196.2–197.8 °C; ^1^H-NMR (400 MHz, MeOD + CDCl_3_) δ 7.90 (s, 1H), 7.46 (t, *J* = 8.4 Hz, 1H), 7.32 (d, *J* = 8.8 Hz, 2H), 6.98 (dd, *J* = 7.2, 10.4 Hz, 2H), 6.85 (d, *J* = 8.8 Hz, 2H), 6.20 (s, 1H), 4.92 (dd, *J* = 6.0, 12.4 Hz, 1H), 4.11 (t, *J* = 4.4 Hz, 2H), 3.83 (t, *J* = 4.4 Hz, 2H), 3.76 (t, *J* = 5.2 Hz, 2H), 3.47 (t, *J* = 5.2 Hz, 2H), 2.74–2.63 (m, 3H), 2.20 (s, 3H), 2.04–1.96 (m, 1H); ^13^C-NMR (125 MHz, DMSO-d_6_) δ 172.80, 170.07, 168.91, 167.28, 157.04, 156.78, 155.09, 149.72, 146.38, 145.70, 142.45, 138.74, 136.19, 132.09, 117.40, 115.44 (2C), 114.91 (2C), 113.15, 110.67, 109.28, 98.02, 69.08, 68.92, 67.63, 48.56, 41.70, 30.98, 22.14, 10.73; HR-MS (FAB^+^) calcd for C_31_H_31_ClN_9_O_6_ [M + H]^+^ 660.2086, found 660.2077.

*4-((2-(2-(2-(4-((5-chloro-4-((5-methyl-1H-pyrazol-3-yl)amino)pyrimidin-2-yl)amino)phenoxy) ethoxy)ethoxy)ethyl)amino)-2-(2,6-dioxopiperidin-3-yl)isoindoline-1,3-dione (**12d**, CPR-12)*: 12d was synthesized according to the procedure for 12c. Yellow solid; yield 22.6%; *R*_f_ = 0.55 (EtOAc); mp 180.9–182.1 °C; ^1^H-NMR (400 MHz, CDCl_3_) δ 8.00 (brs, 1H), 7.94 (s, 1H), 7.60 (brs, 1H), 7.42 (t, *J* = 7.2 Hz, 1H), 7.30 (d, *J* = 9.2 Hz, 2H), 7.01 (d, *J* = 7.2 Hz, 1H), 6.87 (d, *J* = 8.4 Hz, 1H), 6.78–6.71 (m, 2H), 6.63 (brs, 1H), 6.42 (t, *J* = 5.6 Hz, 1H), 6.03 (s, 1H), 4.85 (dd, *J* = 5.6, 12.4 Hz, 1H), 4.11–4.08 (m, 2H), 3.84 (t, *J* = 4.8 Hz, 2H), 3.75–3.69 (m, 6H), 3.46–3.42 (m, 2H), 2.81–2.59 (m, 3H), 2.24 (s, 3H), 2.05–2.02 (m, 1H); ^13^C-NMR (125 MHz, DMSO-d_6_) δ 172.79, 170.07, 168.93, 167.28, 157.04, 156.78, 155.10, 149.74, 146.40, 145.69, 142.41, 138.74, 136.19, 132.08, 117.42, 115.30 (2C), 114.92 (2C), 113.16, 110.65, 109.24, 98.01, 69.90, 69.79, 69.24, 68.91, 67.54, 48.56, 41.69, 30.98, 22.14, 10.73; HR-MS (FAB^+^) calcd for C_33_H_35_ClN_9_O_7_ [M + H]^+^ 704.2348, found 704.2340.

General procedure for synthesis of 14a–b: Dried round bottom flask with stir bar was charged with azidoacetic acid 13 (0.06 mmol, 1.0 eq) and *N*-ethyl-*N*′-(3-dimethylaminopropyl)carbodiimide hydrochloride (EDC·HCl) (0.120 mmol, 2.0 eq) followed by slow addition of dry DMF (3 mL). Then substituted alkyl amine 8a–b (0.06 mmol, 1.0 eq) was added, followed by addition of diisopropyl ethylamine (0.018 mmol, 3.0 eq). The resulting mixture was stirred for 16 h at room temperature. After completion of the reaction, cooled water was added to the reaction mixture, which was extracted with ethyl acetate twice. Separated organic layer was dried with anhydrous sodium sulfate and solvent was evaporated under reduced pressure. Crude product was purified by column chromatography on silica gel using EtOAc: Hexane as mobile phase to get desired product 14a–b in 40–63% yields.

*2-azido-N-(4-(2-(2-((2-(2,6-dioxopiperidin-3-yl)-1,3-dioxoisoindolin-4-yl)amino)ethoxy)ethox y)phenyl)acetamide (**14a**):* Yellow solid; yield 40.6%; *R*_f_ = 0.50 (methanol/dichloromethane = 1:9); ^1^H-NMR (400 MHz, CDCl_3_) δ 8.23 (s, 1H), 7.97 (s, 1H), 7.46 (t, *J* = 7.2 Hz, 1H), 7.38 (d, *J* = 9.2 Hz, 2H), 7.07 (d, *J* = 7.2 Hz, 1H), 6.88 (t, *J* = 8.8 Hz, 3H), 6.53 (brs, 1H), 4.88 (dd, *J* = 5.2 Hz, *J* = 12.0 Hz 1H), 4.14-4.10 (m, 4H), 3.84 (t, *J* = 4.8 Hz, 2H), 3.78 (t, *J* = 5.2 Hz, 2H), 3.46 (t, *J* = 5.2 Hz, 2H), 2.87–2.70 (m, 3H), 2.11–2.08 (m, 1H); ^13^C-NMR (125 MHz, CDCl_3_) δ 171.08, 169.17, 168.41, 167.59, 164.47, 156.01, 146.79, 136.02, 132.46, 130.08, 121.77 (2C), 116.74, 115.19 (2C), 111.69, 110.33, 69.80, 69.72, 67.88, 52.96, 48.84, 42.41, 31.40, 22.76; HR-MS (FAB^+^) calcd for C_25_H_26_N_7_O_7_ [M + H]^+^ 536.1894, found 536.1894.

*2-azido-N-(4-(2-(2-(2-((2-(2,6-dioxopiperidin-3-yl)-1,3-dioxoisoindolin-4-yl)amino)ethoxy) ethoxy)ethoxy)phenyl)acetamide (**14b**)*: Yellow solid; yield 62.8%; *R*_f_ = 0.70 (ethyl acetate); ^1^H-NMR (400 MHz, CDCl_3_) δ 8.08 (s, 1H), 7.93 (s, 1H), 7.46 (t, *J* = 8.0 Hz, 1H), 7.38 (d, *J* = 8.8 Hz, 2H), 7.07 (d, *J* = 7.2 Hz, 1H), 6.90–6.84 (m, 3H), 6.46 (t, *J* = 6.4 Hz, 1H), 4.83 (dd, *J* = 6.8, 13.2 Hz, 1H), 4.12-4.07 (m, 4H), 3.84 (t, *J* = 4.8 Hz, 2H), 3.73–3.68 (m, 6H), 3.44 (dd, *J* = 5.6 Hz, 2H), 2.84–2.65 (m, 3H), 2.08–2.03 (m, 1H); ^13^C-NMR (125 MHz, CDCl_3_) δ 170.93, 169.20, 168.28, 167.60, 164.42, 156.09, 146.82, 136.04, 132.49, 129.99, 121.83 (2C), 116.76, 115.01 (2C), 111.63, 110.24, 70.86, 70.76, 69.76, 69.49, 67.71, 52.96, 48.84, 42.41, 31.39, 22.71; HR-MS (FAB^+^) calcd for C_27_H_30_N_7_O_8_ [M + H]^+^ 580.2156, found 580.2156.

*3-(4-chlorophenyl)-1-(prop-2-yn-1-yl)-1H-pyrazolo[3,4-d]pyrimidin-4-amine* (**15**): Compound 15 was synthesized according to the reported procedure [36,50].

*2-(4-((4-amino-3-(4-chlorophenyl)-1H-pyrazol[3,4-d]pyrimidin-1-yl)methyl)-1H-1,2,3-triazol -1-yl)-N-(4-(2-(2-((2-(2,6-dioxopiperidin-3-yl)-1,3-dioxoisoindolin-4-yl)amino)ethoxy)ethoxy)phen yl)acetamide (**16a**, CPR-7)*: To a reaction mixture of 8a (12 mg, 0.022 mmol) and 15 (6.3 mg, 0.022 mmol) dissolved in THF (1.0 mL) was added t-butanol (0.5 mL). Then CuSO_4_·5H_2_O (1.1 mg, 0.004 mmol) dissolved in 0.2 mL of water was added to it, followed by the addition of sodium ascorbate (3.55 mg, 0.017 mmol) dissolved in 0.2 mL of water. The reaction mixture was stirred at room temperature for 6 h. The progress of reaction was monitored by TLC. After completion of the reaction, solvent was evaporated, and the residue was extracted with ethyl acetate (3 x 20 mL). The combined organic layer was dried over anhydrous sodium sulfate. Upon concentration under reduced pressure, the residue was purified by column chromatography on silica gel to give 16a (5 mg, 27.2%) as a yellow solid. *R*_f_ = 0.15 (methanol/ethyl acetate = 1:9); mp 186.3–187.6 °C; ^1^H-NMR (400 MHz, MeOD + CDCl_3_) δ 8.28 (s, 1H), 7.98 (s, 1H), 7.59 (d, *J* = 8.4 Hz, 2H), 7.48-7.41 (m, 3H), 7.32 (d, *J* = 9.2 Hz, 2H), 6.99 (d, *J* = 6.8 Hz, 1H), 6.93 (d, *J* = 8.4 Hz, 1H), 6.79 (d, *J* = 9.2 Hz, 2H), 5.71 (s, 2H), 5.15 (s, 2H), 4.87 (dd, *J* = 6.0, 12.4 Hz, 1H), 4.07 (t, *J* = 4.4 Hz, 2H), 3.80 (t, *J* = 4.8 Hz, 2H), 3.74 (t, *J* = 5.2 Hz, 2H), 3.44 (t, *J* = 5.2 Hz, 2H), 2.73–2.63 (m, 3H), 2.05–1.96 (m, 1H); ^13^C-NMR (125 MHz, MeOD + CDCl_3_) δ 173.62, 170.49, 170.22, 169.03, 164.18, 157.27, 156.78, 154.71, 147.73, 145.77, 143.70, 137.07, 136.73, 133.37, 131.74 (2C), 131.65 (2C), 130.76, 130.61, 126.08, 124.26, 122.55 (2C), 117.90, 115.99 (2C), 112.55, 112.16, 111.12, 70.73, 70.68, 68.78, 53.81, 43.45, 43.22, 32.33, 30.61, 23.68; HR-MS (FAB^+^) calcd for C_39_H_36_ClN_12_O_7_ [M + H]^+^ 819.2518, found 819.2532.

*2-(4-((4-amino-3-(4-chlorophenyl)-1H-pyrazolo[3,4-d]pyrimidin-1-yl)methyl)-1H-1,2,3-triazol-1-yl)-N-(4-(2-(2-(2-((2-(2,6-dioxopiperidin-3-yl)-1,3-dioxoisoindolin-4-yl)amino)ethoxy) ethoxy)ethoxy)phenyl)acetamide (**16b**, CPR-9)*: 16b was synthesized according to the procedure for 16a. Yellow solid; yield 26.8%; *R*_f_ = 0.20 (ethyl acetate); mp 192.8–194.1 °C; ^1^H-NMR (400 MHz, MeOD) δ 8.28 (s, 1H), 7.92 (s, 1H), 7.55 (d, *J* = 6.4 Hz, 2H), 7.46-7.38 (m, 4H), 7.31 (d, *J* = 9.2 Hz, 2H), 6.99 (d, *J* = 6.8 Hz, 1H), 6.86 (d, *J* = 8.4 Hz, 1H), 6.76 (d, *J* = 9.2 Hz, 2H), 5.69 (s, 2H), 5.09 (s, 2H), 4.77 (dd, *J* = 5.2, 12.4 Hz, 1H), 3.78 (t, *J* = 4.8 Hz, 2H), 3.68-3.58 (m, 6H), 3.39 (t, *J* = 5.2 Hz, 2H), 3.29-3.27 (m, 2H), 2.73-2.59 (m, 3H), 2.00–1.94 (m, 1H); ^13^C-NMR (125 MHz, MeOD + CDCl_3_) δ 173.69, 170.51, 170.29, 169.19, 169.12, 164.28, 157.73, 156.85, 147.78, 137.14, 136.81, 136.43, 133.42, 131.81 (2C), 131.64 (2C), 130.82, 130.67, 129.34, 126.22, 122.61 (2C), 119.64, 117.97, 117.61, 115.87 (2C), 112.53, 111.08, 71.75, 71.65, 70.79, 70.49, 68.66, 53.80, 43.50, 43.23, 32.36, 30.67, 23.70; HR-MS (FAB^+^) calcd for C_41_H_40_ClN_12_O_8_ [M + H]^+^ 863.2781, found 863.2768.

*1-(3-bromopropyl)-3-(4-chlorophenyl)-1H-pyrazolo[3,4-d]pyrimidin-4-amine* (**17**): To a reaction mixture of 22 (400 mg, 1.628 mmol) and 1,3-dibromo propane (394 mg, 1.953 mmol) dissolved in DMF (4.0 mL) was added K_2_CO_3_ (562 mg, 4.070 mmol). The resulting mixture was stirred at room temperature for 16 h. The progress of the reaction was monitored by TLC. After completion of the reaction, cold water was added and extracted with ethyl acetate (3 x 20 mL). The combined organic layer was dried over anhydrous sodium sulfate. Upon concentration under reduced pressure, the residue was purified by column chromatography on silica gel to give 17 (388 mg, 65.0%) as a white solid. *R*_f_ = 0.80 (methanol/dichloromethane = 1:9); ^1^H-NMR (400 MHz, CDCl_3_) δ 8.35 (s, 1H), 7.62 (d, *J* = 8.8 Hz, 2H), 7.50 (d, *J* = 8.8 Hz, 2H), 5.90 (brs, 2H), 4.57 (t, *J* = 6.8 Hz, 2H), 3.42 (t, *J* = 6.8 Hz, 2H), 2.54–2.47 (m, 2H); ^13^C-NMR (125 MHz, CDCl_3_) δ 157.84, 155.94, 154.66, 143.32, 135.36, 131.55, 129.68 (2C), 129.60 (2C), 98.38, 45.55, 32.52, 29.79; HR-MS (FAB^+^) calcd for C_14_H_14_BrClN_5_ [M + H]^+^ 366.0121, found 366.0121.

*4-((2-(2-(4-((3-(4-amino-3-(4-chlorophenyl)-1H-pyrazolo[3,4-d]pyrimidin-1-yl)propyl)amino) phenoxy)ethoxy)ethyl)amino)-2-(2,6-dioxopiperidin-3-yl)isoindoline-1,3-dione(**18a**, CPR-8)*: To the solution of 8a (20 mg, 0.044 mmol) in DMF (3.0 mL) were added potassium carbonate (12.21 mg, 0.066 mmol) and 17 (16.2 mg, 0.044 mmol). The reaction mixture was stirred for 16 h under N_2_ atmosphere at room temperature. The progress of reaction was monitored by TLC. After completion of the reaction, cold water (20 mL) was added and extracted with ethyl acetate (3 x 20 mL). The combined organic layer was dried over anhydrous sodium sulfate. Upon concentration under reduced pressure, the residue was purified by column chromatography on silica gel to give 18a (16.5 mg, 50.6%) as a yellow solid. *R*_f_ = 0.50 (methanol/dichloromethane = 1:9); mp 146.3–147.6 °C; ^1^H-NMR (400 MHz, CDCl_3_) δ 8.35 (s, 1H), 7.63 (d, *J* = 8.4 Hz, 2H), 7.48–7.42 (m, 3H), 7.05 (d, *J* = 6.8 Hz, 1H), 6.89 (d, *J* = 8.4 Hz, 1H), 6.71 (d, *J* = 8.8 Hz, 2H), 6.58 (d, *J* = 8.8 Hz, 2H), 6.46 (t, *J* = 6.0 Hz, 1H), 5.53 (brs, 2H), 4.84 (dd, *J* = 5.2, 12.4 Hz, 1H), 4.45 (td, *J* = 7.2, 2.8 Hz, 2H), 4.03 (t, *J* = 4.4 Hz, 2H), 3.91 (td, *J* = 6.8, 2.4 Hz, 2H), 3.78 (p, 2H), 3.74 (t, *J* = 5.2 Hz, 2H), 3.46 (dd, *J* = 5.6, 11.2 Hz, 2H), 2.88 (dd, *J* = 12.8, 2.8 Hz, 1H), 2.72–2.66 (m, 2H), 2.23 (p, 2H), 2.04–1.99 (m, 1H); ^13^C-NMR (125 MHz, CDCl_3_) δ 170.94, 169.29, 168.75, 167.69, 157.69, 155.86, 154.51, 151.75, 146.77, 142.98, 140.30, 135.93, 135.13, 132.49, 131.75, 129.79 (2C), 129.49 (2C), 116.69, 116.23 (2C), 115.91 (2C), 111.57, 110.37, 98.40, 69.94, 69.71, 68.21, 49.56, 44.84, 42.42, 38.29, 31.96, 27.73, 22.06; HR-MS (FAB^+^) calcd for C_37_H_37_ClN_9_O_6_ [M + H]^+^ 738.2555, found 738.2544.

*4-((2-(2-(2-(4-((3-(4-amino-3-(4-chlorophenyl)-1H-pyrazolo[3,4-d]pyrimidin-1-yl)propyl)amin o)phenoxy)ethoxy)ethoxy)ethyl)amino)-2-(2,6-dioxopiperidin-3-yl)isoindoline-1,3-dione (**18b**, CPR-11)*: 18b was synthesized according to the procedure for 18a. Yellow solid; yield 50.8%; *R*_f_ = 0.45 (methanol/dichloromethane = 1:9); mp 148.2–149.9 °C; ^1^H-NMR (400 MHz, CDCl_3_) δ 8.35 (s, 1H), 7.63 (d, *J* = 8.4 Hz, 2H), 7.49–7.42 (m, 3H), 7.05 (d, *J* = 6.8 Hz, 1H), 6.88 (d, *J* = 8.8 Hz, 1H), 6.70 (d, *J* = 8.8 Hz, 2H), 6.57 (d, *J* = 8.8 Hz, 2H), 6.44 (t, *J* = 5.6 Hz, 1H), 4.82 (dd, *J* = 4.8, 12.0 Hz, 1H), 4.49-4.20 (m, 2H), 4.01 (t, *J* = 4.8 Hz, 2H), 3.93–3.87 (m, 2H), 3.78 (t, *J* = 5.2 Hz, 2H), 3.70–3.63 (m, 6H), 3.43 (dd, *J* = 5.2, 10.8 Hz, 2H), 2.88–2.82 (m, 1H), 2.74–2.62 (m, 2H), 2.23 (p, 2H), 2.06–1.97 (m, 1H); ^13^C-NMR (125 MHz, CDCl_3_) δ 170.94, 169.29, 168.75, 167.70, 157.65, 155.80, 154.49, 151.86, 146.77, 143.01, 140.19, 135.93, 135.14, 132.49, 131.74, 129.79 (2C), 129.50 (2C), 116.68, 116.25 (2C), 115.80 (2C), 111.52, 110.31, 98.39, 70.76, 70.71, 69.97, 69.53, 68.08, 49.55, 44.84, 42.37, 38.26, 31.94, 27.74, 22.04; HR-MS (FAB^+^) calcd for C_39_H_41_ClN_9_O_7_ [M + H]^+^ 782.2817, found 782.2834.

*1-(3-azidopropyl)-3-(4-chlorophenyl)-1H-pyrazolo[3,4-d]pyrimidin-4-amine* (**19**): To the solution of 17 (200 mg, 0.545 mmol) in DMF (2.0 mL) was added sodium azide (46.10 mg, 0.709 mmol) at room temperature. The reaction mixture was stirred for 16 h under N_2_ atmosphere at room temperature. The progress of reaction was monitored by TLC. After completion of the reaction, cold water (50 mL) was added and extracted with ethyl acetate (3 x 20 mL). The combined organic layer was dried over anhydrous sodium sulfate. Upon concentration under reduced pressure, the residue was purified by column chromatography on silica gel to give 19 (119 mg, 66.1%) as a white solid. *R*_f_ = 0.50 (EtOAc/n-Hexane = 1:1); ^1^H-NMR (400 MHz, CDCl_3_) δ 8.36 (s, 1H), 7.62 (d, *J* = 8.0 Hz, 2H), 7.50 (d, *J* = 8.4 Hz, 2H), 5.90 (brs, 2H), 4.52 (t, *J* = 6.4 Hz, 2H), 3.36 (t, *J* = 6.4 Hz, 2H), 2.23–2.17 (m, 2H); ^13^C-NMR (125 MHz, CDCl_3_) δ 157.84, 155.96, 154.63, 143.31, 135.36, 131.55, 129.67 (2C), 129.60 (2C), 98.34, 48.71, 44.26, 28.93; HR-MS (FAB^+^) calcd for C_14_H_14_ClN_8_ [M + H]^+^ 329.1030, found 329.1033.

*2-(2,6-dioxopiperidin-3-yl)-4-((2-(2-(4-(prop-2-yn-1-ylamino)phenoxy)ethoxy)ethyl)amino)iso indoline-1,3-dione (**20a**):* To the solution of 8a (50 mg, 0.110 mmol) in DMF (3.0 mL) were added potassium carbonate (22.9 mg, 0.165 mmol) and propargyl bromide (14.5 mg, 0.110 mmol) at room temperature. The reaction mixture was stirred for 16 h under N_2_ atmosphere at room temperature. The progress of reaction was monitored by TLC. After completion of the reaction, cold water (20 mL) was added and extracted with ethyl acetate (3 x 20 mL). The combined organic layer was dried over anhydrous sodium sulfate. Upon concentration under reduced pressure, the residue was purified by column chromatography on silica gel to give 20a (30 mg, 55.4%) as a yellow solid. *R*_f_ = 0.60 (ethyl acetate = 100%); ^1^H-NMR (400 MHz, CDCl_3_) δ 7.45 (t, *J* = 8.4 Hz, 1H), 7.07 (d, *J* = 7.2 Hz, 1H), 6.91 (d, *J* = 8.4 Hz, 1H), 6.73 (d, *J* = 8.8 Hz, 2H), 6.59 (d, *J* = 8.8 Hz, 2H), 6.49 (t, *J* = 5.6 Hz, 1H), 4.92 (dd, *J* = 7.6, 12.4 Hz, 1H), 4.55 (d, *J* = 2.4 Hz, 2H), 4.04 (t, *J* = 4.4 Hz, 2H), 3.81–3.74 (m, 4H), 3.47 (dd, *J* = 5.2, 10.8 Hz, 2H), 3.00–2.93 (m, 1H), 2.81–2.72 (m, 2H), 2.14 (s, 1H), 2.08–2.04 (m, 1H); ^13^C-NMR (125 MHz, CDCl_3_) δ 170.11, 169.23, 167.96, 167.63, 151.77, 146.81, 140.30, 135.97, 132.46, 116.75, 116.23 (2C), 115.92 (2C), 111.61, 110.33, 77.94, 70.76, 69.94, 69.70, 68.22, 49.52, 42.42, 31.87, 29.62, 21.92; HR-MS (FAB^+^) calcd for C_26_H_27_N_4_O_6_ [M + H]^+^ 491.1931, found 491.1938.

*2-(2,6-dioxopiperidin-3-yl)-4-((2-(2-(2-(4-(prop-2-yn-1-ylamino)phenoxy)ethoxy)ethoxy)ethyl) amino)isoindoline-1,3-dione (**20b**)*: 20b was synthesized according to the procedure for 20a. White solid; yield 52.5%; *R*_f_ = 0.60 (EtOAc/n-Hexane = 1:1); ^1^H-NMR (400 MHz, CDCl_3_) δ 7.45 (t, *J* = 8.4 Hz, 1H), 7.07 (d, *J* = 6.8 Hz, 1H), 6.90 (d, *J* = 8.4 Hz, 1H), 6.72 (d, *J* = 8.8 Hz, 2H), 6.59 (d, *J* = 8.8 Hz, 2H), 6.47 (t, *J* = 5.6 Hz, 1H), 4.87 (dd, *J* = 4.8, 7.2 Hz, 1H), 4.55 (d, *J* = 2.4 Hz, 2H), 4.03 (t, *J* = 4.8 Hz, 2H), 3.81 (t, *J* = 5.2 Hz, 2H), 3.72–3.66 (m, 6H), 3.44 (dd, *J* = 5.6, 11.2 Hz, 2H), 2.97–2.86 (m, 1H), 2.76–2.67 (m, 2H), 2.15 (s, 1H), 2.08–2.01 (m, 1H); ^13^C-NMR (125 MHz, CDCl_3_) δ 170.10, 169.23, 167.95, 167.66, 151.90, 146.81, 140.20, 135.98, 132.48, 116.74, 116.26 (2C), 115.82 (2C), 111.58, 110.30, 77.97, 70.77, 70.74, 69.99, 69.57, 69.51, 68.11, 49.51, 42.39, 31.85, 29.63, 21.93; HR-MS (FAB^+^) calcd for C_28_H_31_N_4_0_7_ [M + H]^+^ 535.2193, found 535.2200.

*4-((2-(2-(4-(((1-(3-(4-amino-3-(4-chlorophenyl)-1H-pyrazolo[3,4-d]pyrimidin-1-yl)propyl)-1H-1,2,3-triazol-4-yl)methyl)amino)phenoxy)ethoxy)ethyl)amino)-2-(2,6-dioxopiperidin-3-yl)isoindol ine-1,3-dione (**21a**, CPR-13)*: 21a was synthesized according to the procedure for 16a. Yellow solid; yield 40.1%; *R*_f_ = 0.45 (methanol/dichloromethane = 1:9); mp 159.1–160.6 °C; ^1^H-NMR (400 MHz, CDCl_3_) δ 8.35 (s, 1H), 7.69 (s, 1H), 7.61 (d, *J* = 8.4 Hz, 2H), 7.48 (d, *J* = 8.4 Hz, 2H), 7.42 (t, *J* = 7.2 Hz, 1H), 7.01 (d, *J* = 7.2 Hz, 1H), 6.88 (d, *J* = 8.4 Hz, 1H), 6.70 (d, *J* = 8.8 Hz, 2H), 6.57 (d, *J* = 8.8 Hz, 2H), 6.47 (t, *J* = 5.6 Hz, 1H), 5.13-5.04 (m, H), 4.92 (dd, *J* = 5.2, 12.4 Hz, 1H), 4.47 (t, *J* = 7.2 Hz, 2H), 4.34 (t, *J* = 6.8 Hz, 2H), 4.03 (t, *J* = 4.8 Hz, 2H), 3.78 (t, *J* = 4.8 Hz, 2H), 3.74 (t, *J* = 5.6 Hz, 2H), 3.44 (dd, *J* = 4.4, 10.0 Hz, 2H), 2.97–2.90 (m, 1H), 2.79–2.68 (m, 2H), 2.53 (p, 2H), 2.07-2.00 (m, 1H); ^13^C-NMR (125 MHz, CDCl_3_) δ 170.52, 169.25, 168.70, 167.80, 167.68, 159.35, 157.61, 155.89, 154.58, 146.79, 146.73, 143.55, 143.07, 135.94, 135.40, 132.46, 131.37, 129.69 (2C), 129.62 (2C), 123.83, 116.74, 116.44 (2C), 115.90 (2C), 111.56, 110.33, 69.92, 69.68, 68.23, 49.54, 47.58, 44.12, 42.39, 35.62, 31.91, 29.98, 22.04; HR-MS (FAB^+^) calcd for C_40_H_40_ClN_12_O_6_ [M + H]^+^ 819.2882, found 819.2887.

*4-((2-(2-(2-(4-(((1-(3-(4-amino-3-(4-chlorophenyl)-1H-pyrazolo[3,4-d]pyrimidin-1-yl)propyl)-1H-1,2,3-triazol-4-yl)methyl)amino)phenoxy)ethoxy)ethoxy)ethyl)amino)-2-(2,6-dioxopiperidin-3-yl)isoindoline-1,3-dione (**21b**, CPR-14)*: 21b was synthesized according to the procedure for 16a. Yellow solid; yield 38.3%; *R*_f_ = 0.250 (methanol/dichloromethane = 1:9); mp 164.5–165.9 °C; ^1^H-NMR (400 MHz, CDCl_3_) δ 8.35 (s, 1H), 7.69 (s, 1H), 7.61 (d, *J* = 8.0 Hz, 2H), 7.48 (d, *J* = 8.4 Hz, 2H), 7.41 (t, *J* = 7.6 Hz, 1H), 6.99 (d, *J* = 7.2 Hz, 1H), 6.87 (d, *J* = 8.8 Hz, 1H), 6.69 (d, *J* = 8.4 Hz, 2H), 6.57 (d, *J* = 8.4 Hz, 2H), 6.45 (t, *J* = 6.0 Hz, 1H), 5.12–5.03 (m, 2H), 4.88 (dd, *J* = 5.2, 11.6 Hz, 1H), 4.47 (t, *J* = 6.0 Hz, 2H), 4.36 (t, *J* = 7.2 Hz, 2H), 4.01 (t, *J* = 5.2 Hz, 2H), 3.79 (t, *J* = 5.2 Hz, 2H), 3.71-3.65 (m, 6H), 3.44–3.40 (m, 2H), 2.93–2.87 (m, 1H), 2.75–2.64 (m, 2H), 2.55 (p, 2H), 2.04–1.98 (m, 1H); ^13^C-NMR (125 MHz, MeOD+CDCl_3_) 170.75, 169.10, 168.74, 167.77, 155.58, 154.17, 146.67, 146.60, 135.90, 135.39, 135.15, 133.28, 132.35, 132.27, 131.00, 130.89, 129.57 (2C), 129.51 (2C), 128.92, 128.67, 116.72 (2C), 111.40 (2C), 109.99, 109.50, 70.58, 70.52, 69.82, 69.37, 68.01, 47.62, 44.02, 42.09, 35.33, 31.62, 29.80, 29.54, 21.90; HR-MS (FAB^+^) calcd for C_42_H_44_ClN_12_O_7_ [M + H]^+^ 863.3144, found 863.3149.

### 4.2. Anticancer Activity

#### 4.2.1. MTT Assay

MCF7 (human breast cancer) and A549 (human lung cancer) cells were obtained from the Korean Cell Line Bank (KCLB, Seoul, Korea) and incubated in 5% CO_2_ at 37 °C. The cells were plated in 96-well culture plates (SPL Life Science Co., Gyeonggi-do, Republic of Korea) for 24 h. For cell viability test, the synthesized PROTACs were treated at the various concentrations from 0 to 80 μM for 3 days. After that, 100 μL of 20 mM MTT (3-(4,5-Dimethylthiazol-2-yl)-2,5-Diphenyltetrazolium Bromide) (Thermo Fisher Scientific, Rockford, IL, USA) (5 mg/mL) dissolved in RPMI-1640 medium was added to the incubated well for 3 h. The supernatant was discarded and 100 μL of DMSO was reacted with viable cells. The product of reaction was measured by absorbance at 570 nm using Mithras2 plate reader (Berthold Technologies, Bad Wildbad, Germany).

#### 4.2.2. Western Blot Assay

The PROTAC-treated MCF7 (human breast cancer) and A549 (human lung cancer) cells were lysed with 1X lysis buffer containing protease inhibitor. Extracted protein lysate (20 μg) were loaded on 12% SDS-polyacrylamide gel electrophoresis and transferred to a polyvinylidene difluoride membrane (ATTO, Tokyo, Japan). The membrane was blocked with 3% Bovine Serum Albumin (BSA) in tris-phosphate buffer containing 0.1% Twin 20, and incubated with primary antibodies for 1 h at room temperature. The following antibodies were IGF1Rβ (1:200, Santa Cruz Biotechnology, Santa Cruz, CA, USA) and Src (1:000, Cell Signaling Technology, Beverly, MA, USA) and GAPDH (1:200, Santa Cruz Biotechnology) as internal control. The signal was reacted with ECL solution (GenDEPOT, Barker, TX, USA) and detected on an Image Quant LAS 4000 biomolecular imager (GE Healthcare, Chicago, Il, USA).

#### 4.2.3. Wound Healing and Invasion Assay

MCF7 (human breast cancer) and A549 (human lung cancer) cells were treated with 1 or 5 μM of PROTAC compounds for 24 h. After 24 h, the treated cells were collected and re-implanted (3 × 10^5^) on 24-well culture plates for 24 h. When cells reached confluence, wound healing assay was performed by scratching with pipette tips. For invasion assay, matrigel (BD Biosciences, San Diego, CA, USA) coated transwell (BD Biosciences, San Diego, CA, USA, 353097, 8 µm pore size) was used to mimic extra cellular matrix in vitro. The PROTAC-treated cells (1 × 10^5^) suspended in RPMI-1640 without FBS were seeded into transwell and each of the insert wells was put in 24-well culture plates containing RPMI-1640 with FBS. After incubation, the membrane of insert transwell was stained with 0.1% crystal violet (Sigma Aldrich, St. Louis, USA). The wound closure or invasive images were analyzed based on bright field microscopy.

#### 4.2.4. Soft Agar Colony Formation Assay

To investigate growth ability on solid surface in vitro, a colony forming assay was performed by using top and bottom agarose method. The base of layer was poured with 1.5 mL of 0.5% agarose (BD Biosciences, San Diego, CA, USA) containing RPMI-1640 with FBS and solidified. 1 mL of PROTAC-treated cells (3 × 10^5^) resuspended in complete medium with 0.3% agarose (BD Biosciences, San Diego, CA, USA) was covered on the base of layer. The plates were incubated for approximately 2 weeks in 5% CO_2_ at 37 °C. Formed colony was counted on bright field microscopy.

## 5. Conclusions

We developed dual degrader PROTACs to target both IGF-1R and Src, which are associated with various cancer cells. We evaluated the degradation potentials of the synthesized PROTAC compounds by different cellular assays. Interestingly, PROTACs with a dual IGF-1R/Src inhibitor warhead (**C**), which possess a common structure from the reported IGF-1R and Src inhibitors, showed significant degradation potency, whereas previously reported individual modules, with similar sizes for Src or IGF-1R, were not active in PROTACs. Our data support the notion that the binding affinities of warhead ligands are not the sole determinant of PROTAC efficiency. Off-target effects, with promiscuous ligands, may be counteracted with other factors, including the formation of a stable ternary complex with target protein-E3 ligase, as well as efficient ubiquitination. Our dual degrader PROTACs, along with various cell-based assays, indicated that the PROTACs developed in this study serve as valuable tools to understand the mechanism and process of induced degradation of target proteins, which will result in next-generation anticancer therapeutics.

## Supplementary Materials

The following are available online: Experimental details, synthesis of intermediates (7a–c, 9, 11a–b, 15, 17 and 19), ^1^H and ^13^C-NMR spectra of the synthesized compounds.
