# Supplementary material for: Design, Synthesis, and Biological Evaluation of Proteolysis Targeting Chimeras (PROTACs) for the Dual Degradation of IGF-1R and Src
Source: Molecules. 2020 Apr 23;25(8):1948. doi: 10.3390/molecules25081948 (PMC7221895; doi:10.3390/molecules25081948)
Supplement: Supplementary file 1 [file molecules-25-01948-s001.pdf]

Supporting information

Design, Synthesis, and Biological Evaluation of Proteolysis Targeting Chimeras (PROTACs) for the Dual Degradation of IGF-1R and Src

Sudhakar Manda [1,†], Na Keum Lee [1,†], Dong-Chan Oh [2] and Jeeyeon Lee [1,*]

[1]College of Pharmacy, Research Institute of Pharmaceutical sciences, Seoul National University, 1 Gwanak-ro, Gwanak-gu, Seoul 08826, Republic of Korea

[2]Natural Products Research Institute, College of Pharmacy, Seoul National University, Seoul 08826, Republic of Korea

† These authors contributed equally to this work

* Correspondence: jyleeut@snu.ac.kr; Tel.: +82-02-880-2471

Table of Contents

1. Synthesis of **7a-c, 9, 11a-b, 15, 17** and **19** S2 – S6

2. ^{1}H and ^{13}C NMR spectra S8-S28

3. Reference S29

1. Synthesis of 7a-c, 9, 11a-b, 15, 17 and **19:**

General procedure for synthesis of 2a-b:

2-(2-azidoethoxy)ethanol (2a)**:** Sodium azide (2.60 g, 40.138 mmol, 2.5 eq.) was added to the solution of 2-(2-chlorethoxy)ethanol **1a** (2 g, 16.055 mmol, 1.0 eq.) in water (20 mL). The reaction was stirred at 90 ºC for 16 h, then poured into sodium hydroxide solution (5%, 50 mL) and extracted with diethyl ether (2 x 50 mL). The organic layer was dried over Na_2SO_4 and evaporated until completely dried to afford 2-(2-azidoethoxy)ethanol **2a** (1.74 g, 82.66%) as a colorless oil. R_f = 0.50 (EtOAc/*n*-hexane=3:7); ^{1}H NMR (400MHz, $CDCl_3$) δ 3.75-3.71 (m, 2H), 3.67 (t, *J* = 8.0 Hz, 2H), 3.59 (t, *J* = 4.0 Hz, 2H), 3.39 (t, *J* = 4.0 Hz, 2H)

2-(2-(2-azidoethoxy)ethoxy)ethanol(2b)**: 2b** was synthesized according to the procedure for **2a.** Colorless oil; yield 69.56%; R_f = 0.3 (EtOAc/*n*-hexane = 3:7); ^{1}H NMR (400 MHz, $CDCl_3$) δ 3.72 (t, *J* = 8.0 Hz, 4H), 3.59 (t, *J* = 8.0 Hz, 4H), 3.37 (t, *J* = 8.0 Hz, 4H), 2.51 (t, *J* = 4.0 Hz, 2H).

1-(2-(2-azidoethoxy)ethoxy)-4-nitrobenzene (4a): To the solution of **2a** (1.6 g, 12.201 mmol) in DMSO, were added 4-fluoro nitrobenzene **3** (1.72 g, 12.201 mmol) and K_2CO_3 (1.68 g, 12.201 mmol). The resulting mixture was stirred for 4 h at 80 °C, which was cooled down to room temperature. 50 mL of cold water was added and extracted with ethyl acetate (3 x 50 mL). The combined organic layer was dried over anhydrous sodium sulfate. Upon concentration under reduced pressure, the residue was purified by column chromatography on silica gel to give **4a** (1.63 g, 53.01%) as a yellow syrup. R_f = 0.40 (EtOAc/*n*-hexane = 3:7); ^{1}H NMR (400 MHz, $CDCl_3$) δ 8.18 (d, *J* = 8.0 Hz, 2H), 6.97 (d, *J* = 12.0 Hz, 2H), 4.22 (t, *J* = 8.0 Hz, 2H), 3.89 (d, *J* = 4.0 Hz, 2H), 3.74 (t, *J* = 4.0 Hz, 2H), 3.41 (t, *J* = 4.0 Hz, 2H).

1-(2-(2-(2-azidoethoxy)ethoxy)ethoxy)-4-nitrobenzene (4b): **4b** was synthesized according to the procedure for **4a**. Yellow colored syrup; yield 61.6%; R_f = 0.250 (EtOAc/*n*-hexane = 1:1); ^{1}H NMR (400 MHz, $CDCl_3$) δ 8.18 (d, *J* = 9.6 Hz, 2H), 6.96 (d, *J* = 9.2 Hz, 2H), 4.21 (t, *J* =

4.8 Hz, 2H), 3.89 (t, J = 4.8 Hz, 2H), 3.73-3.65 (m, 6H), 3.37 (t, J = 4.0 Hz, 1H).

Synthesis of 1-(2-(2-(2-(2-azidoethoxy)ethoxy)ethoxy)ethoxy)-4-nitrobenzene (4c): **4c** was synthesized according to the procedure described in the previous reports (Scheme S1) (1). Tosylation of 2,2'-((oxybis(ethane-2,1-diyl))bis(oxy))diethanol **23** in presence of basic medium produced **24,** which reacted with 4-nitro phenol to yield **25**. Sodium azide was reacted with **25** in ethanol to produce azido nitrophenol intermediate **4c**.

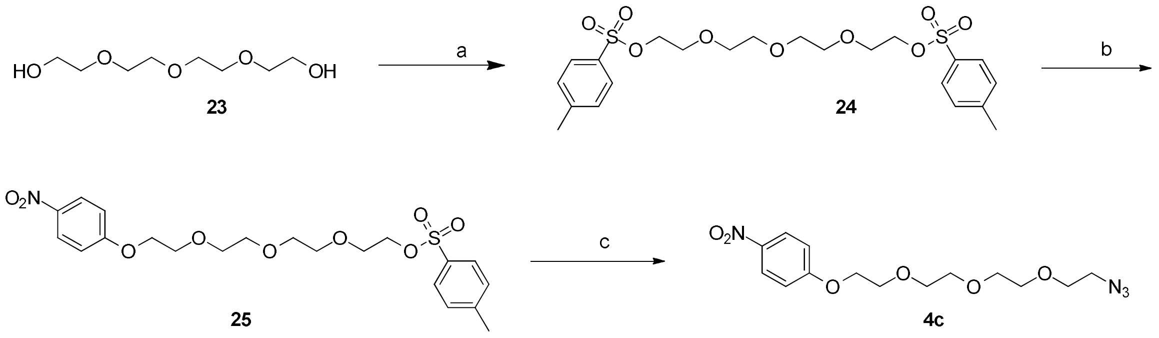

Scheme S1. Reagents and conditions: a) p-TsCl, KOH, H_2O, THF, 0 °C-RT, 5 h, 87%; b) 4-nitro phenol, K_2CO_3, DMF, 50 °C, 16 h, 76%; c) NaN_3, EtOH, reflux, 16 h, 47%.

2-(2-(4-nitrophenoxy)ethoxy)ethanamine (5a): A reaction mixture of **4a** (1.5 g, 5.950 mmol) and triphenylphosphine (1.71 g, 6.545 mmol) were dissolved in THF (15 mL) and water (1.5 mL). The mixture was stirred for 16 h under N_2 atmosphere at room temperature. The progress of reaction was monitored by TLC. After concentration of the reaction mixture under reduced pressure, the crude mixture was purified by column chromatography on silica gel to give **5a** (1.14 g, 85.08%) as a yellow syrup. R_f = 0.05 (EtOAc/*n*-hexane = 3:7); ^{1}H NMR (400 MHz, $CDCl_3$) δ 8.18 (d, J = 9.2 Hz, 2H), 6.97 (d, J = 9.6 Hz, 2H), 4.21 (t, J = 4.4 Hz, 2H), 3.85 (t, J = 4.8 Hz, 2H), 3.58 (t, J = 5.2 Hz, 2H), 2.90 (t, J = 5.2 Hz, 2H), 1.91 (brs, 2H).

2-(2-(2-(4-nitrophenoxy)ethoxy)ethoxy)ethanamine (5b)**:** **5b** was synthesized according to the procedure for **5a**. Yellow colored syrup; yield 86.91%; R_f = 0.250 (EtOAc/*n*-hexane = 1:1); ^{1}H NMR (400 MHz, $CDCl_3$) δ 8.18 (d, J = 9.2, Hz, 2H), 6.96 (d, J = 9.2 Hz, 2H), 4.21 (t, J = 4.4 Hz, 2H), 3.88 (t, J = 4.8 Hz, 2H), 3.73-3.70 (m, 2H), 3.69-3.62 (m, 2H), 3.51 (t, J = 5.2

Hz, 2H), 2.86 (t, J = 5.2 Hz, 2H), 1.86 (s, 2H).

***2-(2-(2(2-(4-nitrophenoxy)ethoxy)ethoxy)ethoxy)ethanamine (5c)*: 5c** was synthesized according to the procedure for **5a**. Yellow colored syrup; yield 62.5%; R_f = 0.250 (EtOAc/*n*-hexane = 1:1); ^{1}H NMR (400 MHz, $CDCl_3$) δ 8.18 (d, J = 9.2 Hz, 2H), 6.96 (d, J = 9.2 Hz, 2H), 4.21 (t, J = 4.8 Hz, 2H), 3.88 (t, J = 4.8 Hz, 2H), 3.73-3.59 (m, 10H), 3.49 (t, J = 4.8 Hz, 2H).

2-(2,6-dioxopiperidin-3-yl)-4-fluoroisoindoline-1,3-dione (6): The intermediates 4-fluorophthalic anhydride (**27**) and 2-(2,6-dioxopiperidin-3-yl)-4-fluoroisoindoline-1,3-dione (**6**) were synthesized according to the procedure described in the previous reports (2). 3-Fluorophthalic acid **26** was refluxed in acetic anhydride to give 3-fluoro phthalic anhydride **27**, which was treated with 3-aminopiperidine-2,6-dione hydrochloride in acetic acid to obtain intermediate **6** (Scheme S2).

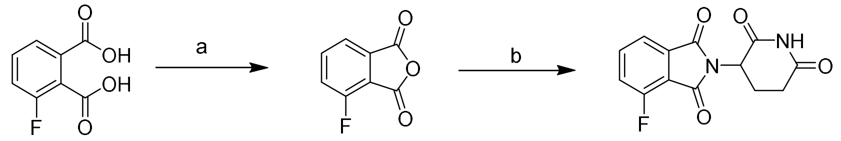

26 **27** **6**

Scheme S2. Reagents and conditions: a) Acetic anhydride, 140 °C, 5 h, 92%; b) 3-Aminopiperidine-2,6-dione hydrochloride, NaOAc, AcOH, reflux, 12 h, 75%.

General procedure for synthesis of 7a-c: To a stirred solution of **6a-c** (1.272 mmol) in DMF (5mL) was added diisopropyl ethylamine (2.545 mmol), and the reaction mixture was stirred for 16 h at 90 °C. After completion of the reaction, the reaction mixture was cooled down to room temperature, and 50 mL of cold water was added and extracted with ethyl acetate (3 x 50 mL). The combined organic layer was dried over anhydrous sodium sulfate. Upon concentration under reduced pressure, the residue was purified by column chromatography on silica gel to give crude **7a-c**, which were directly used for next step without further purification.

Synthesis of 9 and 11a-b: The intermediates *N*-(2-chloropyrimidin-4-yl)quinolin-3-amine (**9**),

2-chloro-*N*-(5-methyl-1*H*-pyrazol-3-yl)pyrimidin-4-amine (**11a**) and 2,5-dichloro-*N*-(5-methyl-1*H*-pyrazol-3-yl)pyrimidin-4-amine (**11b**) were synthesized according to the procedure described in the previous reports (3). 2,4-dichloropyrimidine **28a** reacted with 3-amino quinolin or 3-amino 5-methyl pyrazole in the presence of diisopropyl ethylamine in isopropanol to obtain **9** and **11a**, respectively (4). In the same way 2,4,5-trichloropyrimidine **28b** was treated with 3-amino 5-methyl pyrazole in butanol to produce **11b** (Scheme S3) (5).

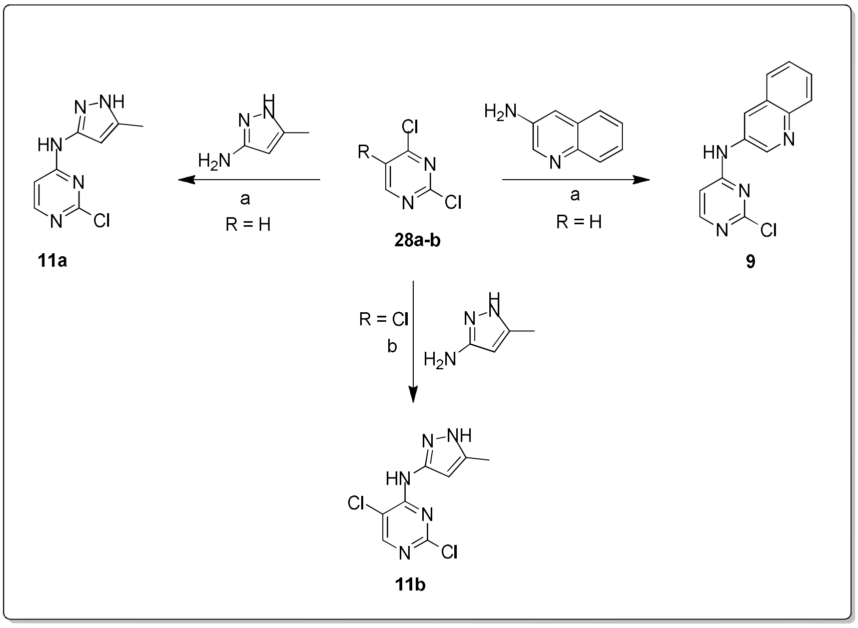

Scheme S3. Reagents and conditions: a) DIPEA, isopropanol, refluxed 100 °C, 16 h, 41-70%; b) p-toluenesulfonic acid monohydrate, butanol, DIEA, 100 °C, 16 h, 78%.

Synthesis of 15-17. The 4-aminopyrazolo[3,4-*d*]pyrimidines modules **15** were synthesized according to the reported procedure with slight modification as follows (Scheme S4) (6,7). 4-chlorobenzoyl chloride **29** was reacted with malononitrile **30** in the presence of sodium hydride in anhydrous THF to yield **31**. Subsequent methylation with dimethyl sulfate in the presence of sodium bicarbonate in dioxane-water mixtures afforded **32**, which was cyclized to **33** by reacting with hydrazine monohydrate in ethanol. **33** reacted with formamide at 180 °C for 2 h to give **22.** Further *N*-alkylation in the presence of K_2CO_3 was proceeded to obtain key intermediates **15.**

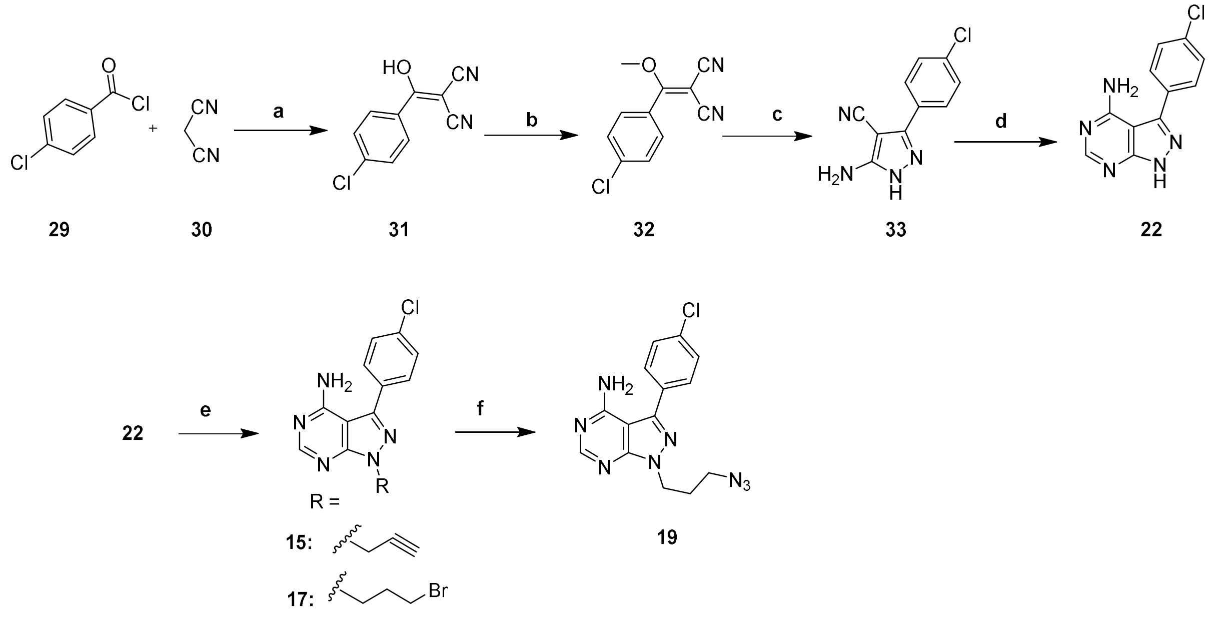

Scheme S4. Reagents and conditions: a) NaH, dry THF, 0 °C to RT, 1 h, 60%; b) Me_2SO_4, $NaHCO_3$, 1,4 dioxane: H_2O, 80 ºC, 2 h, 58%; c) $NH_2NH_2.H_2O$, EtOH, 80 ºC, 1 h, 90.01%; d) Formamide, 180 ºC, 2 h, 60.0%; e) alkyl bromide, K_2CO_3, DMF, RT, 3 h, 65.01%; f) NaN_3, DMF, RT, 16 h, 66.5%.

2. ^{1}H- and ^{13}C-NMR spectra

^{1}H NMR of compound **8a** (400 MHz, $CDCl_3$)

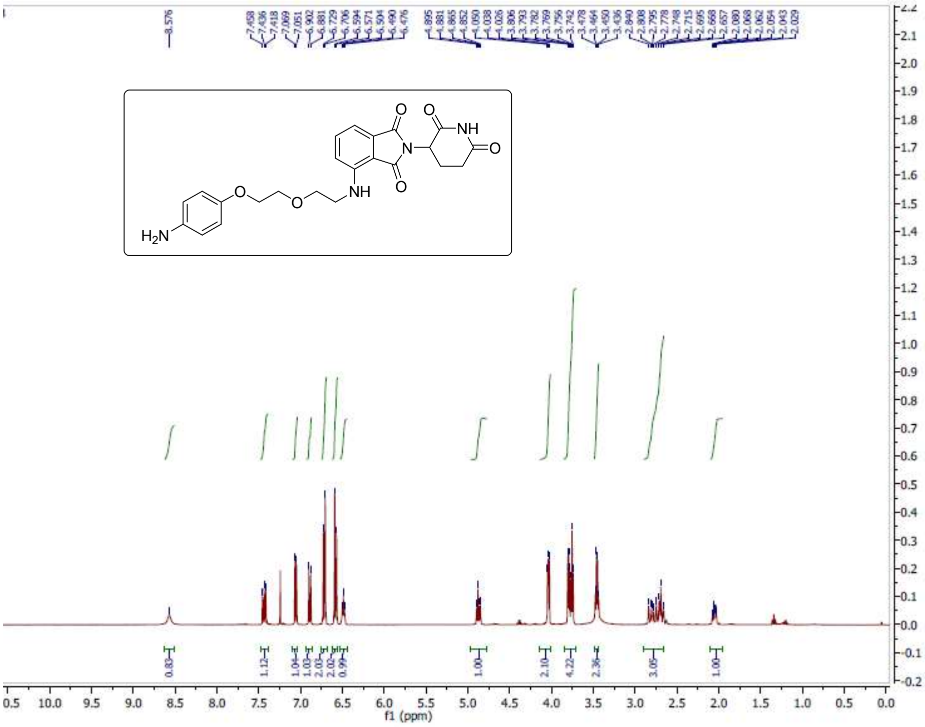

^{13}C NMR of compound **8a** (125 MHz, $CDCl_3$)

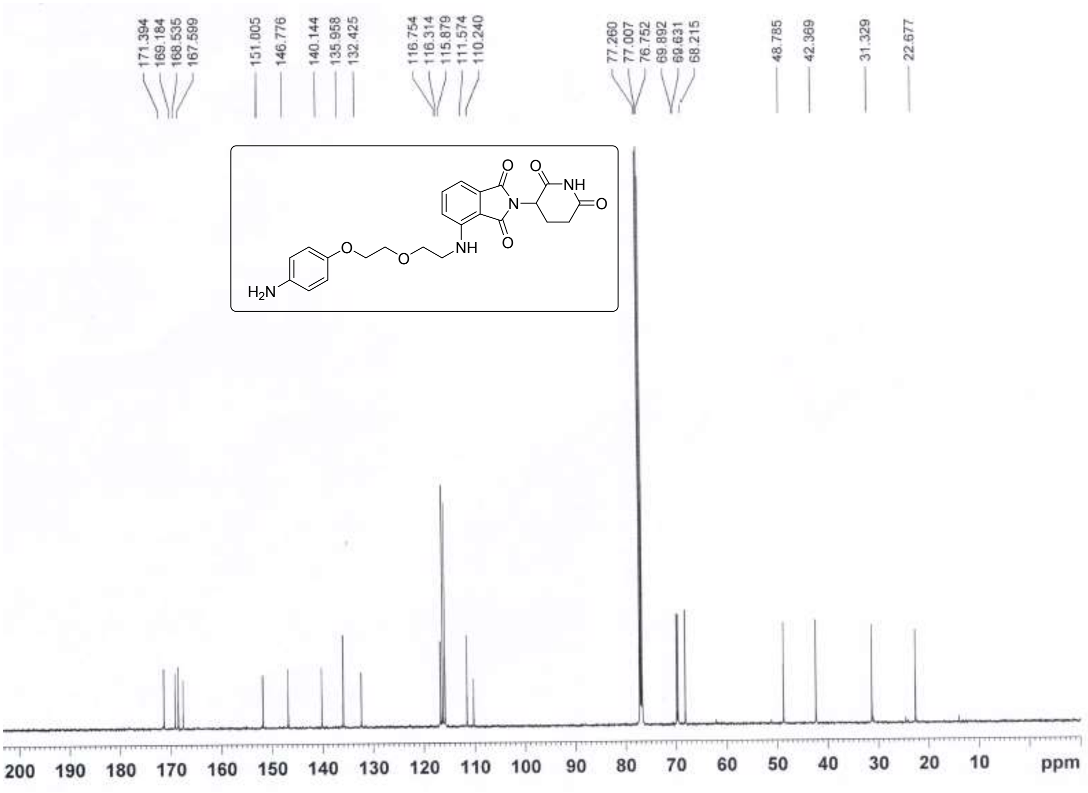

^{1}H NMR of compound **8b** (400 MHz, $CDCl_3$)

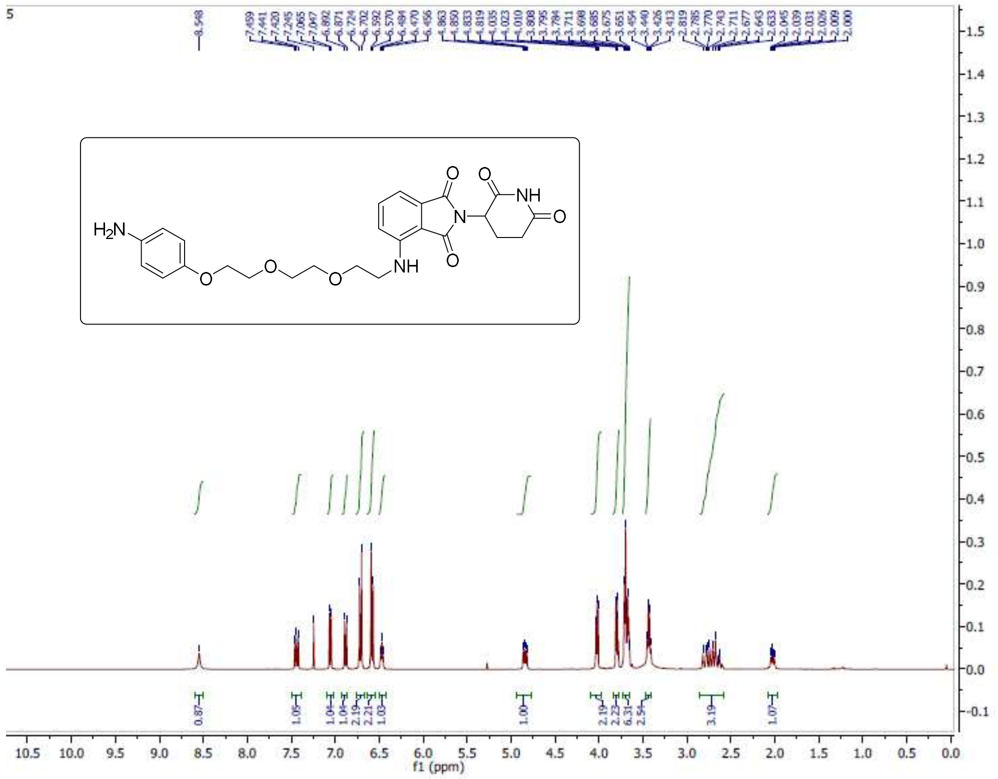

^{13}C NMR of compound **8b** (125 MHz, $CDCl_3$)

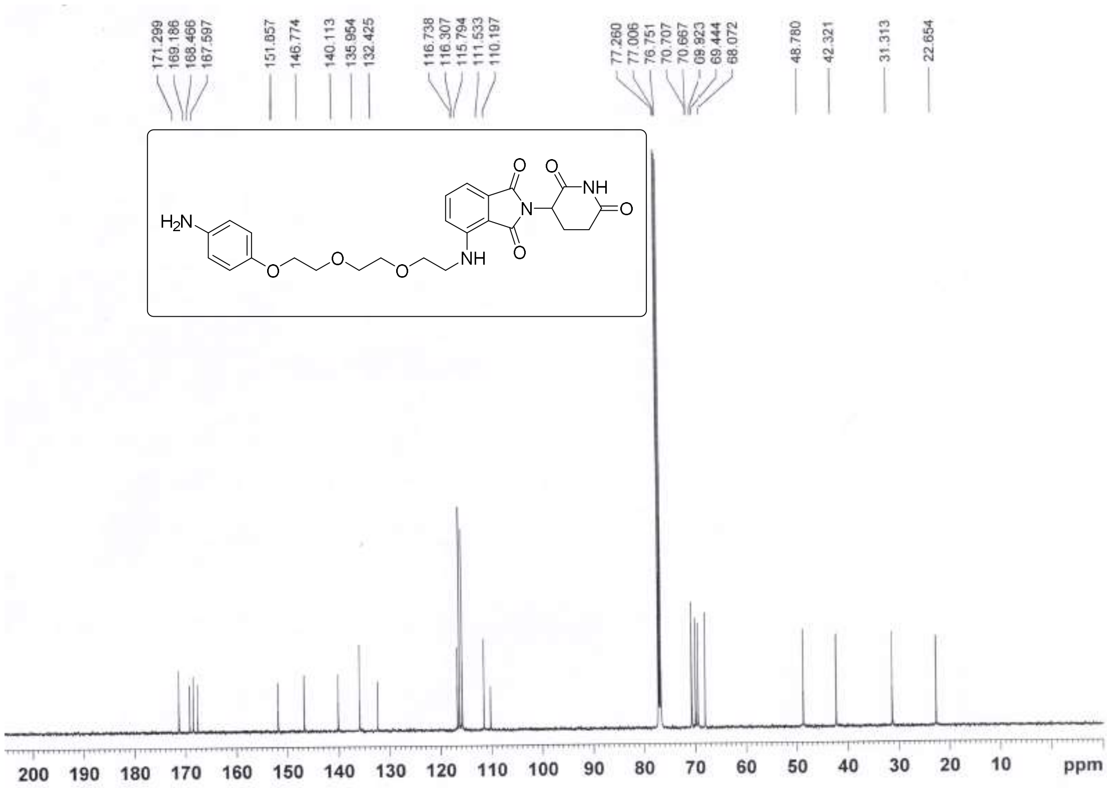

^{1}H NMR of compound **8c** (400 MHz, $CDCl_3$)

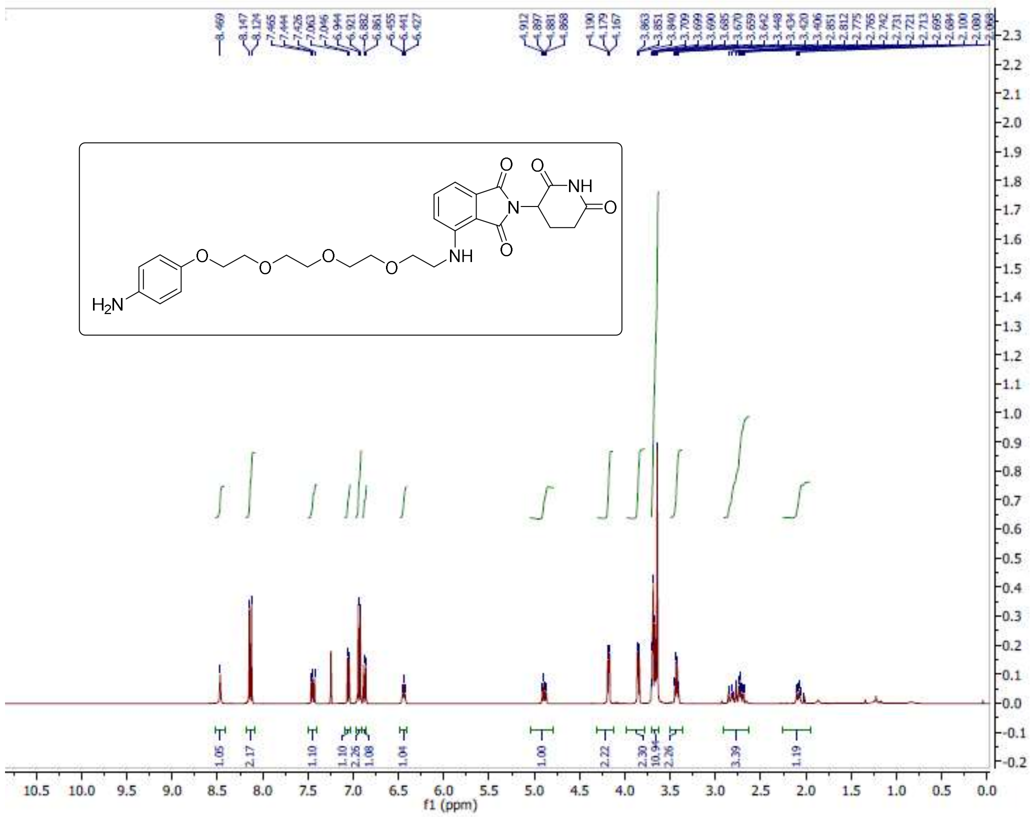

^{13}C NMR of compound **8c** (125 MHz, $CDCl_3$)

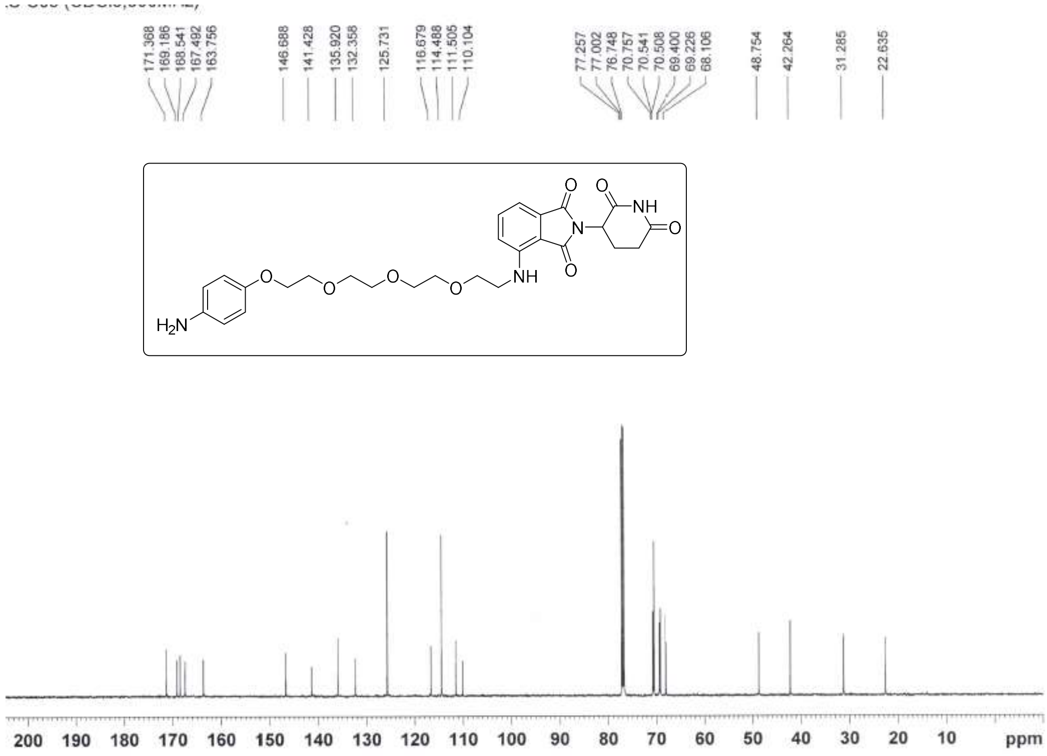

^{1}H NMR of compound **14a** (400 MHz, $CDCl_3$)

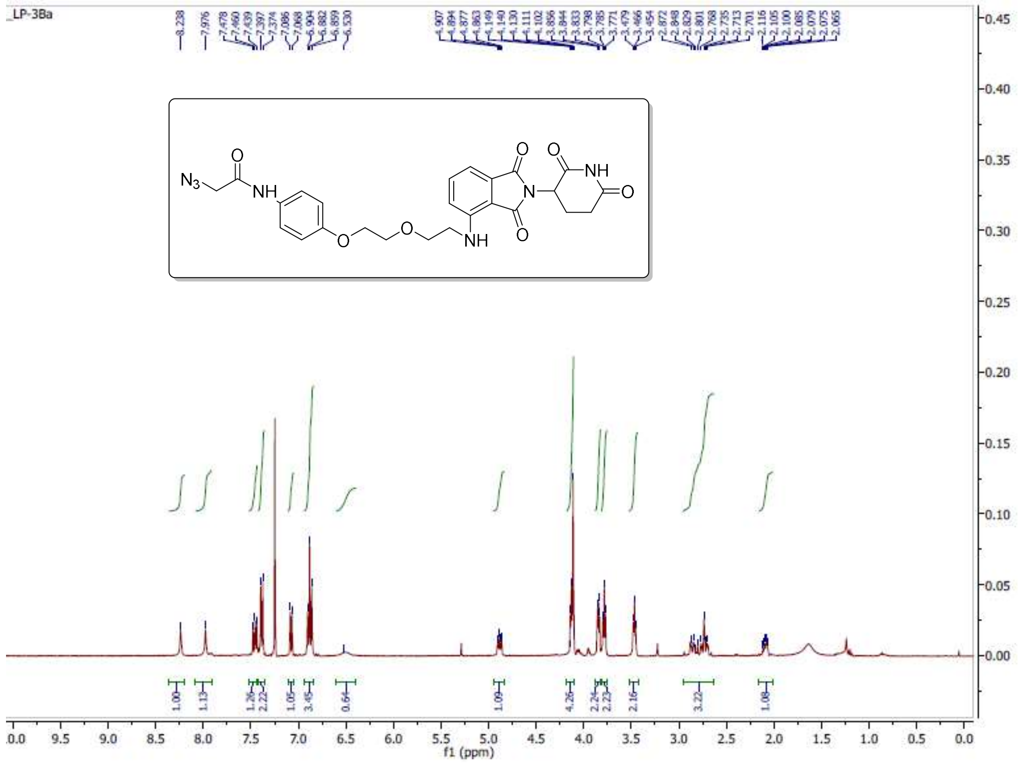

^{13}C NMR of compound **14a** (125 MHz, $CDCl_3$)

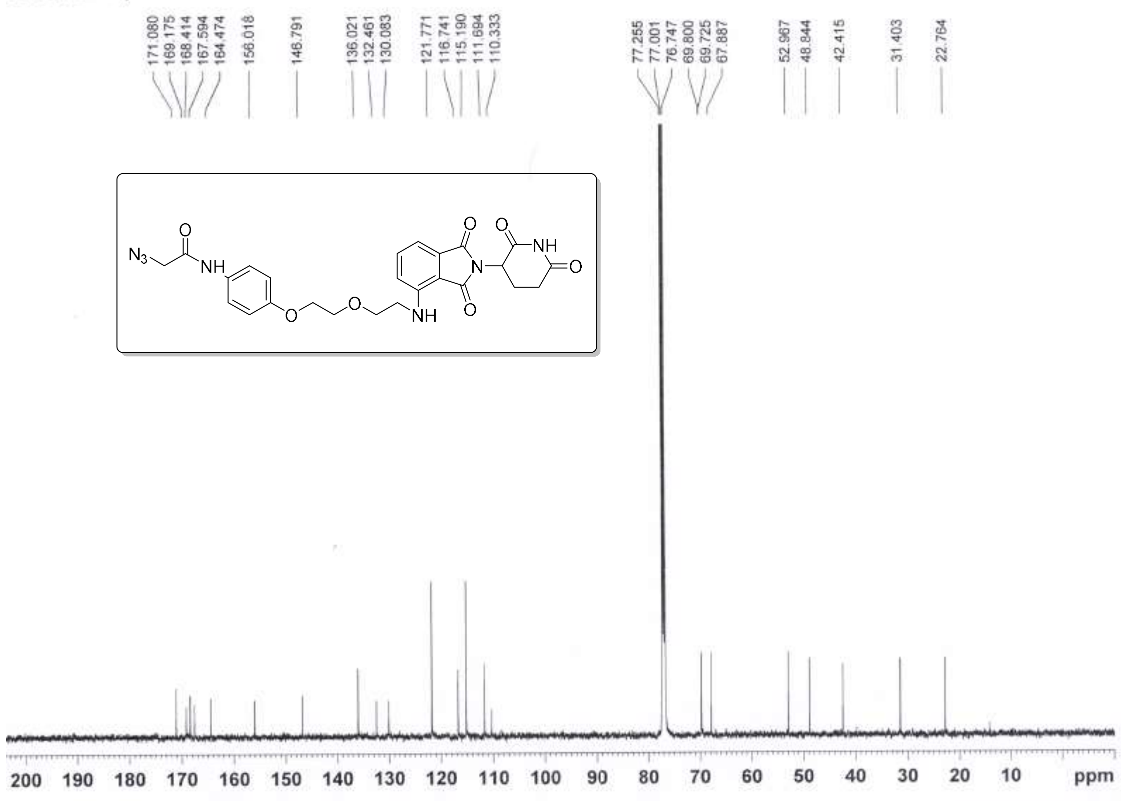

^{1}H NMR of compound **14b** (400 MHz, $CDCl_3$)

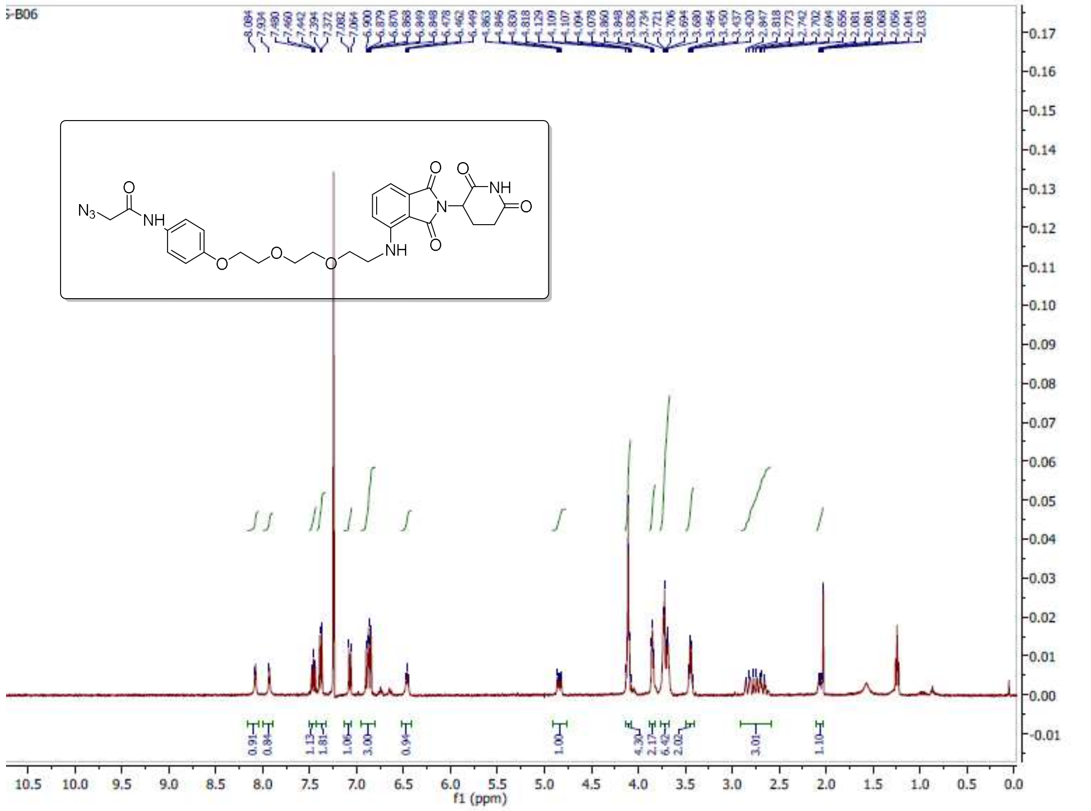

^{13}C NMR of compound **14b** (125 MHz, $CDCl_3$)

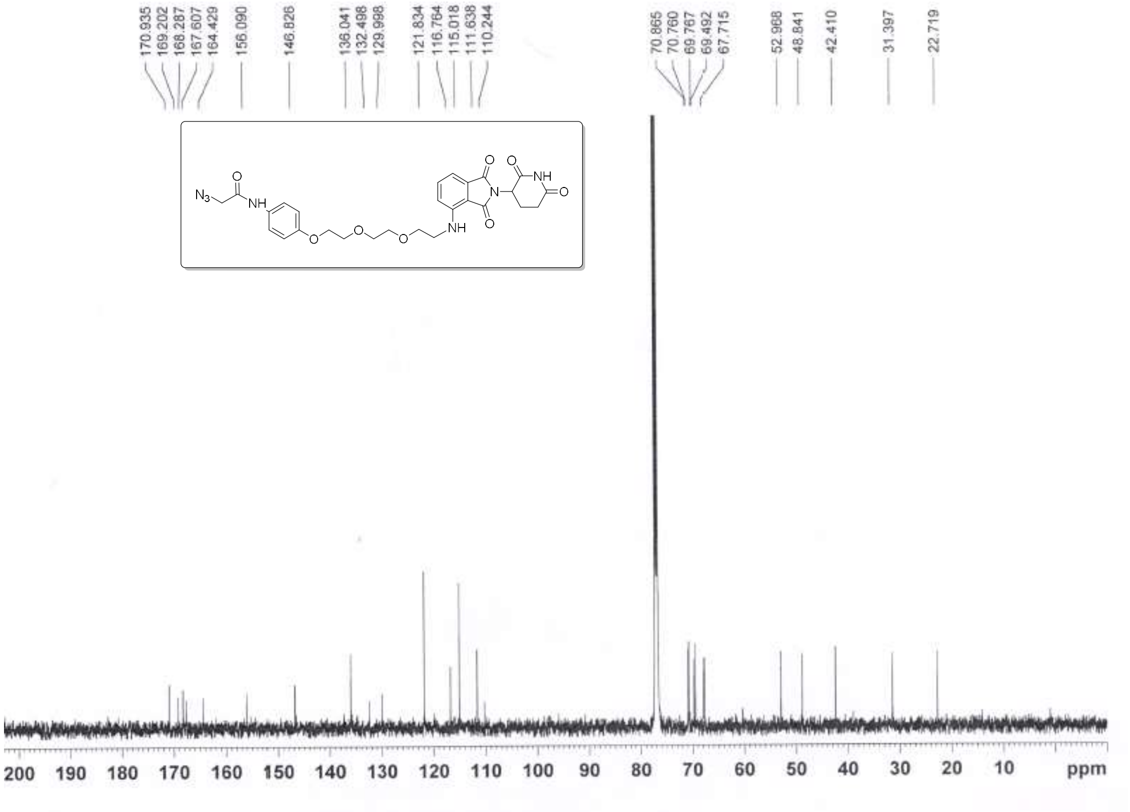

^{1}H NMR of compound **17** (400 MHz, $CDCl_3$)

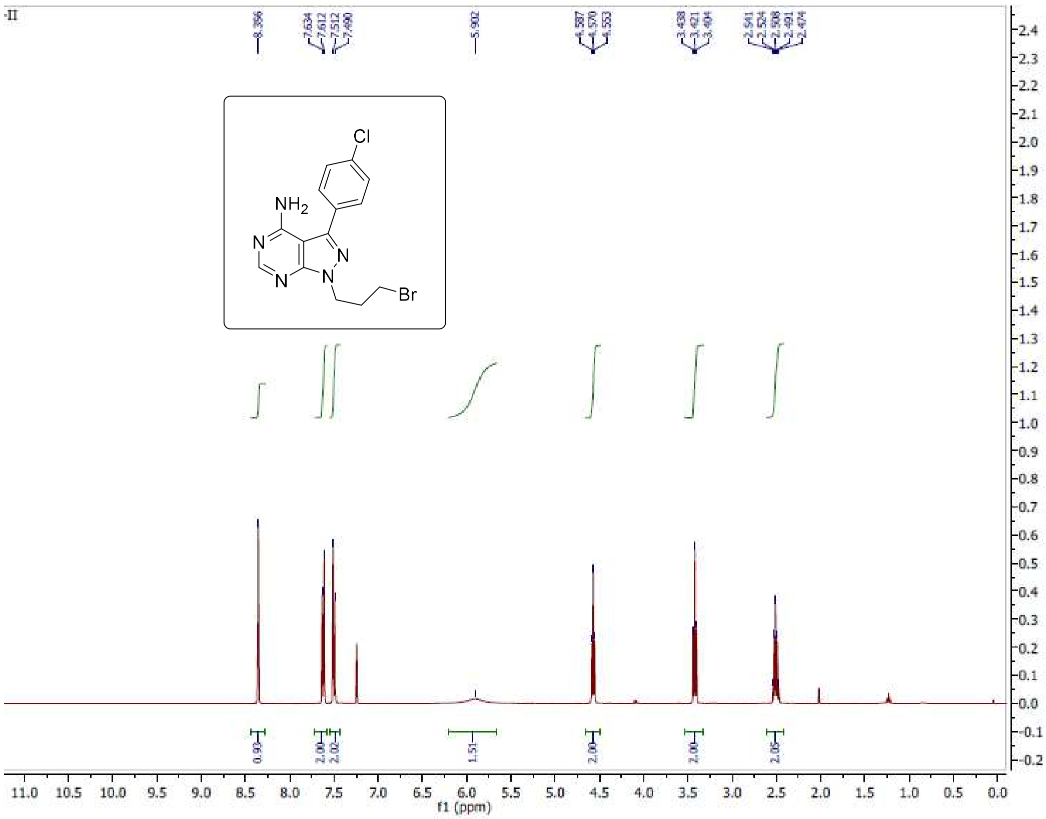

^{13}C NMR of compound **17** (125 MHz, $CDCl_3$)

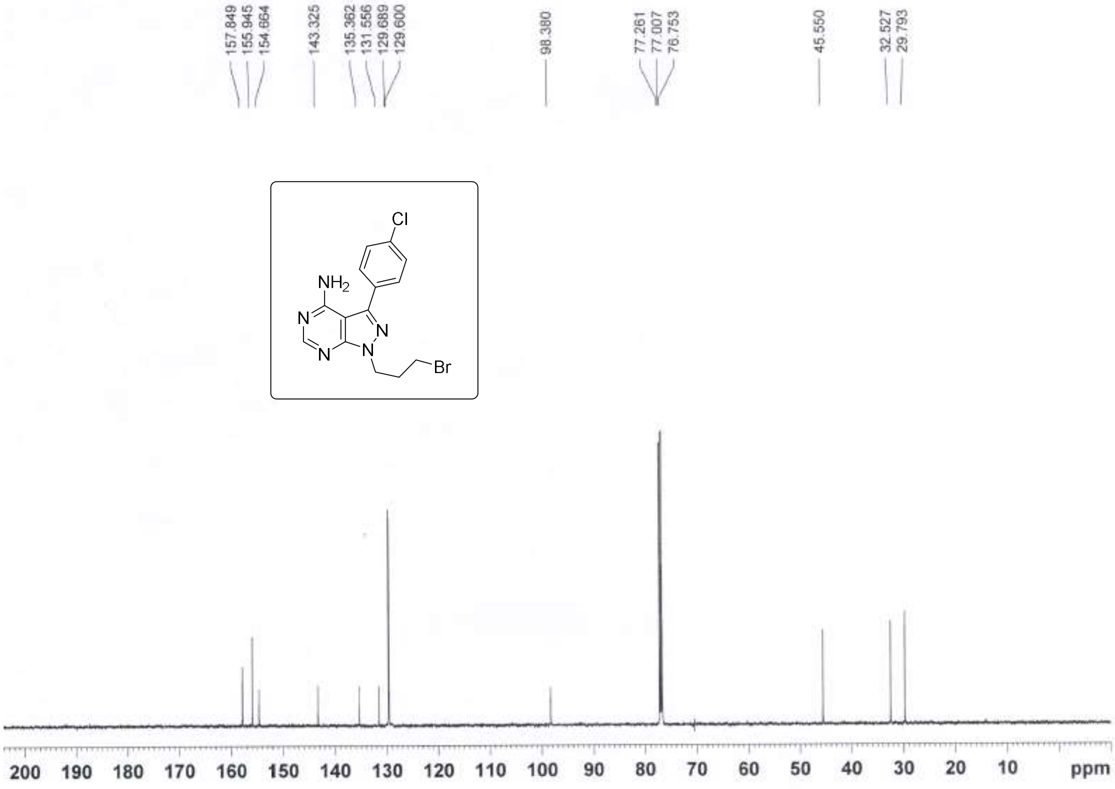

^{1}H NMR of compound **19** (400 MHz, $CDCl_3$)

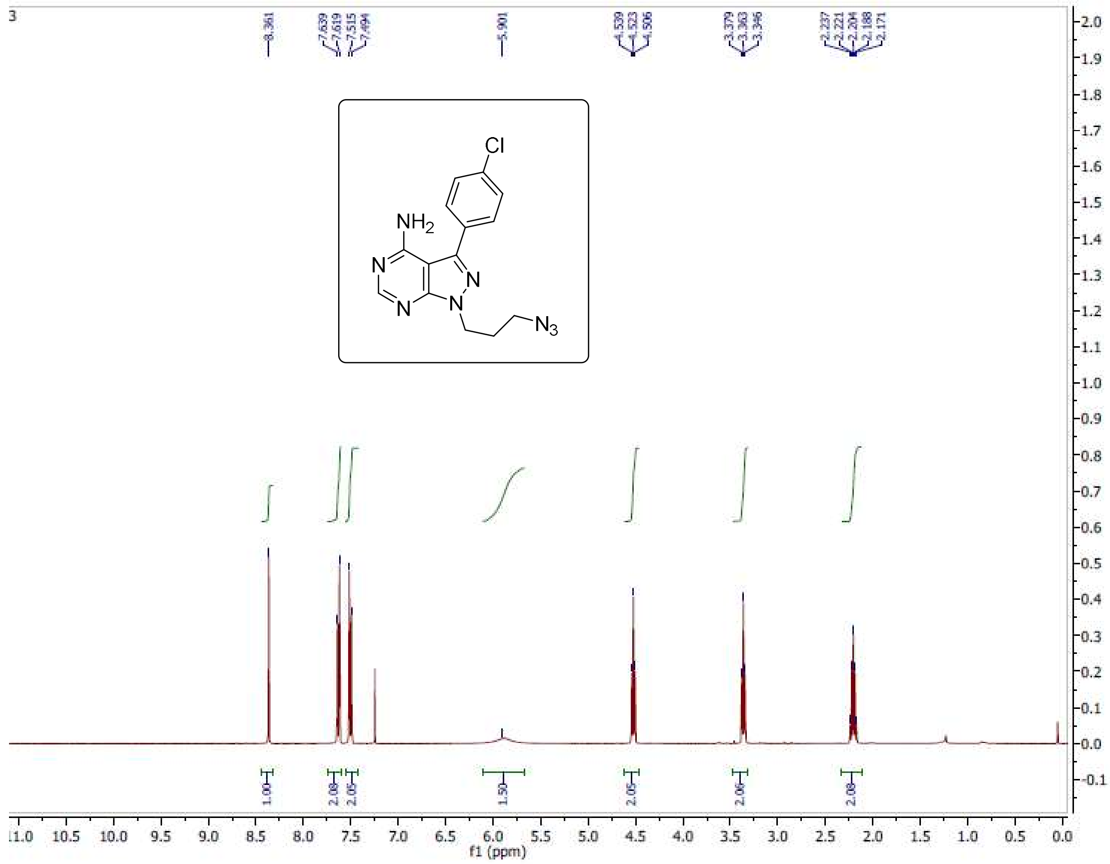

^{13}C NMR of compound **19** (125 MHz, $CDCl_3$)

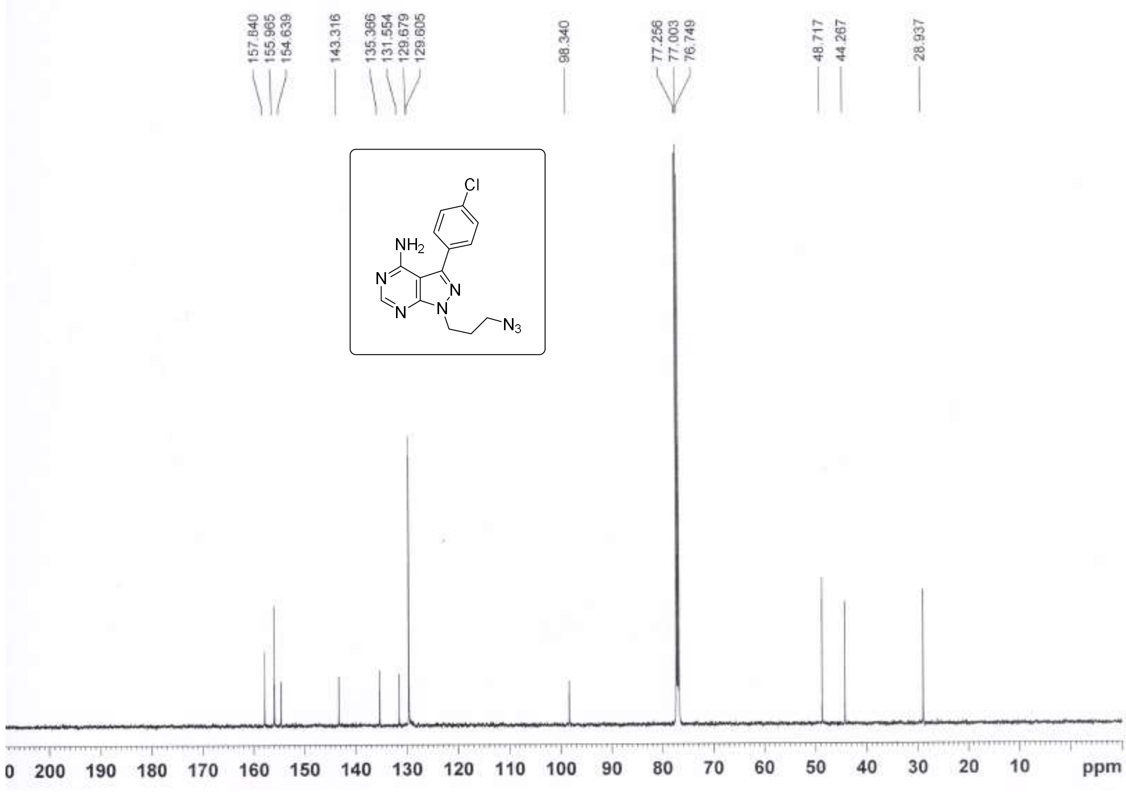

^{1}H NMR of compound **20a** (400 MHz, $CDCl_3$)

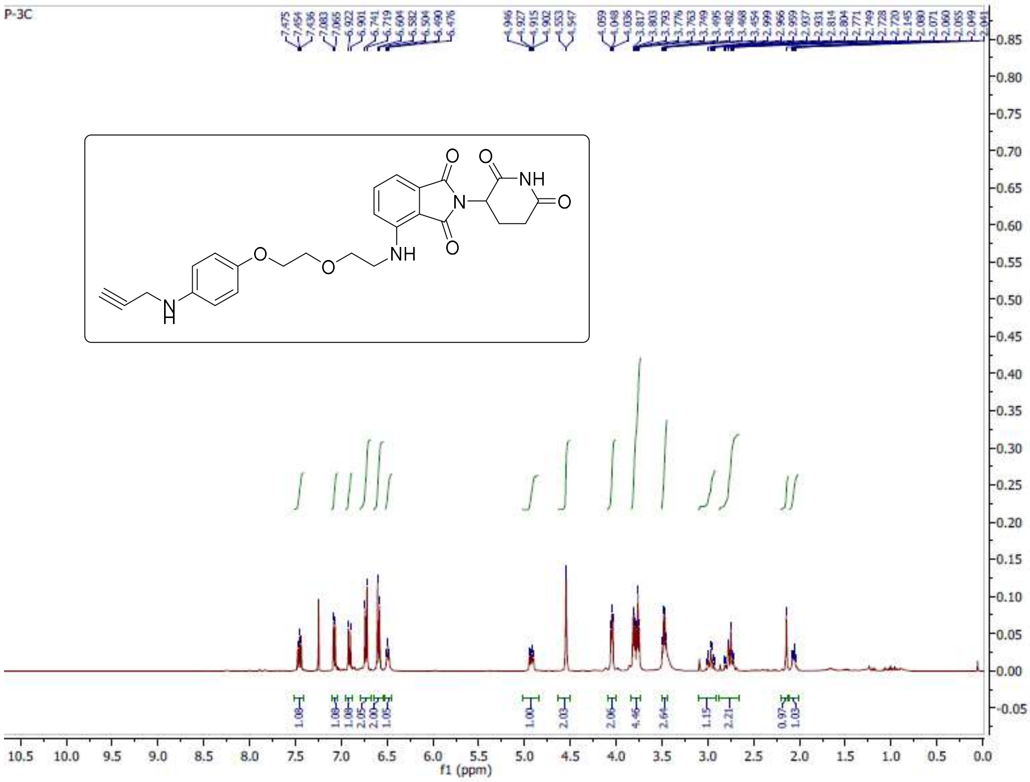

^{13}C NMR of compound **20a** (125 MHz, $CDCl_3$)

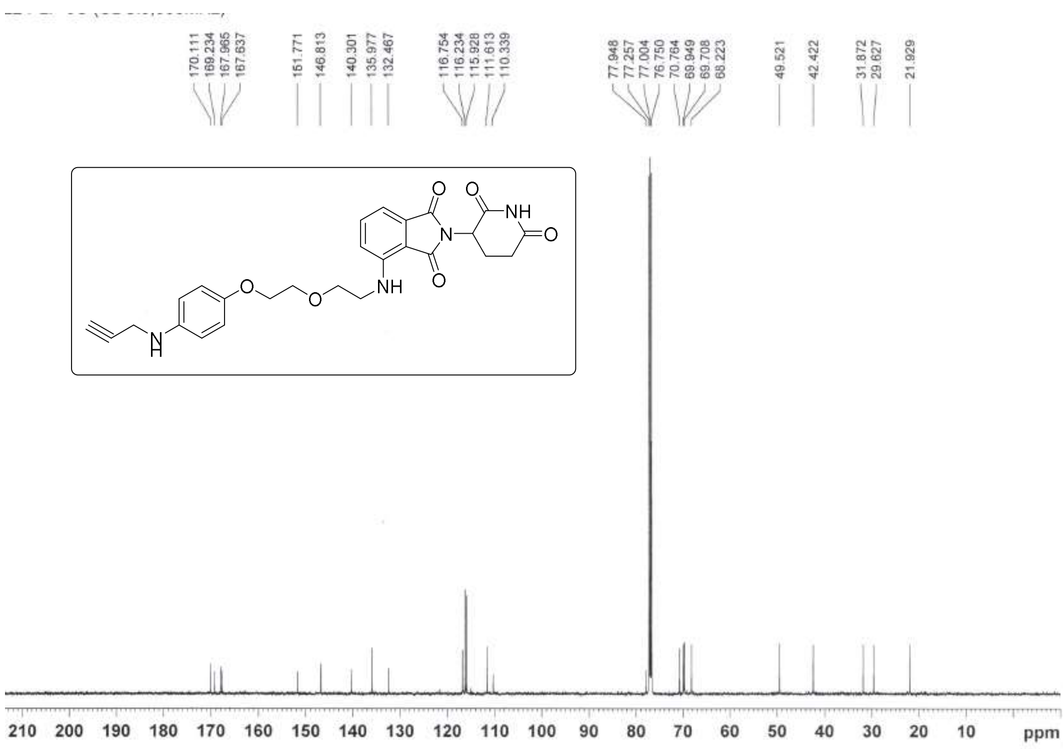

^{1}H NMR of compound **20b** (400 MHz, $CDCl_3$)

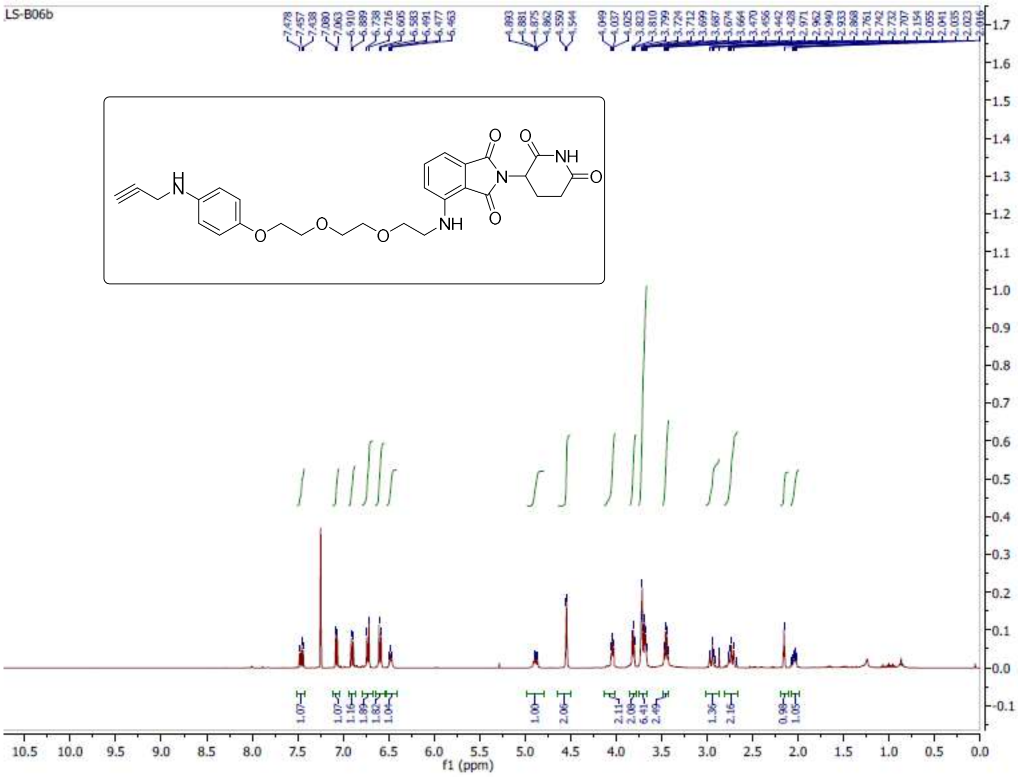

^{13}C NMR of compound **20a** (125 MHz, $CDCl_3$)

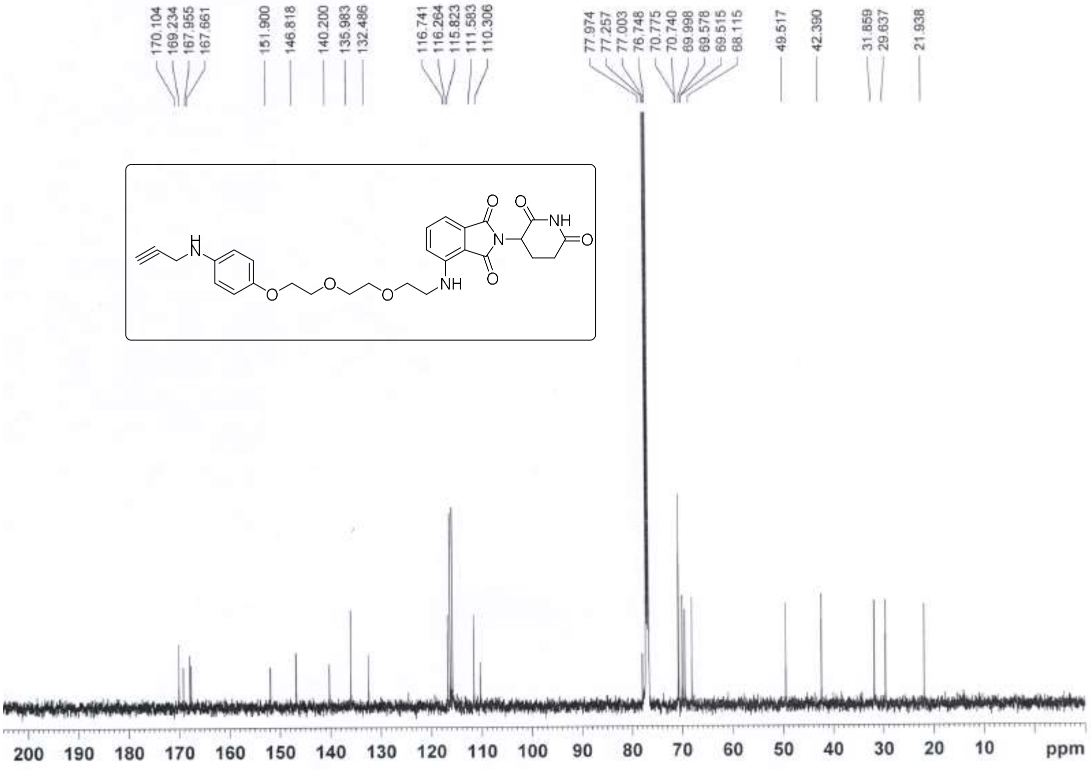

^{1}H NMR of compound **10a** (400 MHz, MeOD+$CDCl_3$)

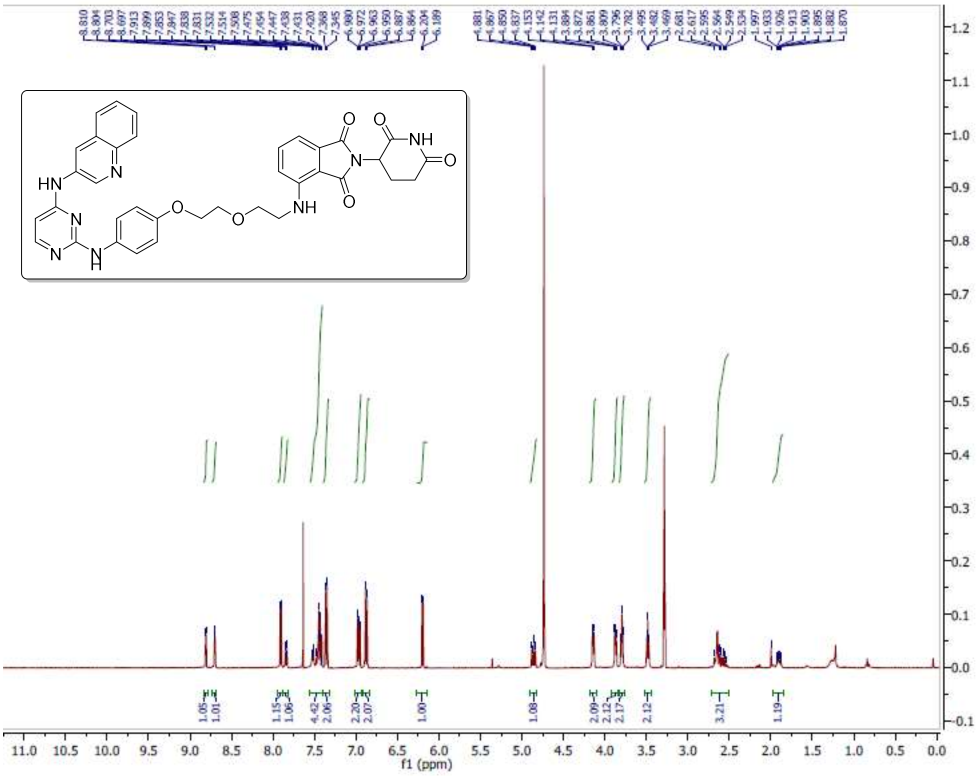

^{13}C NMR of compound **10a** (125MHz, MeOD+$CDCl_3$)

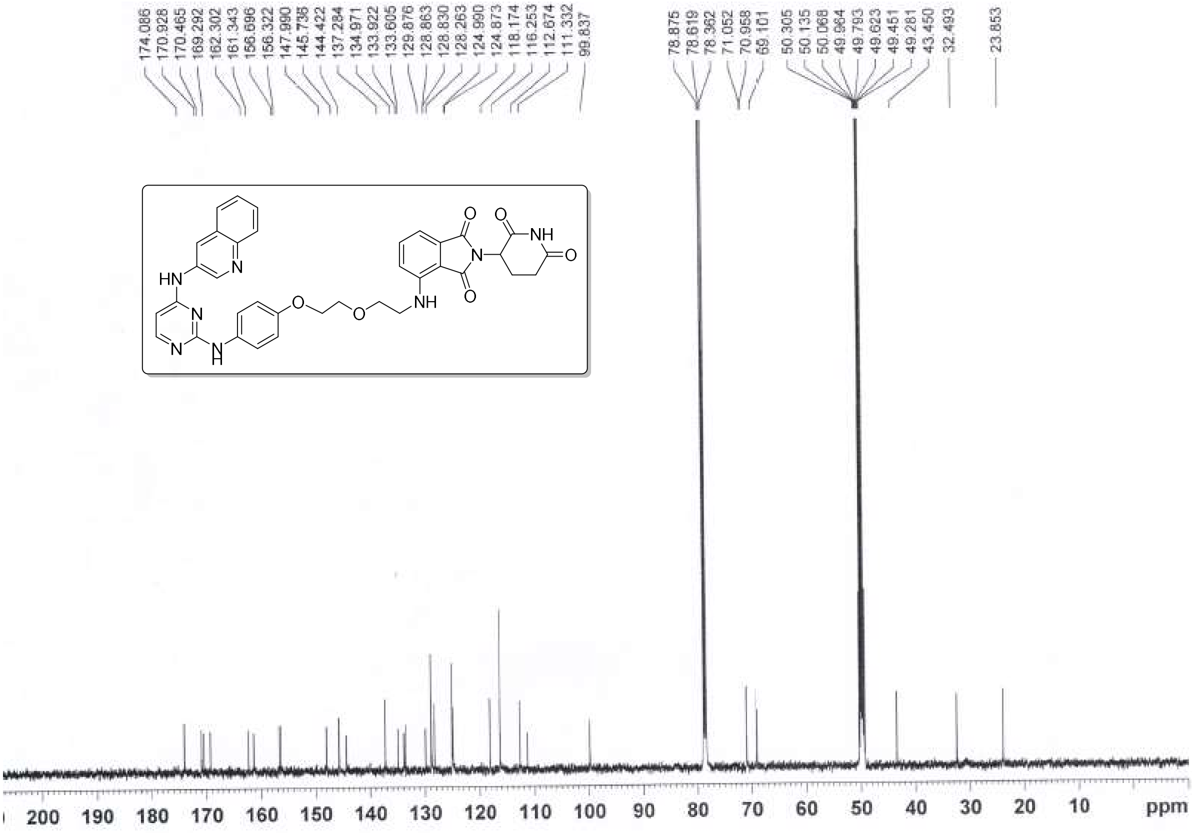

^{1}H NMR of compound **10b** (400 MHz, DMSO-d_6)

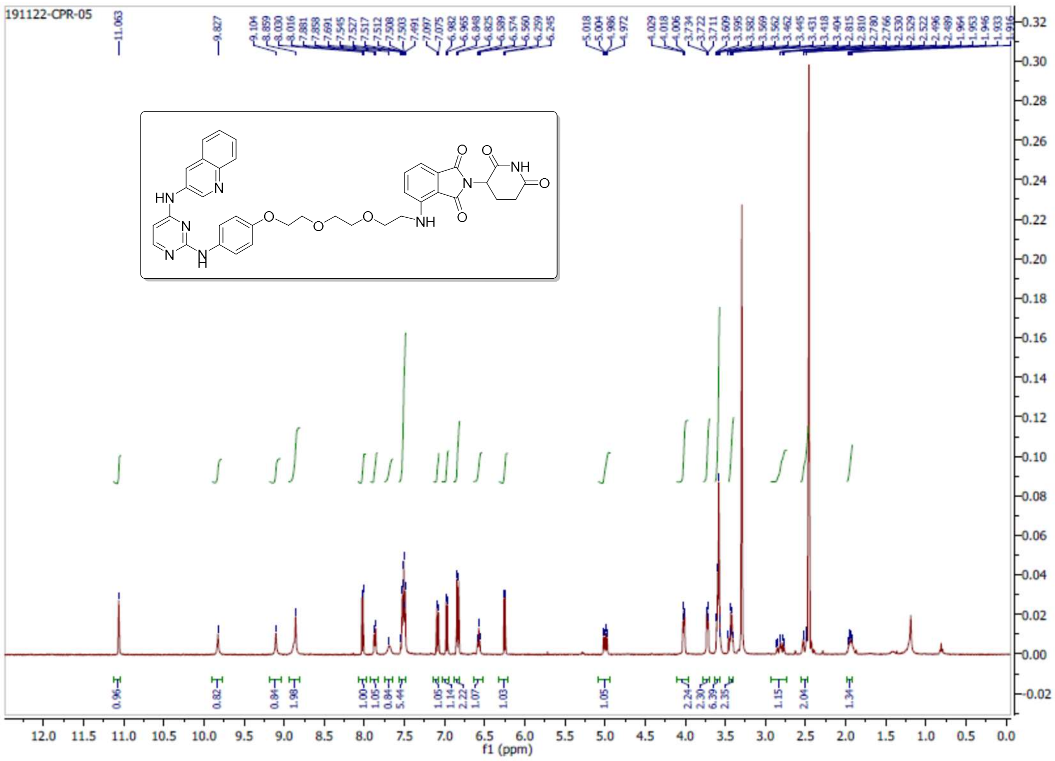

^{13}C NMR of compound **10b** (125 MHz, MeOD+ $CDCl_3$)

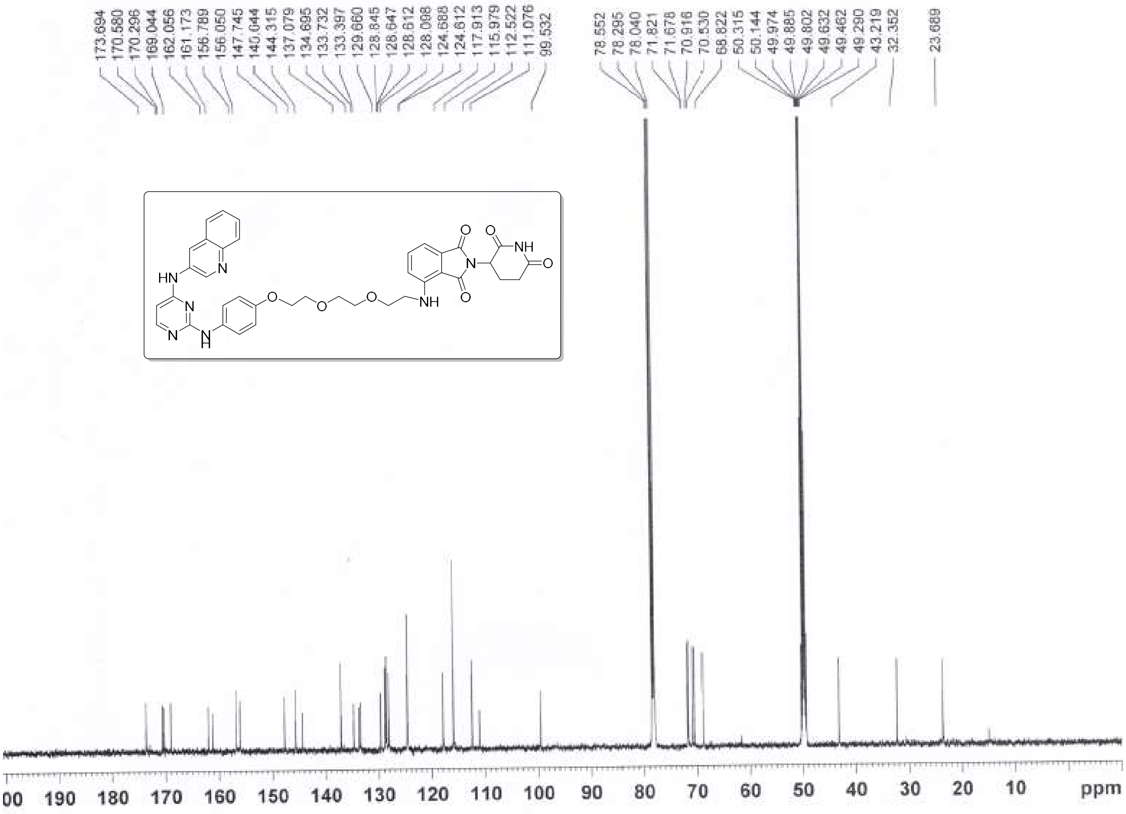

^{1}H NMR of compound **10c** (400 MHz, DMSO-d6)

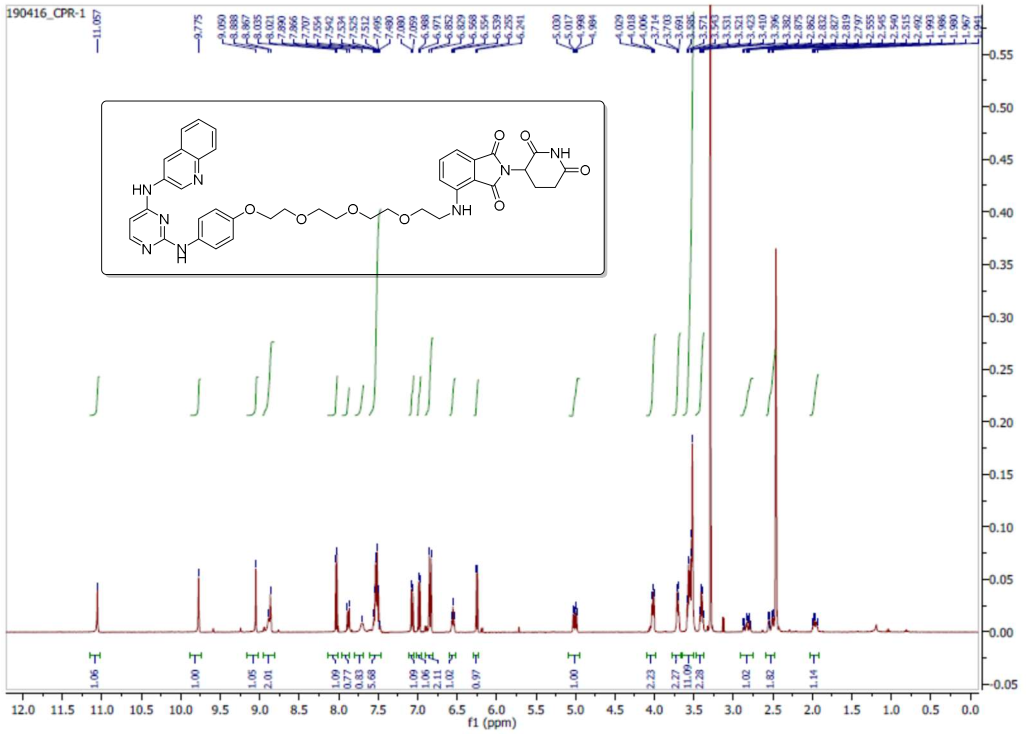

^{13}C NMR of compound **10c** (125 MHz, $CDCl_3$)

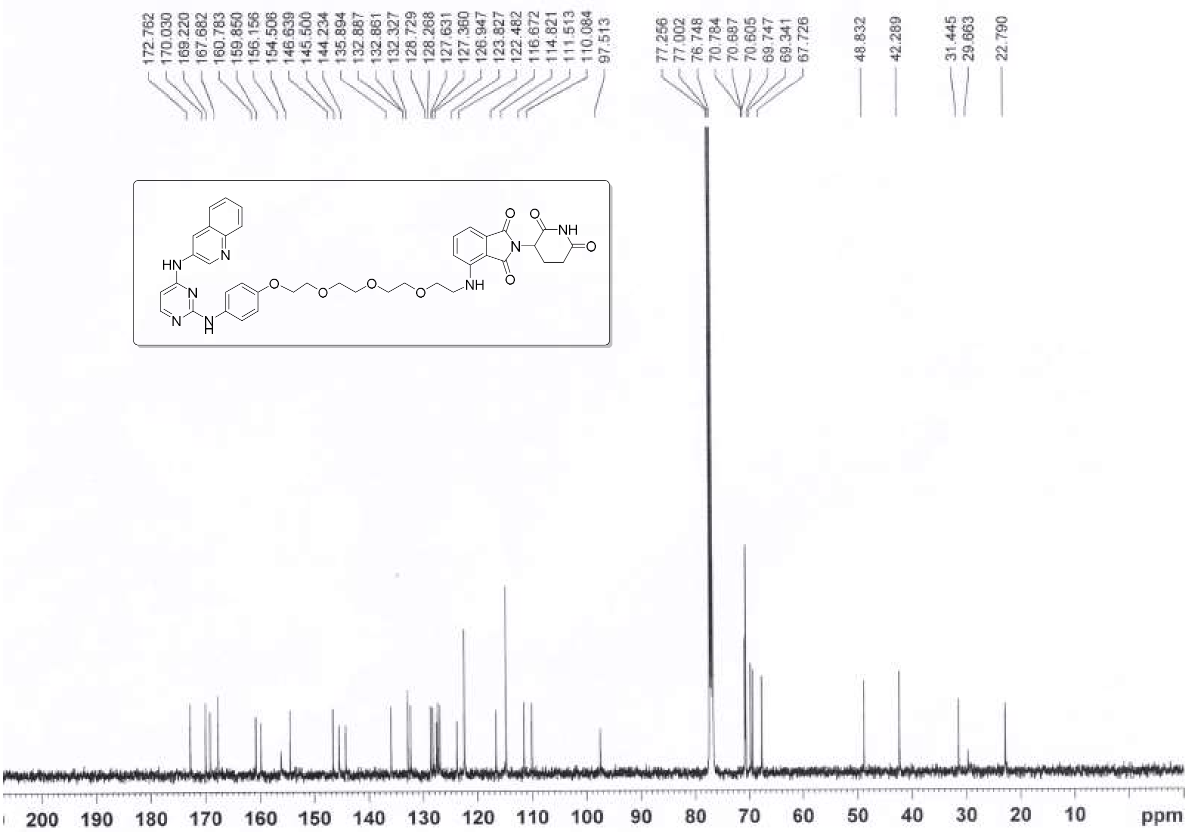

^{1}H NMR of compound **12a** (400 MHz, MeOD+$CDCl_3$)

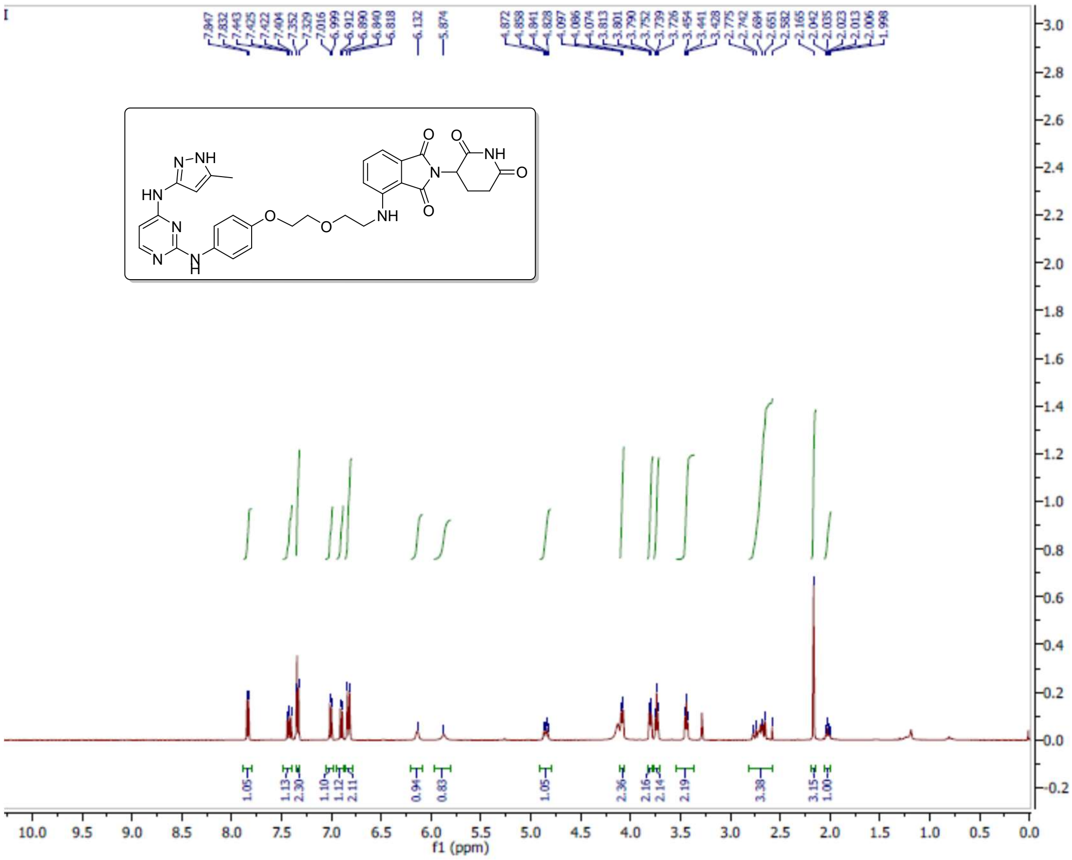

^{13}C NMR of compound **12a** (125 MHz, DMSO-d_6)

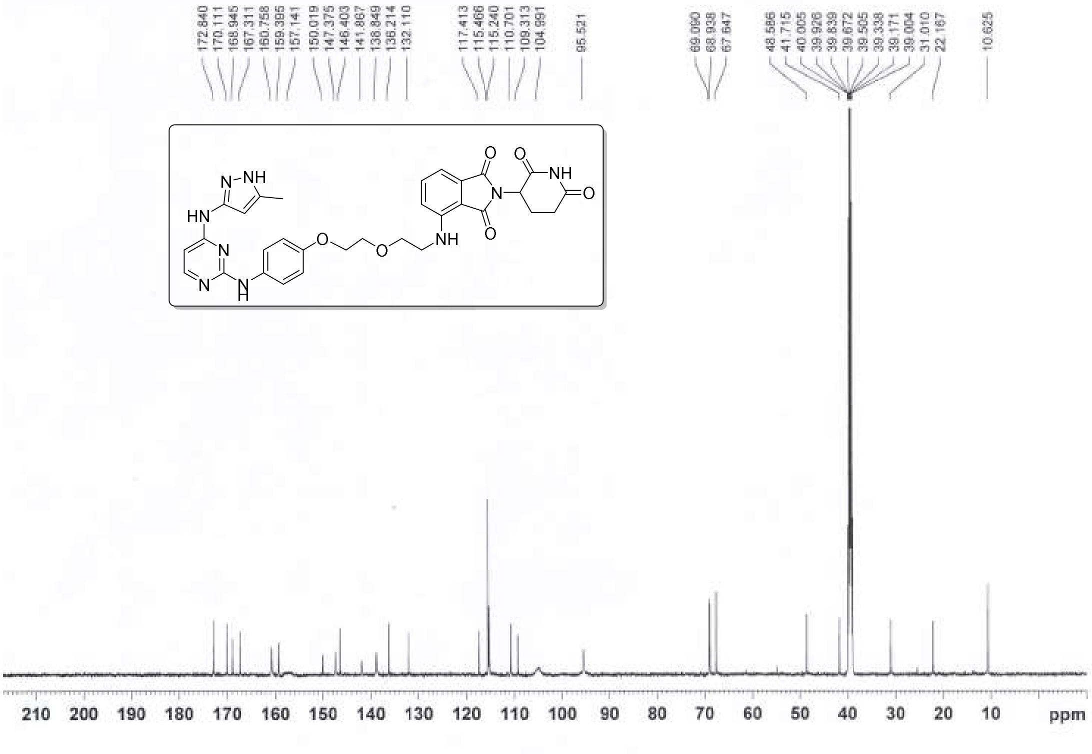

^{1}H NMR of compound **12b** (400 MHz, MeOD+$CDCl_3$)

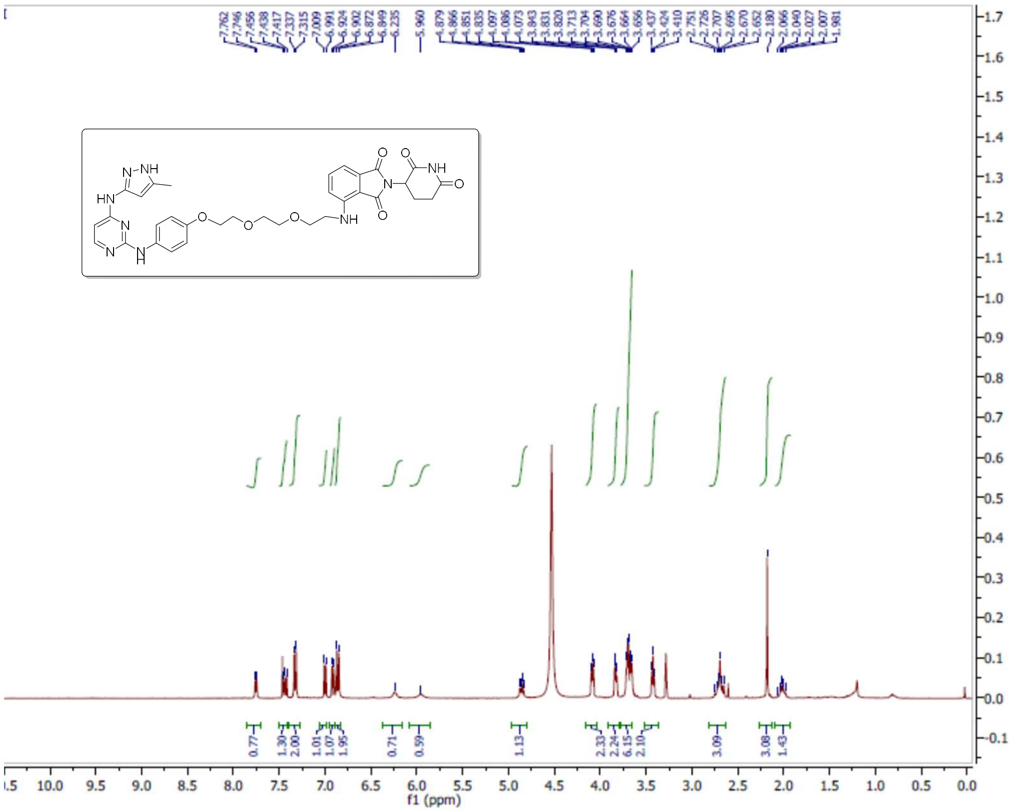

^{13}C NMR of compound **12b** (125 MHz, DMSO-d_6)

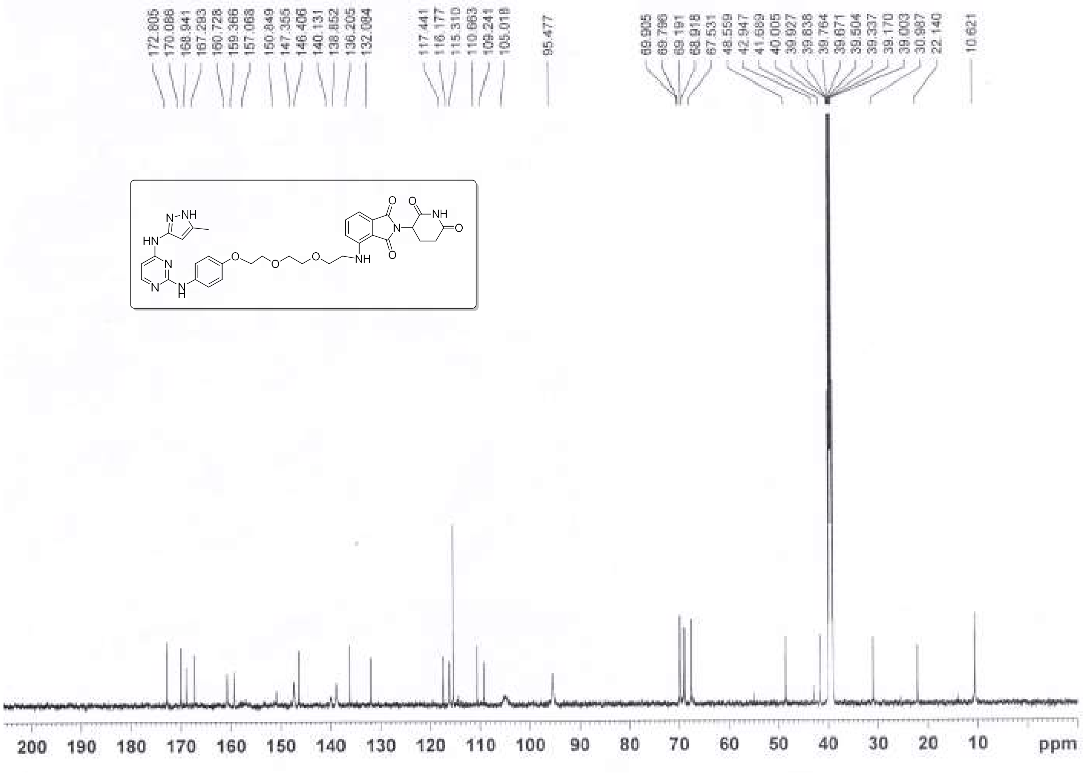

^1H NMR of compound **12c** (400 MHz, MeOD+ $CDCl_3$)

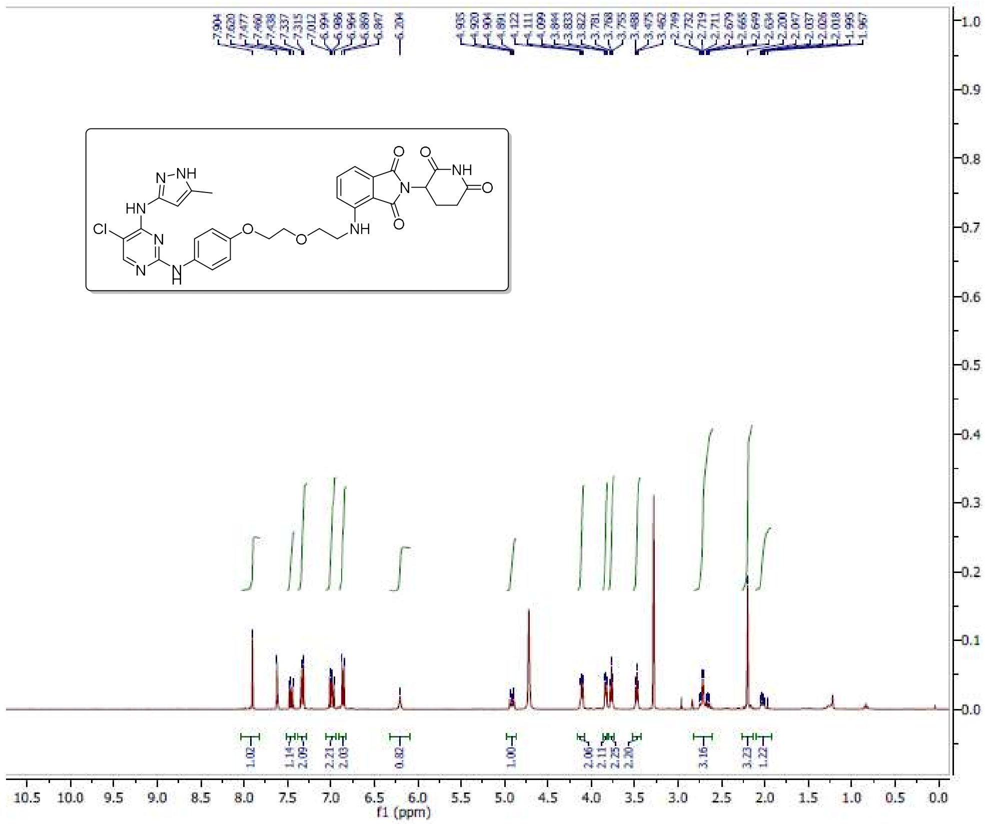

^{13}C NMR of compound **12c** (125 MHz, DMSO-d_6)

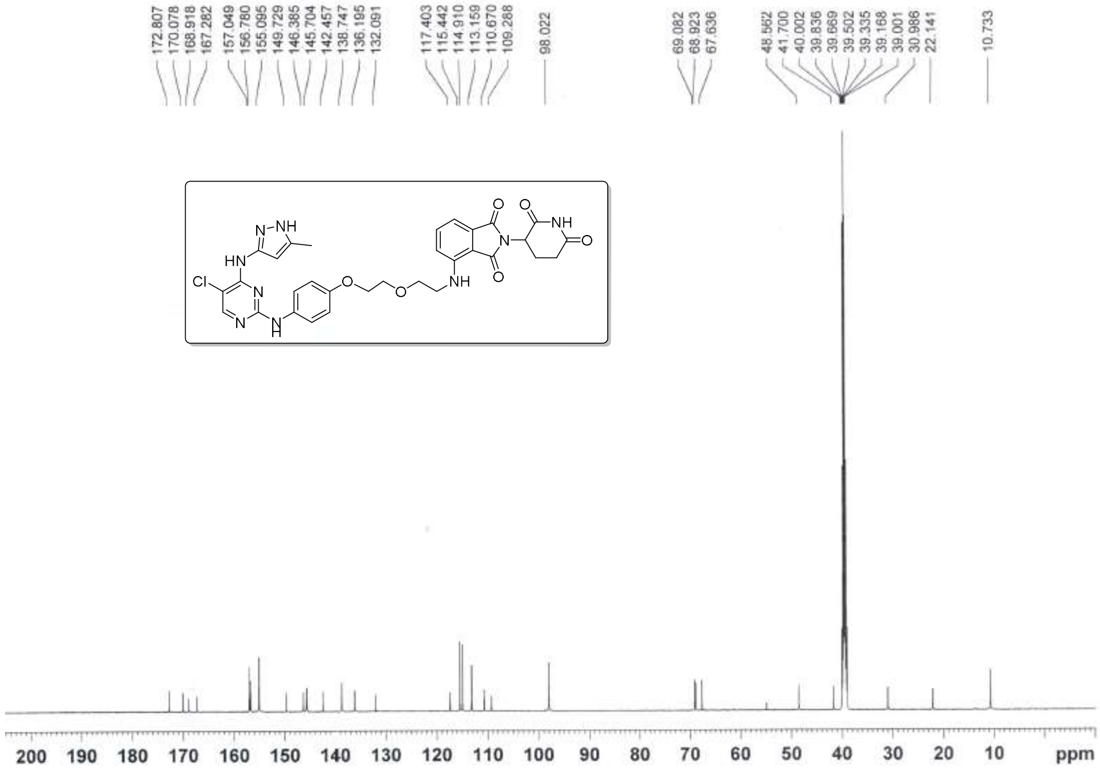

^{1}H NMR of compound **12d** (400 MHz, $CDCl_3$)

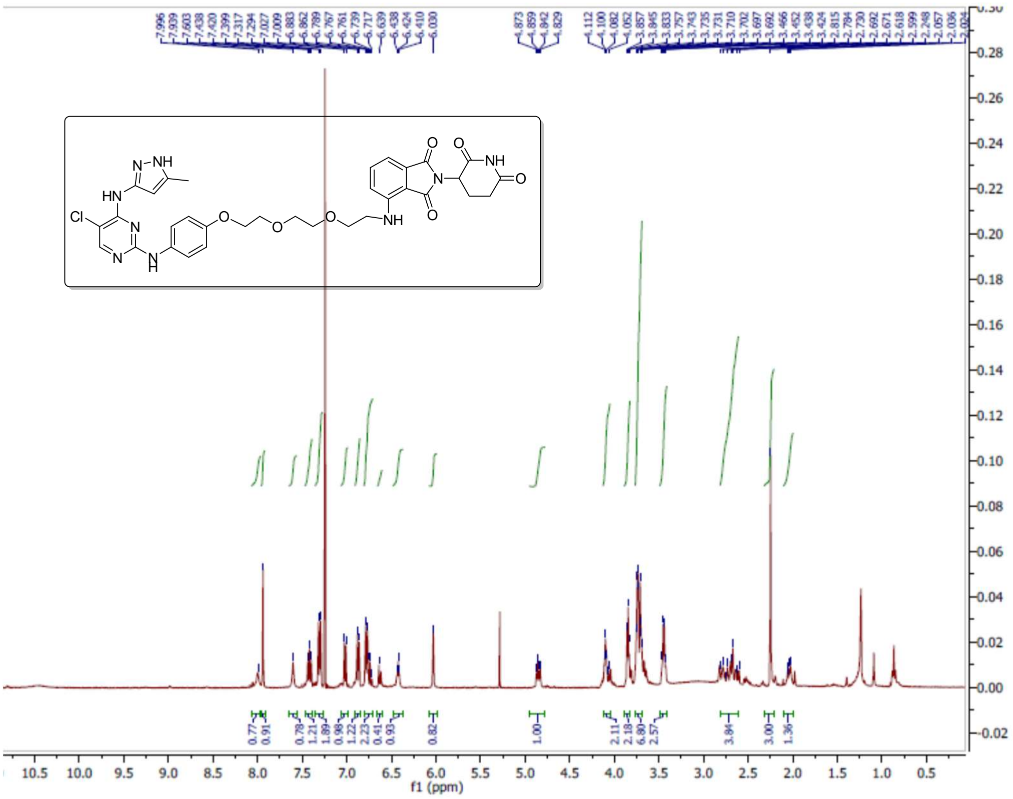

^{13}C NMR of compound **12d** (125 MHz, DMSO-d_6)

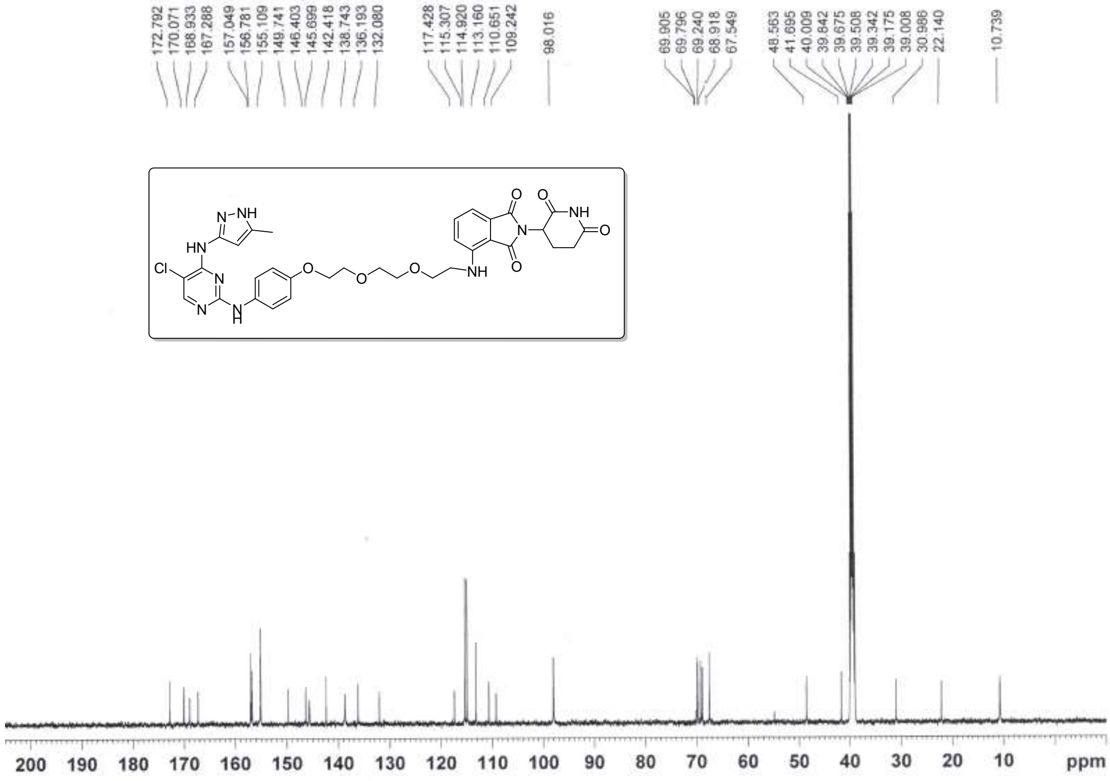

^{1}H NMR of compound **16a** (400 MHz, MeOD+ $CDCl_3$)

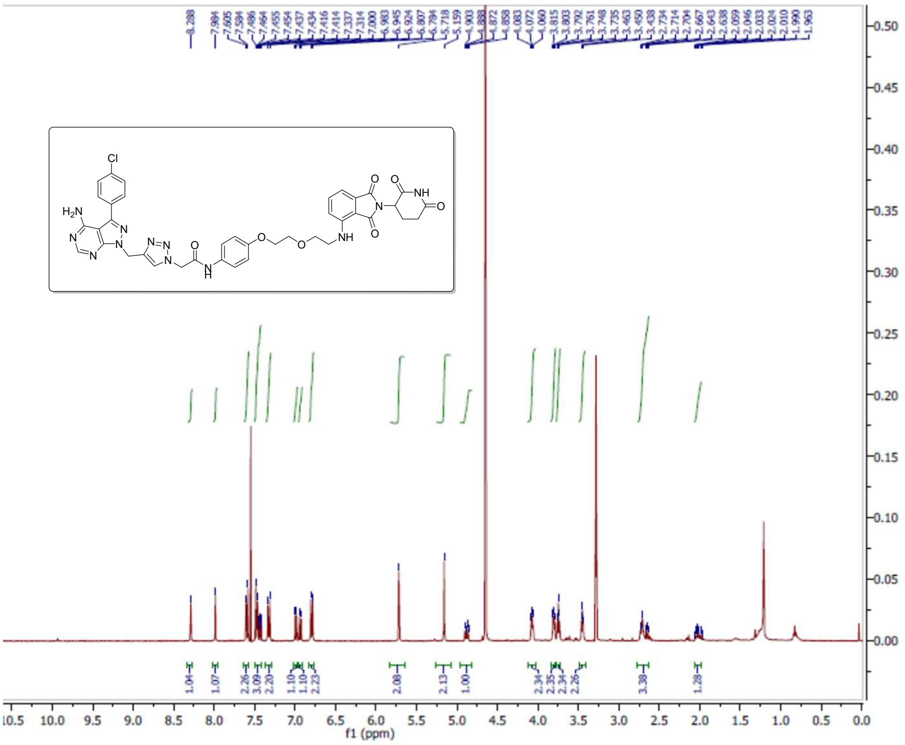

^{13}C NMR of compound **16a** (125 MHz, MeOD+ $CDCl_3$)

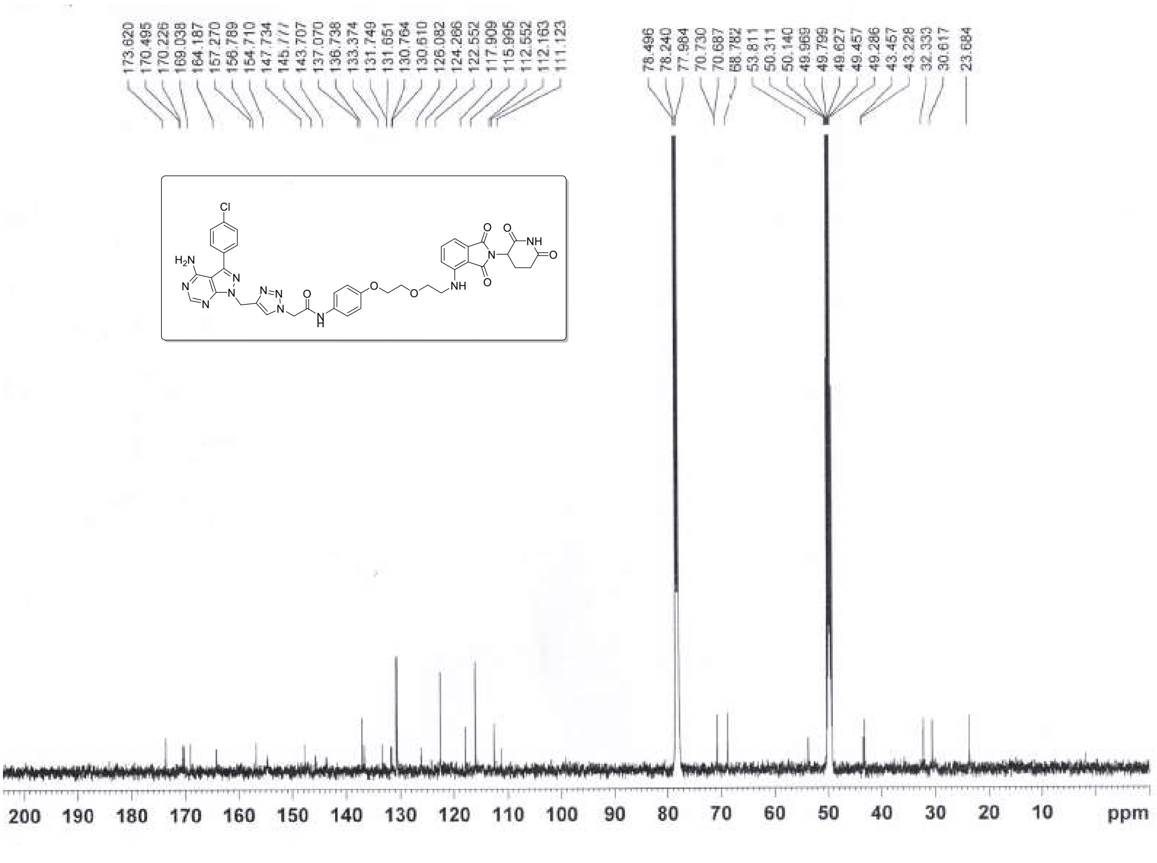

^{1}H NMR of compound **16b** (400 MHz, MeOD+ $CDCl_3$)

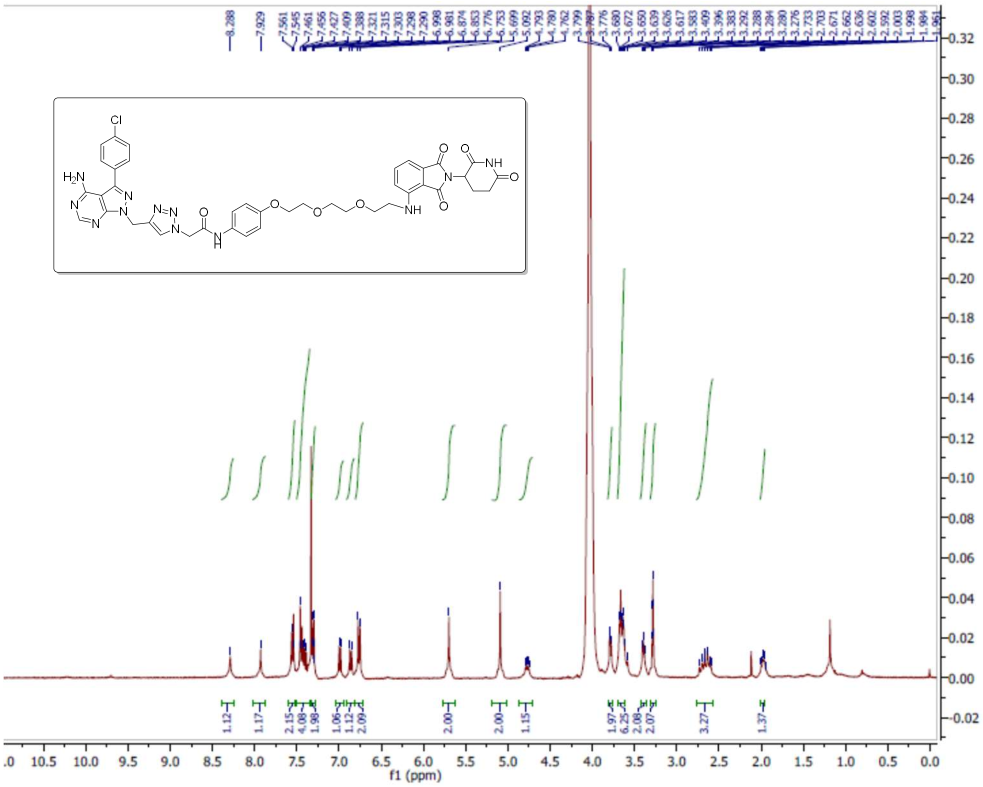

^{13}C NMR of compound **16b** (125 MHz, MeOD+ $CDCl_3$)

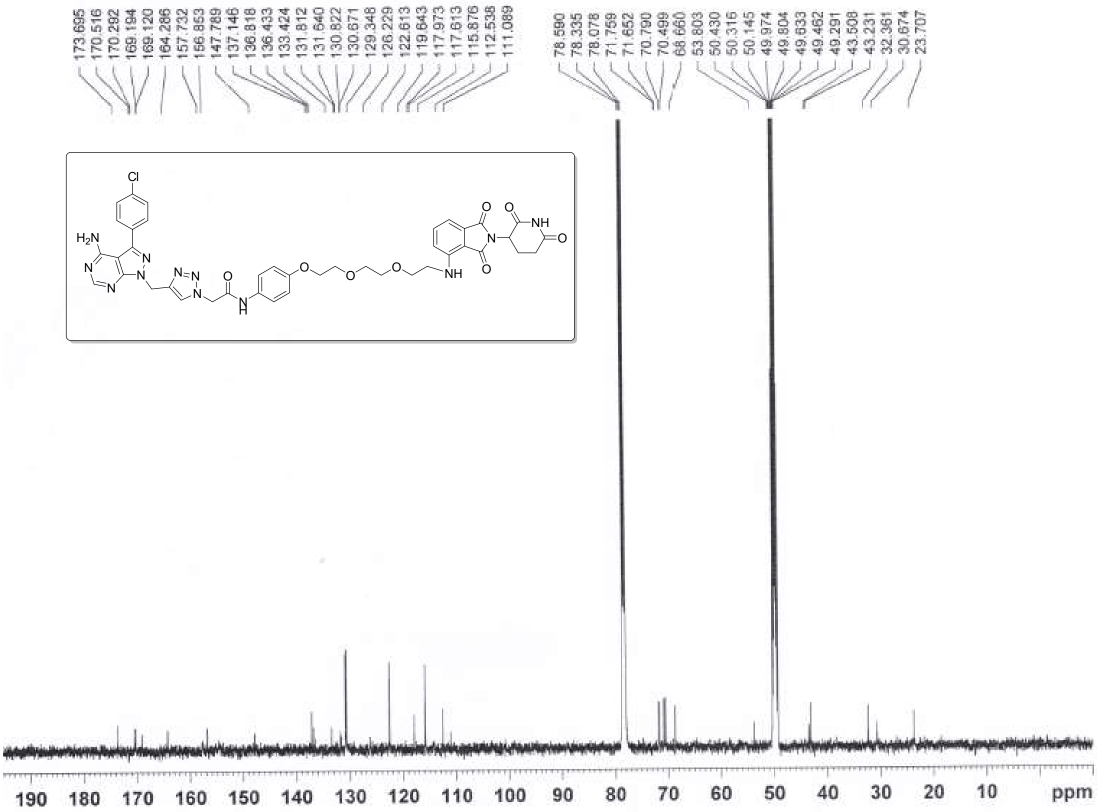

^{1}H NMR of compound **18a** (400 MHz, $CDCl_3$)

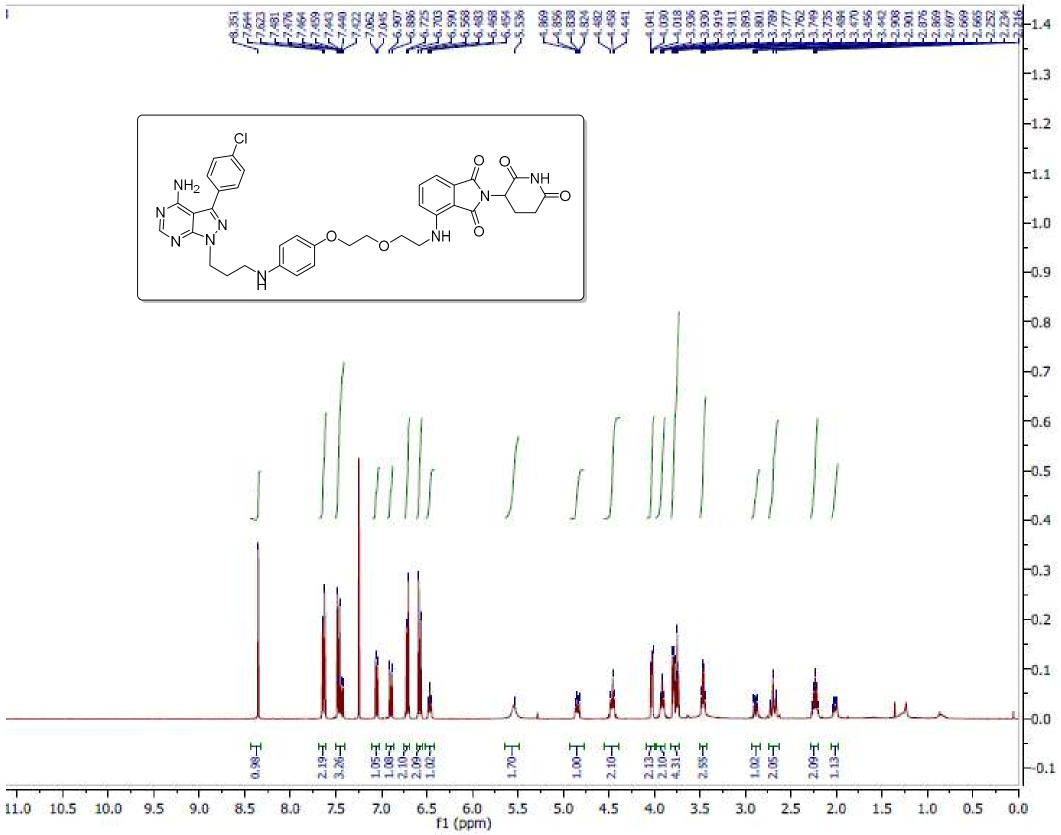

^{13}C NMR of compound **18a** (125 MHz, $CDCl_3$)

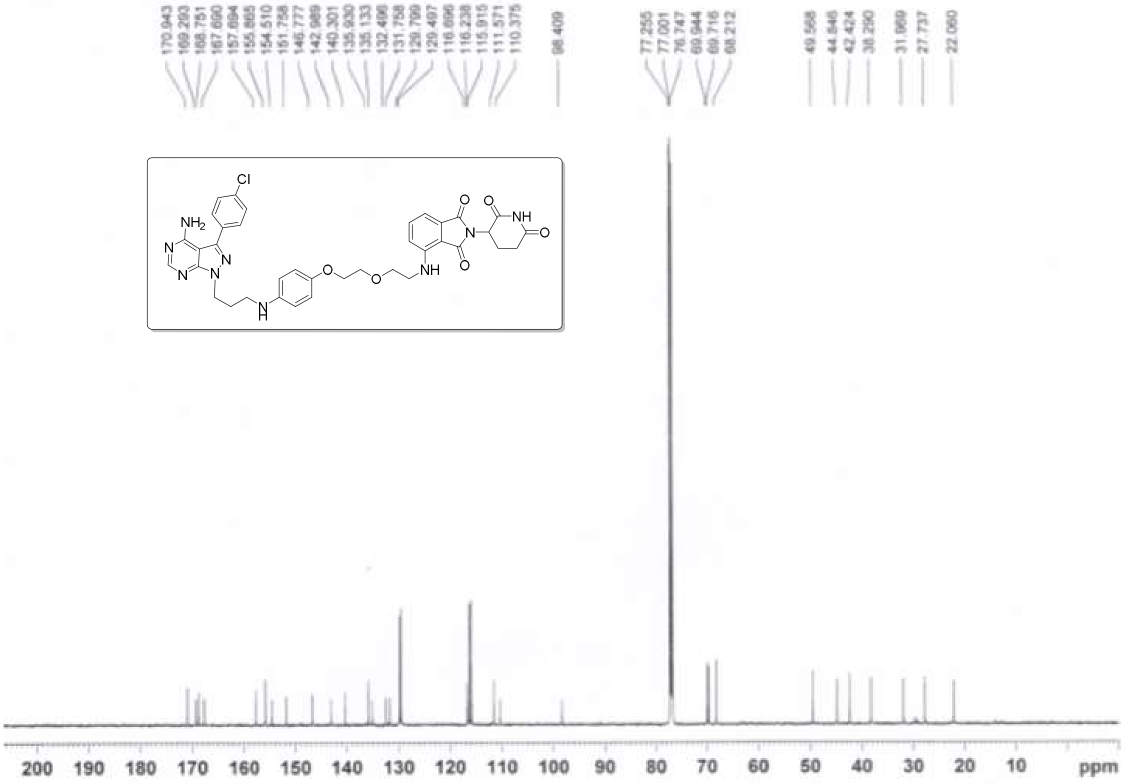

^{1}H NMR of compound **18b** (400 MHz, $CDCl_3$)

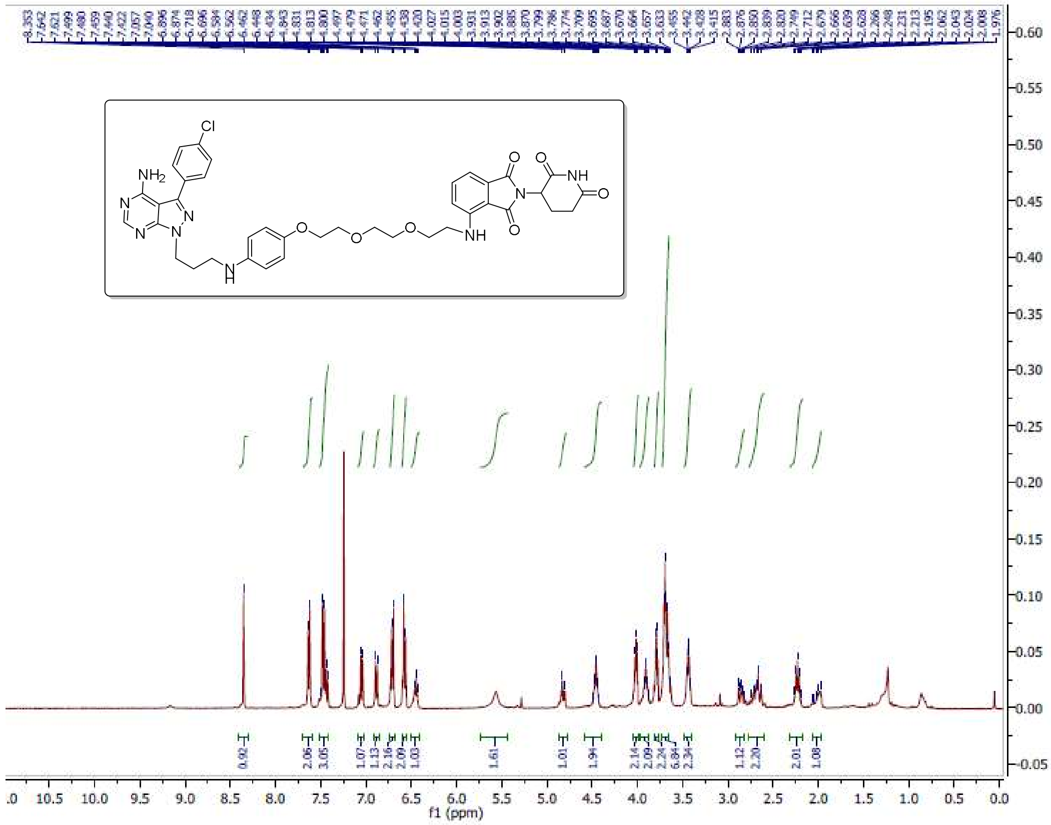

^{13}C NMR of compound **18b** (125 MHz, $CDCl_3$)

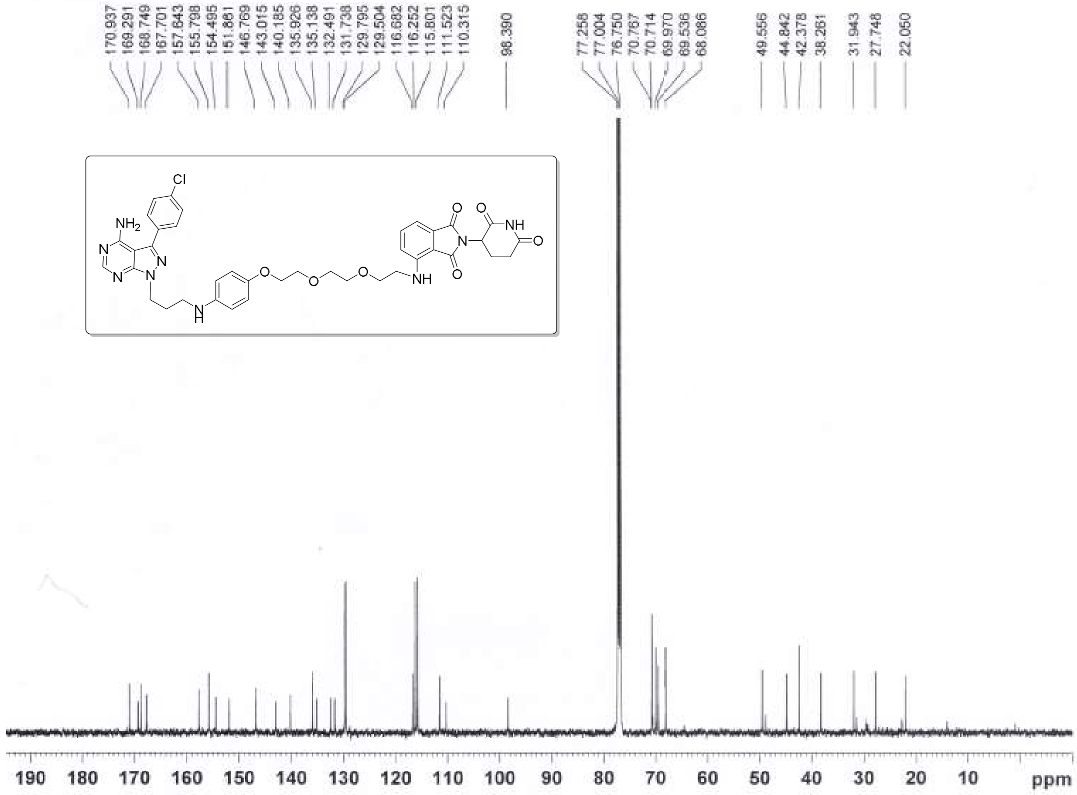

^{1}H NMR of compound **21a** (400 MHz, $CDCl_3$)

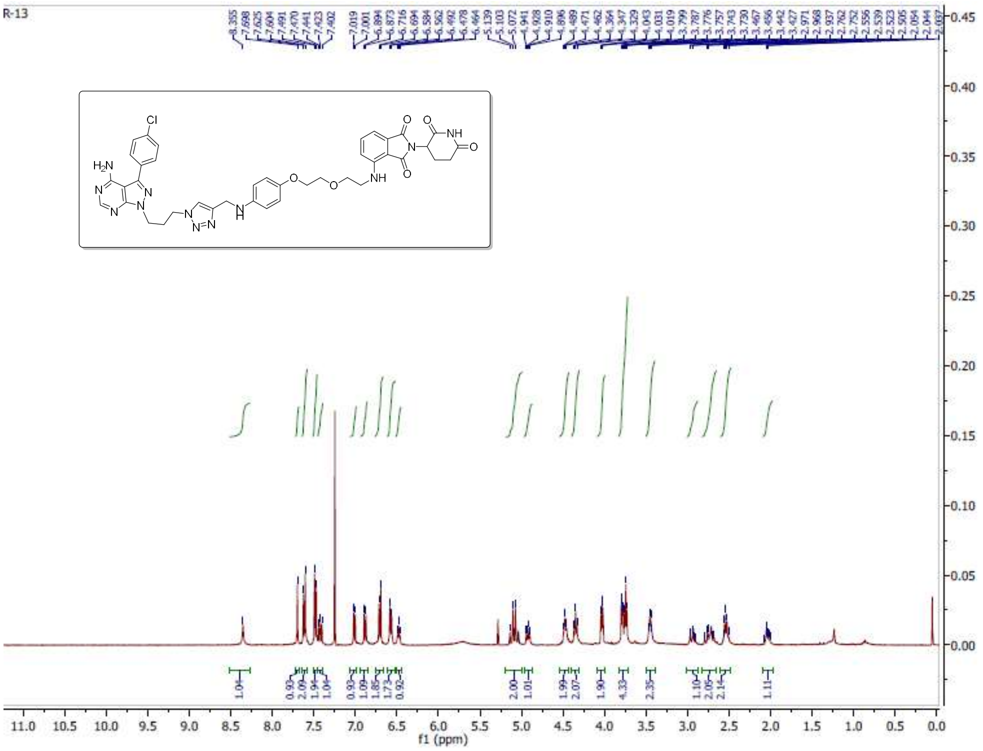

^{13}C NMR of compound **21a** (125 MHz, $CDCl_3$)

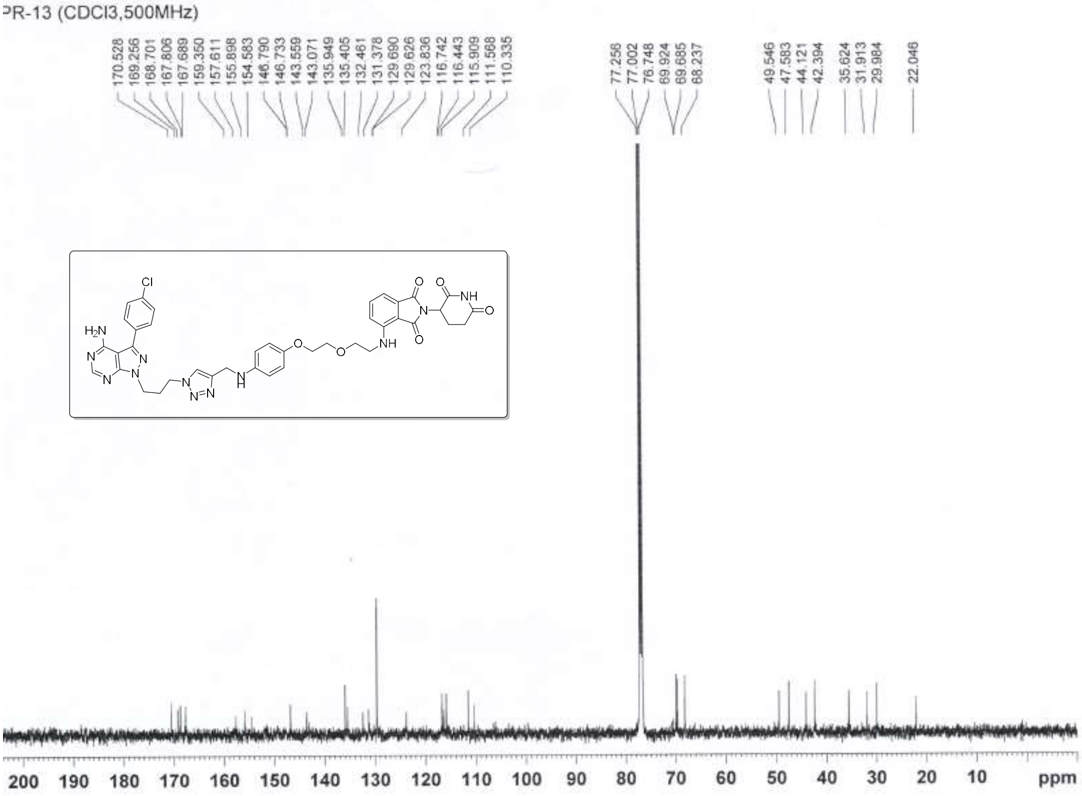

^{1}H NMR of compound **21b** (400 MHz, $CDCl_3$)

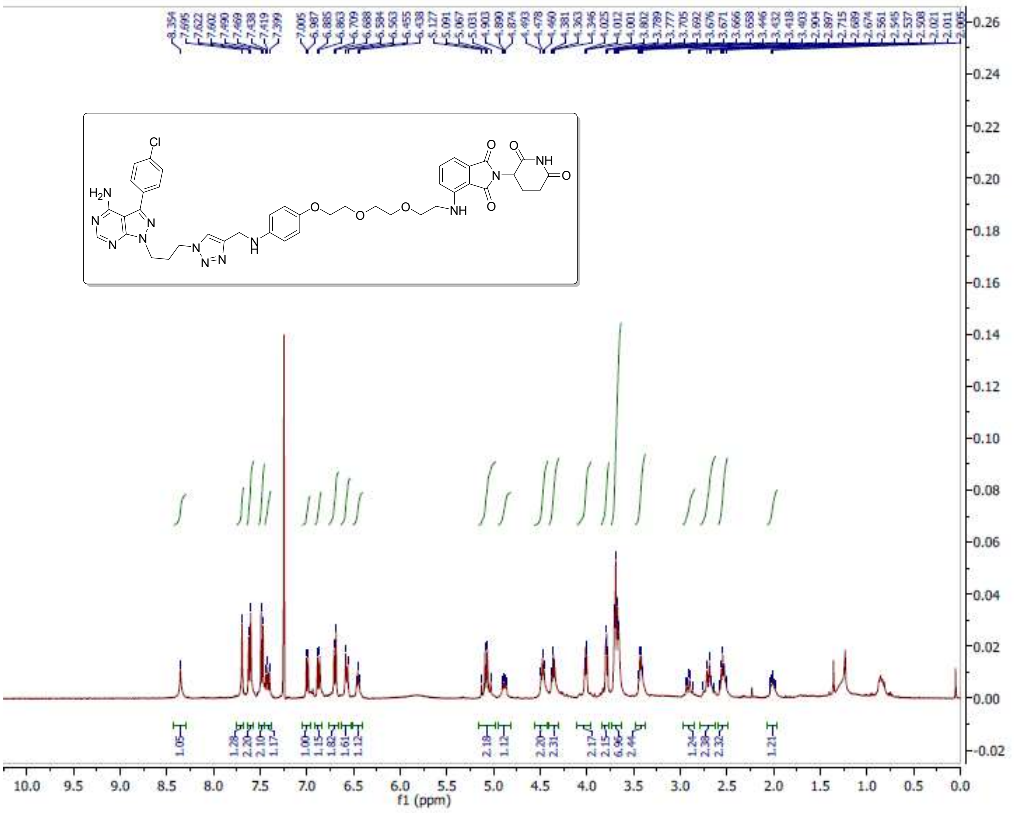

^{13}C NMR of compound **21b** (125 MHz, MeOD+ $CDCl_3$)

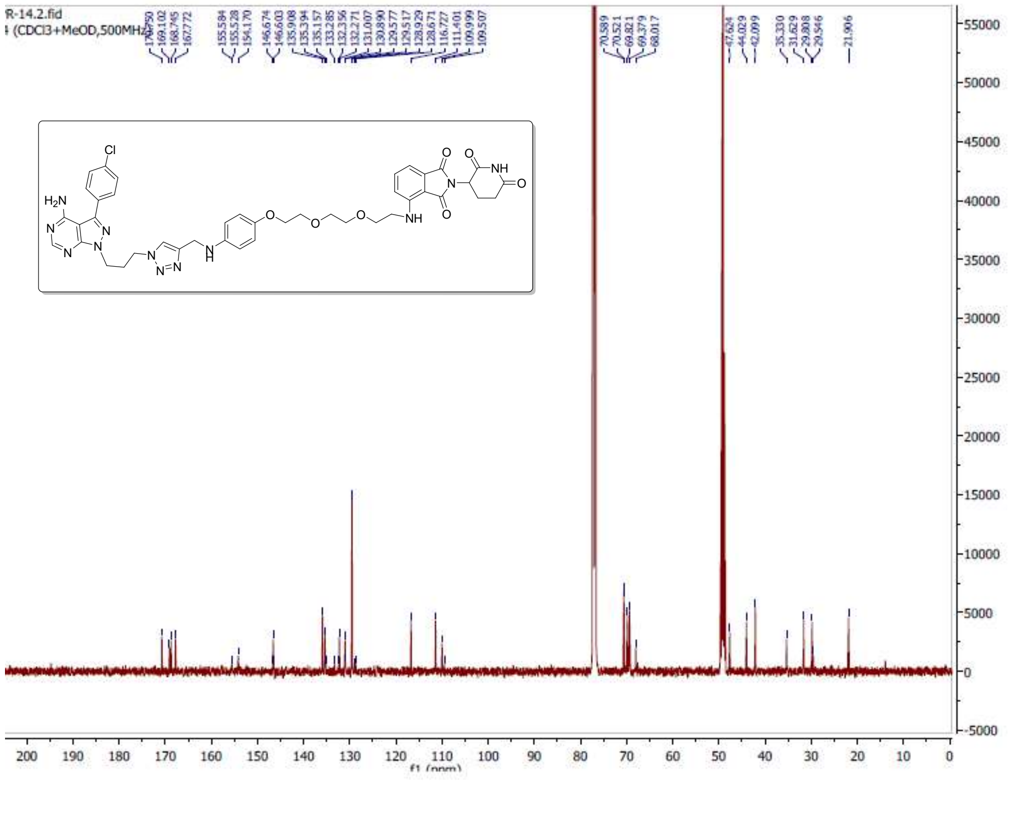

3. Reference:

1. Lu, J., Qian, Y., Altieri, M., Dong, H., Wang, J., Raina, K., Hines, J., Winkler, J.D., Crew, A.P., Coleman, K. *et al.* (2015) Hijacking the E3 Ubiquitin Ligase Cereblon to Efficiently Target BRD4. *Chemistry & biology*, **22**, 755-763.
2. Zhou, B., Hu, J., Xu, F., Chen, Z., Bai, L., Fernandez-Salas, E., Lin, M., Liu, L., Yang, C.Y., Zhao, Y. *et al.* (2018) Discovery of a Small-Molecule Degrader of Bromodomain and Extra-Terminal (BET) Proteins with Picomolar Cellular Potencies and Capable of Achieving Tumor Regression. *Journal of medicinal chemistry*, **61**, 462-481.
3. Buchanan, J.L., Newcomb, J.R., Carney, D.P., Chaffee, S.C., Chai, L., Cupples, R., Epstein, L.F., Gallant, P., Gu, Y., Harmange, J.C. *et al.* (2011) Discovery of 2,4-bis-arylamino-1,3-pyrimidines as insulin-like growth factor-1 receptor (IGF-1R) inhibitors. *Bioorg Med Chem Lett*, **21**, 2394-2399.
4. Engel, J., Richters, A., Getlik, M., Tomassi, S., Keul, M., Termathe, M., Lategahn, J., Becker, C., Mayer-Wrangowski, S., Grutter, C. *et al.* (2015) Targeting Drug Resistance in EGFR with Covalent Inhibitors: A Structure-Based Design Approach. *Journal of medicinal chemistry*, **58**, 6844-6863.
5. Ioannidis, S., Lamb, M.L., Wang, T., Almeida, L., Block, M.H., Davies, A.M., Peng, B., Su, M., Zhang, H.J., Hoffmann, E. *et al.* (2011) Discovery of 5-chloro-N2-[(1S)-1-(5-fluoropyrimidin-2-yl)ethyl]-N4-(5-methyl-1H-pyrazol-3-yl)p yrimidine-2,4-diamine (AZD1480) as a novel inhibitor of the Jak/Stat pathway. *Journal of medicinal chemistry*, **54**, 262-276.
6. Lee, H.J., Pham, P.C., Hyun, S.Y., Baek, B., Kim, B., Kim, Y., Min, H.Y., Lee, J. and Lee, H.Y. (2018) Development of a 4-aminopyrazolo[3,4-d]pyrimidine-based dual IGF1R/Src inhibitor as a novel anticancer agent with minimal toxicity. *Molecular cancer*, **17**, 50.
7. Kumar, A., Wang, Y., Lin, X., Sun, G. and Parang, K. (2007) Synthesis and evaluation of 3-phenylpyrazolo[3,4-d]pyrimidine-peptide conjugates as Src kinase inhibitors. *ChemMedChem*, **2**, 1346-1360.
